# Metal-Free Trifluoromethylthiolation of Arylazo Sulfones

**DOI:** 10.1021/acs.joc.0c02669

**Published:** 2020-12-22

**Authors:** Ankun Li, Yuxuan Li, Junjie Liu, Jingqi Chen, Kui Lu, Di Qiu, Maurizio Fagnoni, Stefano Protti, Xia Zhao

**Affiliations:** † Tianjin Key Laboratory of Structure and Performance for Functional Molecules, College of Chemistry, Tianjin Normal University, Tianjin 300387, People’s Republic of China; ‡ PhotoGreen Lab, Department of Chemistry, University of Pavia, V. Le Taramelli 12, Pavia 27100, Italy; § College of Biotechnology, Tianjin University of Science & Technology, Tianjin 300457, China

## Abstract

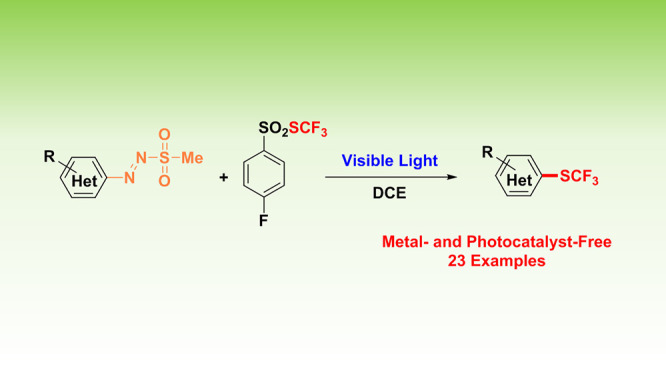

A visible-light-driven protocol for the synthesis of aryl trifluoromethyl thioethers under photocatalyst- and metal-free conditions has been pursued. The procedure exploits the peculiar properties of arylazo sulfones (having electron-rich or electron-poor substituents on the (hetero)aromatic ring) as photochemical precursors of aryl radicals and *S*-trifluoromethyl arylsulfonothioates as easy-to-handle trifluoromethylthiolating agents.

The formation of an Ar–SCF_3_ bond is important in life sciences for the synthesis of bioactive molecules. In fact the SCF_3_ group, when present in an aromatic compound, strongly affected its physicochemical properties, mostly its lipophilicity. The latter is a key parameter in drug design since the bioavailability of the drug is enhanced when lipophilicity increases.^1^ In this case, a combined effect of the presence of fluorine atoms^2^ with heteroatoms imparts a good lipophilicity as witnessed by the high values of the Hansch parameters (π_R_ = 1.44).^3^ Some drugs, such as the antiprotozoal agent Toltrazuril and the stimulant amphetamine Tiflorex, contain the Ar–SCF_3_ moiety. Accordingly, a more reliable synthetic procedure in the forging of the Ar–S bond could increase a wider application of trifluoromethylthiolated aromatics in medicinal chemistry. In the last years, several synthetic protocols for the introduction of a SCF_3_ group in a (hetero)aromatic core have been developed.^4^ An interesting approach is the direct trifluoromethylthiolation of (hetero)arenes, but only certain electron-rich derivatives led to the desired product in a clean fashion, avoiding the concomitant formation of undesired regioisomers (Scheme 1a).^5^ Thus, several approaches have been developed by replacing an aromatic substituent with a SCF_3_ group making use of a metal catalyst (e.g., Cu,^6^ Ni,^7^ Pd,^8^ and Ag^9^). The reaction is, in most cases, an ipso-substitution that starts from aryl diazonium salts,^6a,6d,6h^ aryl halides,^6c,7a,7c,7d,9^ aryl boronic acids,^6b−6g^ aryltrifluoroborates,^6e^ di(hetero)aryl-λ^3^-iodanes,^6f^ aryl sulfonates,^7b^ and arylmercaptodifluoroacetic acids (Scheme 1b).^8a^ Metal-free alternatives, however, are limited to the alkylation of aryl sulfides by using CF_3_I under basic conditions^10^ (Scheme 1c) except the trifluoromethylation of diaryl sulfides with trifluoromethyltriisopropylsilane (TIPSCF_3_)^11^ and the radical trifluoromethylthiolation of arenediazonium salts with Me_4_NSCF_3_.^12^ In recent years, a handful of examples were reported where photochemistry was applied for the synthesis of ArSCF_3_ thanks to the easy photogeneration of aryl radicals from aryl diazonium salts and ensuing reaction with a *S*-trifluoromethyl arylsulfonothioate^13a,13b^ or 1,2-bis(trifluoromethyl)disulfane (CF_3_S)_2_ (Scheme 1d).^13c^ Unfortunately, the latter approach requires the presence of a photocatalyst (PC) and a sensitive aryl radical precursor complicated by the special caution required on the use and storage of arenediazonium salts.^14^

**Scheme 1:**
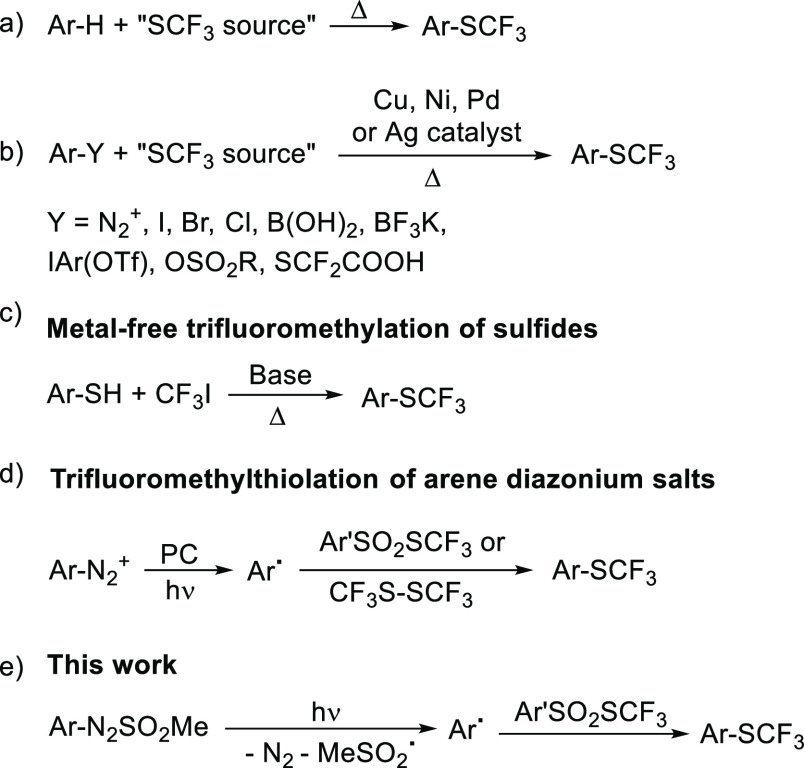


We then envisioned that a straightforward route to Ar–SCF_3_ could involve again aryl radicals but was generated by visible-light irradiation of arylazo sulfones (ArN_2_SO_2_Me, Scheme 1e). Such derivatives exhibit a wavelength selective behavior^15^ and release an aryl radical upon visible-light irradiation^16^ by cleavage of the N–S bond followed by nitrogen loss of the resulting aryl diazenyl radical.^17^ The thus generated aryl radicals were recently applied for the forging of various Ar–C and Ar–heteroatom bonds.^18^ In view of these premises, we planned to use such sulfones for the easy arylation of a SCF_3_-containing derivative to form the desired Ar–SCF_3_. As the trifluoromethylthiolating agent, we exclude the use of (CF_3_S)_2_ due to its difficult handling,^13c^ focusing our attention on *S*-trifluoromethyl arylsulfonothioates.

Preliminary experiments were carried out on compound **1a**, and the obtained results are summarized in Table 1. Irradiation of **1a** (0.125 M, in 1,2-dichloroethane, DCE) in the presence of two equivalents of *S*-(trifluoromethyl) 4-difluorobenzenesulfonothioate (**2a**) by means of a 21 W green LED afforded the arylated compound **3a** in low yields (21%, entry 1). Moving to blue light (entries 2 and 3) resulted in an improvement of the efficiency of the process, and a 53% yield was reached with a 21W blue LED lamp. The use of a different arylazo sulfone (**1a**′, entry 4) as well as the adoption of either electron-rich (**2b**) or difluorinated (**2c**) benzenesulfonothioates (entries 5 and 6) did not afford better results, while, among the different media tested (entries 7–9), DCE furnished the most satisfactory performance. Hydrodeaminated biphenyl can be competitively formed as the main product by changing the reaction medium (e.g., MeCN). Gratifyingly, when doubling the concentration of the reactants, **3a** was isolated in 63% yield (entry 10), and this yield slightly decreased by using a 0.5 M amount of **1a** (entry 11). Notably, increasing the loading of the trifluoromethylthiolating agent **2a** from 2.0 equiv to 4.0 equiv improved the yield up to 75% (entry 12). The formation of **3a** was completely inhibited in the absence of light (entry 13). Dedicated on–off experiments confirmed that the reaction did not proceed in the dark (see Figure S1).

**Table 1:**
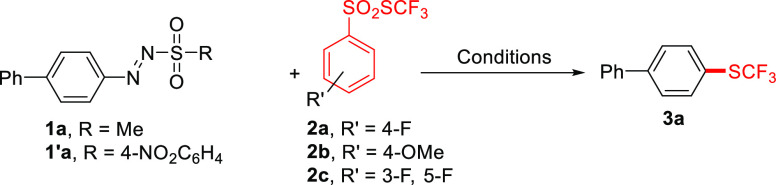
Optimization of the Reaction Conditions^a^

entry	**1a** (conc)	**2** (equiv)	solvent	light source (LED)	yield (%)
1	**1a**, 0.125 M	**2a** (2)	DCE	21 W green	21
2	**1a**, 0.125 M	**2a** (2)	DCE	9 W blue	39
3	**1a**, 0.125 M	**2a** (2)	DCE	21 W blue	53
4	**1′a**, 0.125 M	**2a** (2)	DCE	21 W blue	49
5	**1a**, 0.125 M	**2b** (2)	DCE	21 W blue	49
6	**1a**, 0.125 M	**2c** (2)	DCE	21 W blue	52
7	**1a**, 0.125 M	**2a** (2)	toluene	21 W blue	20
8	**1a**, 0.125 M	**2a** (2)	DMF	21 W blue	19
9	**1a**, 0.125 M	**2a** (2)	MeCN	21 W blue	<5%^b^
10	**1a**, 0.25 M	2a (2)	DCE	21 W blue	63
11	**1a**, 0.5 M	**2a** (2)	DCE	21 W blue	60
12	1a, 0.25 M	2a (4)	DCE	21 W blue	75
13^c^	**1a**, 0.25 M	**2a** (2)	DCE	21 W blue	trace

a Reaction time = 12–36 h.

b Biphenyl was observed as the main product;

c In the dark.

The conditions described in entry 12 have been thus adopted to investigate the scope of the synthetic protocol.

Compound **3a** was also synthesized on a gram scale and isolated in 45% yield (see Table 2). We then investigated the scope of the protocol, as shown in Table 2. The desired trifluoromethylthiolated products have been isolated in discrete to satisfactory yields, and the process showed a good tolerance to both electron-donating and electron-withdrawing substituents present on the aromatic ring, including (thio)alkoxy groups (see products **3c**–**e**, **3p**), halogens (**3i**,**n**), and carbonyls (**3k**–**m**). In some cases, good results were obtained in the presence of only 2 equiv of **2a** (as in the synthesis of **3f** and **3l**). The process was found to be also suitable to prepare naphthyl derivative **3q**, whereas heteroaryl trifluoromethyl thioethers (**3r**–**x**) were mainly obtained in a lower yield. In the latter cases, the hydrodeamination product (see the case of **3u** and **3x**) was detected as the main byproduct (up to 30% yield).

**Table 2:**
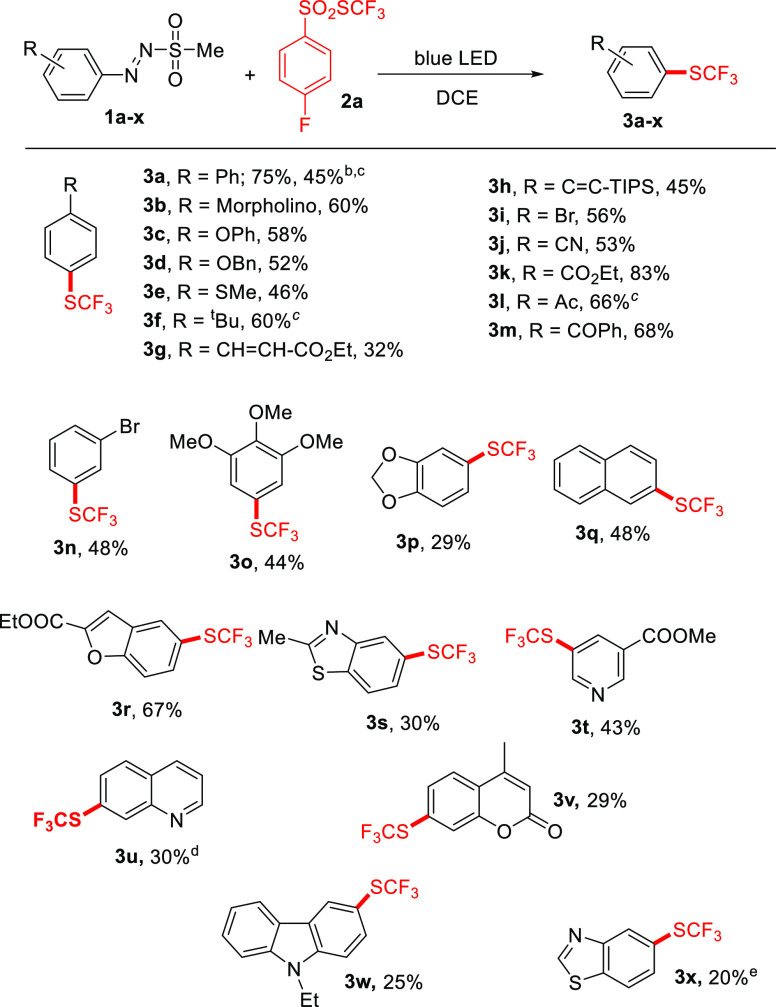
Trifluoromethylthiolation of Arylazo Sulfones^a^

a A solution of **1a**–**x** in DCE in the presence of **2a** (4 equiv) irradiated for 36 h by means of a 21 W Blue Light LED.

b Reaction carried out on 10 mmol **1a**, 48 h irradiation (gram scale = 1.15 g).

c 2 equiv of **2a** employed.

d Quinoline (30% yield) was observed as the byproduct via GC–MS analyses.

e Benzothiazole (24% yield) was observed as the byproduct via GC–MS analyses.

A tentative mechanism is shown in Scheme 2. Sulfones **1a**–**x** possess a weak nπ band between 400 and 500 nm (ε ca. 500 M^–1^) sufficient to allow the absorption in the visible region (465–470 nm blue LEDs have been used as inexpensive light source).^16^ A direct photolysis of **2a** is safely excluded since its absorption at the wavelength used is negligible (see Figure S2). The labile S–N bond underwent a smooth photocleavage (path a) liberating the desired aryl radical Ar^•^ that, as previously described,^13a,13b^ gave a substitution reaction with **2a** (path b) to form derivatives **3a**–**x** along with the stable 4-fluorophenylsulfonyl radical. Hydrogen atom abstraction of Ar^•^ from the solvent to form the hydrodeaminated product Ar–H (path c) is the main competitive path.^18c^ The intermediacy of an aryl radical has been further confirmed by the formation of adduct **4k** that was isolated in 30% yield when the reaction of **1k** with **2a** was carried out in the presence of TEMPO (4 equiv, path d).

**Scheme 2:**
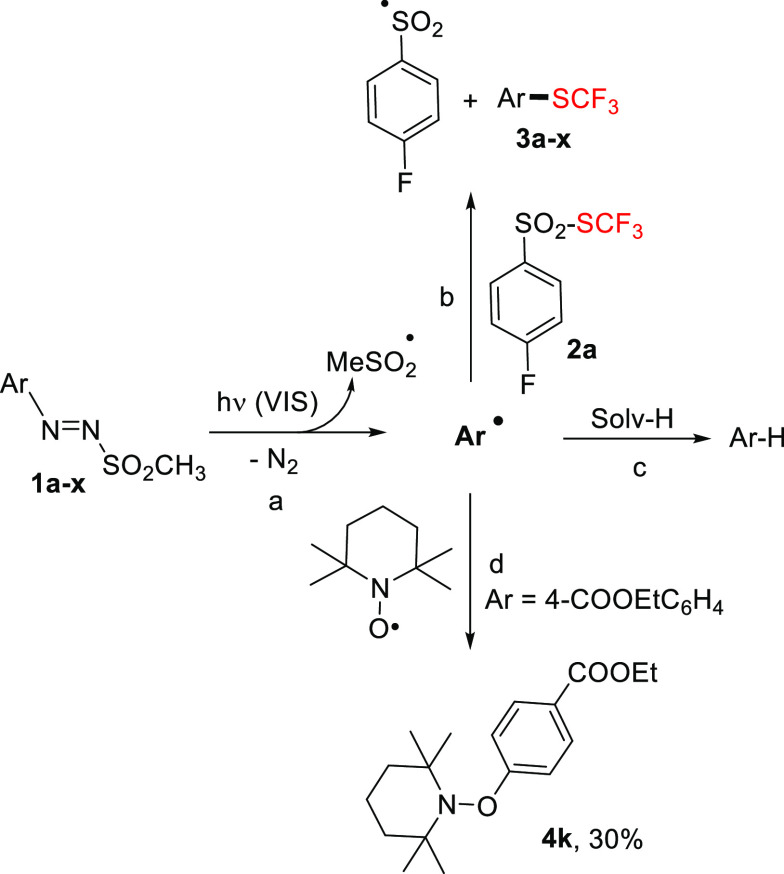
Proposed Mechanism for the Thiotrifluoromethylation of Arylazo Sulfones

In summary, we proposed a radical trifluoromethylthiolation reaction for the formation of aryl–SCF_3_ bonds via simple visible-light activation of bench-stable arylazo sulfones under both metal-free and (photo)catalyst-free conditions. The process employed *S*-(trifluoromethyl) 4-difluorobenzenesulfonothioate as the trifluoromethylthiolating agent and was found suitable for the preparation of both electron-rich and electron-poor SCF_3_-substituted aromatics in discrete to satisfactory yields. In analogy with other metal-free^12^ or photocatalyzed^13a−13c^ trifluoromethylthiolation protocols, a low efficiency in the synthesis of heteroaryl trifluoromethyl thioethers was observed, with the only exception of benzofuran **3r**.

## Experimental Section

### General Remarks

All solvents were distilled prior to use. For chromatography, 200–300 mesh silica gel (Qingdao, China) was employed. ^1^H, ^13^C, and ^19^F NMR spectra were recorded at 400, 100 and 375 MHz with a Bruker ARX 400 spectrometer. Chemical shifts are reported in ppm using tetramethylsilane as an internal standard. HRMS was performed on an Thermo Scientific LTQ Orbitrap XL (ion trap) or Bruker Solarix XR FTMS (Q-TOF) mass instrument. All arylazo sulfones were prepared from the corresponding arenediazonium tetrafluoroborates according to literature procedures.^18e−18g^ The light-promoted reactions were carried out by using a standard blue LED lamp with 20 one-light-emitting diodes (12–28 V, 21 W, 465–470 nm). The distance from the light source to the irradiation vessel was 3 cm.

### Typical Procedure for the Preparation of Arenediazonium Tetrafluoroborates

#### Method A

In a 100 mL round-bottom flask, the aromatic amine (20 mmol) was dissolved in a mixture of H_2_O (10 mL) and HBF_4_ (48% aq, 6 mL). After the mixture was stirred for 15 min, a solution of NaNO_2_ (1.5 g, 1.1 equiv, in 4 mL of H_2_O) was added dropwise at 0 °C (in an ice bath). The mixture was stirred for another 30 min at 0 °C. Then, the arenediazonium tetrafluoroborate was removed by filtration and washed with diethyl ether (2 × 10 mL). The crude product was dried in vacuo for 20 min and was then directly used without further purification.

#### Method B

In a 100 mL round-bottom flask, the aromatic amine (20 mmol) was dissolved in a mixture of ethanol (8 mL) and HBF_4_ (48% aq, 6 mL). Subsequently, *tert*-butyl nitrite (4.7 mL, 2.0 equiv) was added dropwise to the solution at 0 °C. The reaction mixture was stirred for 30 min at 0 °C, and anhydrous diethyl ether (20 mL) was added to precipitate the corresponding arenediazonium tetrafluoroborate. The solid was filtered off and washed with diethyl ether (2 × 10 mL). The product was then dried in vacuo for 20 min and used without further purification.

#### Typical Procedure for the Preparation for Arylazo Sulfones

In a 100 mL round-bottom flask, the suspension of the freshly prepared arenediazonium tetrafluoroborate (20 mmol) in CH_2_Cl_2_ (20 mL) was stirred at 0 °C; then sodium methanesulfinate (2.24 g, 1.1 equiv) was added to the reaction system in one portion. The temperature was risen to rt; the mixture was stirred overnight and then filtered, and the obtained solution evaporated. The crude product was thus purified by dissolution in CH_2_Cl_2_ and precipitation by adding cold petroleum ether. The obtained arylazo sulfones were filtered and dried in vacuo for 30 min.

### Characterization Data for the Arylazo Sulfones **1a**–**t**

#### 1-([1,1′-Biphenyl]-4-yl)-2-(methylsulfonyl)diazene (**1a**).^18d^

Yellow solid (3.6 g, 69%). Mp (dec): 107–109 °C. ^1^H NMR (400 MHz, CDCl_3_): δ 8.03 (d, *J* = 8.6 Hz, 2H), 7.80 (d, *J* = 8.6 Hz, 2H), 7.68–7.66 (m, 2H), 7.52–7.48 (m, 2H), 7.46–7.42 (m, 1H), 3.24 (s, 3H).

#### 1-([1,1′-Biphenyl]-4-yl)-2-((4-nitrophenyl)sulfonyl)diazene (**1a**′)

Yellow solid (4.0 g, 54%). Mp (dec): 100–101 °C. ^1^H NMR (400 MHz, CDCl_3_): δ 8.45 (d, *J* = 8.88 Hz, 2H), 8.19 (d, *J* = 8.92 Hz, 2H), 7.89 (d, *J* = 8.64 Hz, 2H), 7.75 (d, *J* = 8.68 Hz, 2H), 7.64–7.62 (m, 2H), 7.51–7.41 (m, 3H). ^13^C {^1^H} NMR (100 MHz, CDCl_3_): δ 151.3, 148.7, 147.9, 139.4, 138.9, 131.8, 129.1, 129.0, 128.2, 127.3, 125.5, 124.1. IR (neat, υ cm^–1^): 3413, 1616, 1526, 1352, 1149, 1074, 952, 858, 620, 517. The compound was found to decompose upon HRMS analysis.

#### 4-(4-((Methylsulfonyl)diazenyl)phenyl)morpholine (**1b**)

Orange solid (2.1 g, 39%). Mp (dec): 139–140 °C. ^1^H NMR (400 MHz, CDCl_3_): δ 7.86 (d, *J* = 9.2 Hz, 2H), 6.90 (d, *J* = 9.2 Hz, 2H), 3.86 (t, *J* = 4.8 Hz, 4H), 3.46 (t, *J* = 5.1 Hz, 4H), 3.14 (s, 3H). ^13^C{^1^H} NMR (100 MHz, CDCl_3_): δ 155.7, 140.9, 127.8, 113.1, 66.3, 46.8, 35.0. IR (neat, υ cm^–1^): 3413, 1607, 1379, 1318, 1234, 1141, 1067, 619, 533. HRMS (ESI) *m*/*z*: [M + H]^+^ calcd for C_11_H_16_N_3_O_3_S, 270.0907; found, 270.0904.

#### 1-(Methylsulfonyl)-2-(4-phenoxyphenyl)diazene (**1c**)

Brown solid (4.4 g, 80%). Mp (dec): 110–111 °C. ^1^H NMR (400 MHz, CDCl_3_): δ 7.94 (d, *J* = 9.0 Hz, 2H), 7.47–7.43 (m, 2H), 7.27 (t, *J* = 7.4 Hz, 1H), 7.13–7.01 (m, 4H), 3.20 (s, 3H). ^13^C{^1^H} NMR (100 MHz, CDCl_3_): δ 164.3, 154.5, 144.0, 130.2, 127.1, 125.4, 120.6, 117.7, 34.8. IR (neat, υ cm^–1^): 3413, 1615, 1486, 1338, 1243, 1137, 949, 853, 773, 617, 502. HRMS (ESI) *m*/*z*: [M + H]^+^ calcd for C_13_H_13_N_2_O_3_S, 277.0641; found, 277.0641.

#### (Benzyloxy)phenyl)-2-(methylsulfonyl)diazene (**1d**)

Pale yellow solid (3.0 g, 52%). Mp (dec): 150–151 °C. ^1^H NMR (400 MHz, CDCl_3_): δ 7.95 (d, *J* = 9.8 Hz, 2H), 7.45–7.36 (m, 5H), 7.11 (d, *J* = 9.1 Hz, 2H), 5.19 (s, 2H), 3.19 (s, 3H). ^13^C{^1^H} NMR (100 MHz, CDCl_3_): δ 164.8, 143.3, 135.5, 128.8, 128.5, 127.5, 127.3, 115.7, 70.7, 34.9. IR (neat, υ cm^–1^): 3413, 1614, 1485, 1338, 1137, 949, 852, 617, 502. HRMS (ESI) *m*/*z*: [M + H]^+^calcd for C_14_H_15_N_2_O_3_S, 291.0795; found, 291.0798.

#### 1-(Methylsulfonyl)-2-(4-(methylthio)phenyl)diazene (**1e**)

Yellow solid (2.9 g, 63%). Mp (dec): 130–132 °C. ^1^H NMR (400 MHz, CDCl_3_): δ 7.87 (d, *J* = 8.8 Hz, 2H), 7.35 (d, *J* = 8.0 Hz, 2H) 3.20 (s, 3H), 2.58 (s, 3H). ^13^C{^1^H} NMR (100 MHz, CDCl_3_): δ 150.0, 145.8, 125.4, 124.9, 34.8, 14.7. IR (neat, υ cm^–1^): 3413, 1614, 1486, 1337, 1137, 949, 617, 502. HRMS (ESI) *m*/*z*: [M + H]^+^ calcd for C_8_H_11_N_2_O_2_S_2_, 231.0257; found, 231.0256.

#### 1-(4-(*tert*-Butyl)phenyl)-2-(methylsulfonyl)diazene (**1f**).^18d^

Yellow solid (2.6 g, 55%). Mp (dec): 78–80 °C. ^1^H NMR (400 MHz, CDCl_3_): δ 7.88 (d, *J* = 8.8 Hz, 2H), 7.59 (d, *J* = 8.8 Hz, 2H), 3.20 (s, 3H), 1.36 (s, 9H).

#### Ethyl 3-(4-((*E*)-(Methylsulfonyl)diazenyl)phenyl)acrylate (**1g**).^18e^

Yellow solid (5.5 g, 98%). Mp (dec): 98–100 °C. ^1^H NMR (400 MHz, CDCl_3_): δ 7.95(d, *J* = 8.5 Hz, 2H), 7.68–7.72 (m, 3H), 6.57 (d, *J* = 16.1 Hz, 1H), 4.28 (q, *J* = 7.1 Hz, 2H), 3.22 (s, 3H), 1.34 (t, *J* = 7.1 Hz, 3H).

#### 1-(Methylsulfonyl)-2-(4-((triisopropylsilyl)ethynyl)phenyl)diazene (**1h**).^18e^

Yellow solid (2.7 g, 37%). Mp (dec): 63–64 °C. ^1^H NMR (400 MHz, CDCl_3_): δ 7.89 (d, *J* = 8.5 Hz, 2H), 7.65 (d, *J* = 8.5 Hz, 2H), 3.22 (s, 3H), 1.16–1.12 (m, 21H).

#### 1-(4-Bromophenyl)-2-(methylsulfonyl)diazene (**1i**).^18f^

Yellow solid (3.2 g, 60%). Mp (dec): 131–133 °C. ^1^H NMR (400 MHz, CDCl_3_): δ 7.83 (d, *J* = 8.8 Hz, 2H), 7.74 (d, *J* = 8.8 Hz, 2H), 3.22 (s, 3H).

#### 4-((Methylsulfonyl)diazenyl)benzonitrile (**1j**).^18h^

Yellow solid (3.3 g, 79%). Mp (dec): 114–115 °C. ^1^H NMR (400 MHz, CDCl_3_): δ 8.04 (d, *J* = 8.4 Hz, 2H), 7.89 (d, *J* = 8.4 Hz, 2H), 3.26 (s, 3H).

#### Ethyl 4-((Methylsulfonyl)diazenyl)benzoate (**1k**).^18d^

Yellow solid (3.7 g, 73%). Mp (dec): 83–84 °C. ^1^H NMR (400 MHz, CDCl_3_): δ 8.25(d, *J* = 8.6 Hz, 2H), 7.99 (d, *J* = 8.6 Hz, 2H), 4.44 (q, *J* = 7.1 Hz, 2H), 3.25 (s, 3H), 1.43 (t, *J* = 7.1 Hz, 3H).

#### 1-(4-((Methylsulfonyl)diazenyl)phenyl)ethanone (**1l**).^18d^

Yellow solid (3.6 g, 80%). Mp (dec): 119–120 °C. ^1^H NMR (400 MHz, CDCl_3_): δ 8.13 (d, *J* = 8.5 Hz, 2H), 8.00 (d, *J* = 8.5 Hz, 2H), 3.24 (s, 3H), 2.67 (s, 3H).

#### (4-((Methylsulfonyl)diazenyl)phenyl)(phenyl)methanone (**1m**)

Yellow solid (4.3 g, 75%). Mp (dec): 130 °C. ^1^H NMR (400 MHz, CDCl_3_): δ 8.01 (dd, *J* = 8.6, 31.4 Hz, 4H), 7.81 (d, *J* = 7.04 Hz, 2H), 7.67–7.62 (m, 1H), 7.52 (t, *J* = 7.88 Hz, 2H), 3.27 (s, 3H). ^13^C{^1^H} NMR (100 MHz, CDCl_3_): δ 195.1, 150.6, 142.8, 136.4, 133.2, 131.0, 130.0, 128.5, 124.2, 34.8. IR (neat, υ cm^–1^): 3413, 1659, 1617, 1340, 1276, 1159, 1069, 954, 861, 700. HRMS (ESI) *m*/*z*: [M + H]^+^ calcd for C_14_H_13_N_2_O_3_S, 289.0638; found, 289.0641.

#### 1-(3-Bromophenyl)-2-(methylsulfonyl)diazene (**1n**).^18e^

Yellow solid (2.5 g, 47%). Mp (dec): 95–96 °C. ^1^H NMR (400 MHz, CDCl_3_): δ 8.06 (s, 1H), 7.90 (d, *J* = 8.0 Hz, 1H), 7.77 (d, *J* = 8.0 Hz, 1H), 7.47 (t, *J* = 8.0 Hz, 1H), 3.22 (s, 3H).

#### 1-(Methylsulfonyl)-2-(3,4,5-trimethoxyphenyl)diazene (**1o**)

Pale yellow solid (2.7 g, 49%). Mp (dec): 122–124 °C. ^1^H NMR (400 MHz, CDCl_3_): δ 7.26 (s, 2H), 4.00 (s, 3H), 3.94 (s, 6H), 3.22 (s, 3H). ^13^C{^1^H} NMR (100 MHz, CDCl_3_): δ 153.5, 144.4, 102.5, 61.1 (d, *J* = 6.4 Hz), 56.3 (d, *J* = 5.4 Hz), 34.9. IR (neat, υ cm^–1^): 3413, 1614, 1486, 1338, 1137, 949, 617, 502. HRMS (ESI) *m*/*z*: [M + H]^+^calcd for C_10_H_14_N_2_O_5_S, 275.0696; found, 275.0696.

#### 1-(Benzo[*d*][1,3]dioxol-5-yl)-2-(methylsulfonyl)diazene (**1p**)

Dark green solid (0.91 g, 20%). Mp (dec): 113–114 °C. ^1^H NMR (400 MHz, CDCl_3_): δ 7.69 (dd, *J* = 2.0, 8.2 Hz, 1H), 7.34 (d, *J* = 1.9, Hz, 1H), 6.99 (d, *J* = 8.2 Hz, 1H), 6.14 (s, 2H), 3.18 (s, 3H). ^13^C{^1^H} NMR (100 MHz, CDCl_3_): δ 154.4, 149.5, 144.8, 129.3, 108.4, 102.8, 98.8, 34.9. IR (neat, υ cm^–1^): 3414, 1617, 1477, 1414, 1152, 618. HRMS (EI) *m*/*z*: [M + H]^+^ calcd for C_8_H_9_N_2_O_4_S, 229.0278; found, 229.0281.

#### 1-(Methylsulfonyl)-2-(naphthalen-2-yl)diazene (**1q**).^18e^

Yellow solid (2.2 g, 47%). Mp (dec): 108–109 °C. ^1^H NMR (400 MHz, CDCl_3_): δ 8.56 (s, 1H), 8.03–8.01 (m, 1H), 7.92–7.89 (m, 3H), 7.67 (t, *J* = 7.5 Hz, 1H), 7.61 (t, *J* = 7.5 Hz, 1H), 3.27 (s, 3H).

#### Ethyl 5-((Methylsulfonyl)diazenyl)benzofuran-2-carboxylate (**1r**).^18e^

Yellow solid (4.6 g, 78%). Mp (dec): 117–118 °C. ^1^H NMR (400 MHz, CDCl_3_): δ 8.35 (d, *J* = 2.0 Hz, 1H), 8.05 (dd, *J* = 2.0, 9.0 Hz, 1H), 7.73 (d, *J* = 9.0 Hz, 1H), 7.64 (d, *J* = 0.8 Hz, 1H), 4.47 (q, *J* = 7.1 Hz, 2H), 3.24 (s, 3H), 1.44 (t, *J* = 7.1 Hz, 3H).

#### 2-Methyl-5-((methylsulfonyl)diazenyl)benzo[*d*]thiazole (**1s**).^18e^

Brown solid (3.9 g, 77%). Mp (dec): 123–124 °C. ^1^H NMR (400 MHz, CDCl_3_): δ 8.50 (d, *J* = 1.9 Hz, 1H), 7.95 (d, *J* = 8.6 Hz, 1H), 7.92 (dd, *J* = 1.9, 8.6 Hz, 1H), 3.25 (s, 3H), 2.89 (s, 3H).

#### Methyl 5-((Methylsulfonyl)diazenyl)nicotinate (**1t**).^18e^

Yellow solid (4.1 g, 85%). Mp (dec): 103–105 °C. ^1^H NMR (400 MHz, CDCl_3_): δ 9.46 (d, *J* = 1.9 Hz, 1H), 9.38 (d, *J* = 2.3 Hz, 1H), 8.73 (t, *J* = 2.1 Hz, 1H), 4.02 (s, 3H), 3.29 (s, 3H).

#### 7-((Methylsulfonyl)diazenyl)quinoline (**1u**)

Red solid (3.1 g, 66%). Mp (dec): 140–142 °C. ^1^H NMR (400 MHz, CDCl_3_): δ 9.06 (dd, *J* = 1.5, 4.2 Hz, 1H), 8.79 (s, 1H), 8.25 (d, *J* = 8.2 Hz, 1H), 7.99–7.92 (m, 2H), 7.59–7.56 (m, 1H), 3.31 (s, 3H). ^13^C{^1^H} NMR (100 MHz, CDCl_3_): δ 152.1, 149.0, 148.1, 136.0, 133.7, 132.0, 129.8, 123.8, 116.2, 34.8. IR (neat, υ cm^–1^): 3440, 3045, 3020, 1500, 1463, 1328, 1151, 946, 867, 772, 746, 616, 566, 443. HRMS (ESI) *m*/*z*: [M + Na]^+^ calcd for C_10_H_9_N_3_O_2_SNa, 258.0308; found, 258.0308.

#### 4-Methyl-7-((methylsulfonyl)diazenyl)-2*H*-chromen-2-one (**1v**)

Orange solid (2.3 g, 43%). Mp (dec): 102–104 °C. ^1^H NMR (400 MHz, CDCl_3_): δ 7.88–7.85 (m, 2H), 7.80 (d, *J* = 9.0 Hz, 1H), 6.46 (d, *J* = 1.3 Hz, 1H), 3.27 (s, 3H), 2.51 (d, *J* = 1.3 Hz, 3H). ^13^C{^1^H} NMR (100 MHz, CDCl_3_): δ 159.04, 153.9, 151.1, 150.2, 126.0, 125.0, 118.9, 118.0, 113.6, 35.0, 18.7. IR (neat, υ cm^–1^): 3440, 3045, 3006, 2920, 1715, 1624, 14110, 1340, 1259, 1158, 979, 903, 558. HRMS (ESI) *m*/*z*: [M + H]^+^ calcd for C_11_H_11_N_2_O_4_S, 267.0434; found, 267.0434.

#### 9-Ethyl-3-((methylsulfonyl)diazenyl)-9*H*-carbazole (**1w**)

Brown solid (4.9 g, 81%). Mp (dec): 145–147 °C. ^1^H NMR (400 MHz, CDCl_3_): δ 8.70 (d, *J* = 1.9 Hz, 1H), 8.12 (d, *J* = 7.8 Hz, 1H), 8.08 (dd, *J* = 1.9, 8.9 Hz, 1H), 7.58–7.54 (m, 1H), 7.48–7.45 (m, 2H), 7.37–7.33 (m, 1H), 4.41 (q, *J* = 7.2 Hz, 2H), 3.24 (s, 3H), 1.49 (t, *J* = 7.3 Hz, 3H). ^13^C{^1^H} NMR (100 MHz, CDCl_3_): δ 144.3, 142.3, 140.9, 127.3, 123.7, 123.3, 122.2, 121.0, 120.5, 111.4, 109.6, 109.2, 38.1, 35.0, 13.8. IR (neat, υ cm^–1^): 3442, 3032, 2975, 1593, 1498, 1418, 1119, 958, 835, 489. HRMS (ESI) *m*/*z*: [M + H]^+^ calcd for C_15_H_16_N_3_O_2_S, 302.0958; found, 302.0958.

#### 5-((Methylsulfonyl)diazenyl)benzo[*d*]thiazole (**1x**)

Yellow solid (1.5 g, 31%). Mp (dec): 152–154 °C. ^1^H NMR (400 MHz, CDCl_3_): δ 9.16 (s, 1H), 8.74 (d, *J* = 1.8 Hz, 1H), 8.12 (d, *J* = 8.1 Hz, 1H), 8.02 (dd, *J* = 1.9, 8.7 Hz, 1H), 3.28 (s, 3H). ^13^C{^1^H} NMR (100 MHz, CDCl_3_): δ 156.6, 153.8, 147.7, 140.7, 123.4, 122.9, 118.2, 34.9. IR (neat, υ cm^–1^): 3438, 3049, 1477, 1326, 1151, 865, 823, 531. HRMS (ESI) *m*/*z*: [M + H]^+^ calcd for C_8_H_8_N_3_O_2_S_2_, 242.0052; found, 242.0052.

#### Synthesis of Compound **2c**

Compound **2c** was obtained by following a procedure previously reported for the synthesis of compounds **2a**,**b**.^19^ A mixture of 3,5-difluorobenzenesulfinate sodium salt (20 mmol, 1 equiv), *N*-[(trifuoromethyl)thio] aniline (1 equiv),^20^ and paratoluenesulfonic acid (2.5 equiv) in DCE (130 mL) was stirred at room temperature. After the completion of the reaction, indicated by TLC, the mixture was filtered with a sand core funnel with silica gel, washed with CH_2_Cl_2_, and dried over Na_2_SO_4_. After concentration, the residue was purified by flash column chromatography to obtain the final product **2c** (light yellow oil, 3.8 g, 68%). ^1^H NMR (400 MHz, CDCl_3_): δ 7.55 (dd, *J* = 1.92, 5.68 Hz, 2H), 7.19 (tt, *J* = 2.28, 8.20 Hz, 1H). ^13^C{^1^H} NMR (100 MHz, CDCl_3_): δ 164.1 (d, *J* = 11.52 Hz, 1C), 161.5 (d, *J* = 11.36 Hz, 1C), 147.0 (t, *J* = 9.46 Hz, 1C), 120.7 (q, *J* = 311.66 Hz, 1C), 111.3 (d, *J* = 29.06 Hz, 1C), 111.3 (d, *J* = 11.31 Hz, 1C), 110.9 (t, *J* = 24.89 Hz, 1C). ^19^F NMR (375 MHz, CDCl_3_): δ −38.1 (s, 3F), −103.4 (s, 2F). The present compound was found to decompose upon HRMS analysis.

#### Synthesis of Thiotrifluoromethyl Arenes (**3a**–**x**)

A solution of the chosen arylazo sulfone **(1a**–**x**, 0.25 M) in DCE was placed in a 10 mL microwave tube, and then *S*-(trifluoromethyl) 4-fluorobenzenesulfonothioate (**2a**, 1 mmol, 2.0–4.0 equiv, see Table 1) was added. The resulting solution was irradiated under stirring at room temperature for 36 h by means of a 21 W blue LED, and then the reaction mixture was concentrated under reduced pressure to evaporate the solvent. The residue was purified by silica gel column chromatography (petroleum ether/ethyl acetate mixture as the eluent).

#### Gram-Scale Preparation of **3a**

1-([1,1′-Biphenyl]-4-yl)-2- (methylsulfonyl) diazene (**1a**, 10 mmol, 2.6 g) was placed in a round-bottom flask and dissolved in DCE (100 mL) under aerated conditions. Compound **2a** (20 mmol, 2.0 equiv) was added, and the resulting solution was irradiated under stirring for 48 h by means of a 21 W blue LED. The reaction mixture was concentrated under reduced pressure to evaporate the solvent, and the residue was purified by silica gel column chromatography (petroleum ether as the eluent) to obtain [1,1′-biphenyl]-4-yl (trifluoromethyl) sulfane **3a** (1.15 g, 45%) as a white solid.

### Characterization Data for the Isolated Products (**3a**–**x**)

#### [1,1′-Biphenyl]-4-yl(trifluoromethyl)sulfane (**3a**).^13^

After purification by silica gel column chromatography (PE), compound **3a** was isolated as a white solid (50 mg, 75%). *R*_*f*_ (PE) = 0.8. ^1^H NMR (400 MHz, CDCl_3_): δ 7.74 (d, *J* = 8.3 Hz, 2H), 7.66–7.60 (m, 4H), 7.49 (t, *J* = 7.4 Hz, 2H), 7.42 (d, *J* = 7.4 Hz, 1H). ^13^C{^1^H} NMR (100 MHz, CDCl_3_): δ 143.8, 139.7, 136.7, 129.6 (q, ^1^*J*(C,F) = 306.4 Hz), 128.9, 128.1, 127.2, 123.1 (q, ^2^*J*(C,F) = 1.9 Hz). ^19^F NMR (375 MHz, CDCl_3_): δ −42.7 (s, 3F).

#### 4-(4-((Trifluoromethyl)thio)phenyl)morpholine (**3b**).^9^

After purification by silica gel column chromatography (PE/EA = 30:1), compound **3b** was isolated as a white solid (39 mg, 60%). *R*_*f*_ (PE/EA = 20:1) = 0.3. ^1^H NMR (400 MHz, CDCl_3_): δ 7.52 (d, *J* = 8.3 Hz, 2H), 6.88 (d, *J* = 8.3 Hz, 2H), 3.85 (t, *J* = 5.1 Hz, 4H), 3.23 (t, *J* = 5.0 Hz, 4H). ^13^C{^1^H} NMR (100 MHz, CDCl_3_): δ 152.9, 137.9, 129.7 (q, ^1^*J*(C,F) = 306.3 Hz), 115.2, 112.5, 66.6, 48.0. ^19^F NMR (375 MHz, CDCl_3_): δ −44.1 (s, 3F).

#### (4-Phenoxyphenyl)(trifluoromethyl)sulfane (**3c**).^13^

After purification by silica gel column chromatography (PE), compound **3c** was isolated as a white solid (39 mg, 58%). *R*_*f*_ (PE) = 0.55. ^1^H NMR (400 MHz, CDCl_3_): δ 7.60 (d, *J* = 8.7 Hz, 2 H), 7.40 (t, *J* = 7.6 Hz, 2H), 7.22 (t, *J* = 7.4 Hz, 1H), 7.07 (d, *J* = 7.7 Hz, 2H), 7.00 (d, *J* = 8.8 Hz, 2H). ^13^C{^1^H} NMR (100 MHz, CDCl_3_): δ 160.4, 155.6, 138.3, 129.6 (q, ^1^*J*(C,F) = 306.3 Hz), 130.0, 124.5, 120.1, 118.6, 117.3 (q, ^2^*J*(C,F) = 2.0 Hz). ^19^F NMR (375 MHz, CDCl_3_): δ −43.5 (s, 3F).

#### (4-(Benzyloxy)phenyl)(trifluoromethyl)sulfane (**3d**).^21^

After purification by silica gel column chromatography (PE), compound **3d** was isolated as a white solid (37 mg, 52%). *R*_*f*_ (PE) = 0.6. ^1^H NMR (400 MHz, CDCl_3_): δ 7.59 (d, *J* = 8.8 Hz, 2H), 7.45–7.34 (m, 5H), 7.01 (d, *J* = 8.9 Hz, 2H), 5.09 (s, 2H). ^13^C{^1^H} NMR (100 MHz, CDCl_3_): δ 161.0, 138.3, 136.2, 129.6 (q, ^1^*J*(C,F) = 306.2 Hz), 128.7, 128.2, 127.5, 115.8, 115.2 (q, ^2^*J*(C,F) = 2.0 Hz), 70.2. ^19^F NMR (375 MHz, CDCl_3_): δ −43.8 (s, 3F).

#### Methyl(4-((trifluoromethyl)thio)phenyl)sulfane (**3e**).^13^

After purification by silica gel column chromatography (PE), compound **3e** was isolated as a yellow oil (26 mg, 46%). *R*_*f*_ (PE) = 0.6. ^1^H NMR (400 MHz, CDCl_3_): δ 7.55 (d, *J* = 8.4 Hz, 2H), 7.25 (d, *J* = 8.8 Hz, 2H), 2.50 (s, 3H). ^13^C{^1^H} NMR (100 MHz, CDCl_3_): δ 143.3, 136.6, 129.5 (q, ^1^*J*(C,F) = 306.4 Hz), 126.4, 119.7, 15.0. ^19^F NMR (375 MHz, CDCl_3_): δ −43.3 (s, 3F).

#### (4-(*tert*-Butyl)phenyl)(trifluoromethyl)sulfane (**3f**).^13^

After purification by silica gel column chromatography (PE/EA = 100:1), compound **3f** was isolated as a colorless oil (35 mg, 60%). *R*_*f*_ (PE/EA = 50:1) = 0.6. ^1^H NMR (400 MHz, CDCl_3_): δ 7.58 (d, *J* = 8.0 Hz, 2H), 7.43 (d, *J* = 8.4 Hz, 2H), 1.33 (s, 9H). ^13^C{^1^H} NMR (100 MHz, CDCl_3_): δ 154.4, 136.1, 129.7 (q, ^1^*J*(C,F) = 306.0 Hz), 126.6, 120.9 (q, ^2^*J*(C,F) = 1.8 Hz), 34.9, 31.1. ^19^F NMR (375 MHz, CDCl_3_): δ −43.0 (s, 3F).

#### Ethyl (*E*)-3-(4-((Trifluoromethyl)thio)phenyl)acrylate (**3g**)

After purification by silica gel column chromatography (PE/EA = 50:1), compound **3g** was isolated as a white solid (22 mg, 32%). *R*_*f*_ (PE/EA = 40:1) = 0.4. ^1^H NMR (400 MHz, CDCl_3_): δ 7.68–7.65 (m, 3H), 7.55 (d, *J* = 8.3 Hz, 2H), 6.49 (d, *J* = 16.0 Hz, 1H), 4.28 (q, *J* = 7.2 Hz, 2H), 1.34 (t, *J* = 7.1 Hz, 3H). ^13^C{^1^H} NMR (100 MHz, CDCl_3_): δ 166.4, 142.7, 136.9, 136.5, 129.4 (q, ^1^*J*(C,F) = 306.6 Hz), 128.7, 126.2 (q, ^2^*J*(C,F) = 2.0 Hz), 120.6, 60.8, 14.3. ^19^F NMR (375 MHz, CDCl_3_): δ −42.3 (s, 3F). HRMS (EM) *m*/*z*: [M – e]^+^calcd for C_12_H_11_F_3_O_2_S, 276.0432; found, 276.0417.

#### Triisopropyl((4-((trifluoromethyl)thio)phenyl)ethynyl)silane (**3h**)

After purification by silica gel column chromatography (PE), compound **3h** was isolated as a colorless oil (40 mg, 45%). *R*_*f*_ (PE) = 0.8. ^1^H NMR (400 MHz, CDCl_3_): δ 7.59 (d, *J* = 8.2 Hz, 2H), 7.51 (d, *J* = 8.2 Hz, 2H), 1.13 (s, 21H). ^13^C{^1^H} NMR (100 MHz, CDCl_3_): δ 136.0, 134.0, 132.9, 124.4 (q, ^1^*J*(C,F) = 306.4 Hz), 126.4, 124.2 (q, ^2^*J*(C,F) = 2.1 Hz), 105.5, 94.1, 18.6, 11.3. ^19^F NMR (375 MHz, CDCl_3_): δ −42.6 (s, 3F). HRMS (EM) *m*/*z*: [M – e]^+^calcd for C_18_H_25_F_3_SSi, 358.1398; found, 358.1385.

#### (4-Bromophenyl)(trifluoromethyl)sulfane (**3i**).^13^

After purification by silica gel column chromatography (PE), compound **3i** was isolated as a colorless oil (36 mg, 56%). *R*_*f*_ (PE) = 0.85. ^1^H NMR (400 MHz, CDCl_3_): δ 7.57 (d, *J* = 8.8 Hz, 2H), 7.52 (d, *J* = 8.5 Hz, 2H). ^13^C{^1^H} NMR (100 MHz, CDCl_3_): δ 137.7 132.8 129.2 (q, ^1^*J*(C,F) = 306.6 Hz), 126.0, 123.4 (q, ^2^*J*(C,F) = 2.1 Hz). ^19^F NMR (375 MHz, CDCl_3_): δ −42.7 (s, 3F).

#### 4-((Trifluoromethyl)thio)benzonitrile (**3j**).^13^

After purification by silica gel column chromatography (PE/EA = 100:1), compound **3j** was isolated as a white solid (27 mg, 53%). *R*_*f*_ (PE/EA = 50:1) = 0.35. ^1^H NMR (400 MHz, CDCl_3_): δ 7.77 (d, *J* = 8.4 Hz, 2H), 7.72 (d, *J* = 8.4 Hz, 2H). ^13^C{^1^H} NMR (100 MHz, CDCl_3_): δ 136.0 132.9, 130.6 (q, ^2^*J*(C,F) = 2.1 Hz), 129.0 (q, ^1^*J*(C,F) = 307.0 Hz,), 117.6, 114.7. ^19^F NMR (375 MHz, CDCl_3_): δ −41.5 (s, 3F).

#### Methyl 4-((Trifluoromethyl)thio)benzoate (**3k**).^13^

After purification by silica gel column chromatography (PE/EA = 50:1), compound **3k** was isolated as a yellow oil (52 mg, 83%). *R*_*f*_ (PE/EA = 50:1) = 0.5. ^1^H NMR (400 MHz, CDCl_3_): δ 8.08 (d, *J* = 8.5 Hz, 2H), 7.71 (d, *J* = 8.0 Hz, 2H), 4.40 (q, *J* = 7.1 Hz, 2H), 1.40 (t, *J* = 7.1 Hz, 3H). ^13^C{^1^H} NMR (100 MHz, CDCl_3_): δ 165.5, 135.5, 132.6, 130.8, 129.7 (q, ^2^*J*(C,F) = 2.0 Hz), 129.3 (q, ^1^*J*(C,F) = 306.5 Hz), 61.5, 14.2. ^19^F NMR (375 MHz, CDCl_3_): δ −41.9 (s, 3F).

#### 1-(4-((Trifluoromethyl)thio)phenyl)ethanone (**3l**).^13^

After purification by silica gel column chromatography (PE/EA = 50:1), compound **3l** was isolated as a yellow soil (36 mg, 66%). *R*_*f*_ (PE/EA = 50:1) = 0.25. ^1^H NMR (400 MHz, CDCl_3_): δ 7.98 (d, *J* = 8.4 Hz, 2H), 7.74 (d, *J* = 8.3 Hz, 2H), 2.63 (s, 3H). ^13^C{^1^H} NMR (100 MHz, CDCl_3_): δ 197.0, 138.5, 135.7, 130.0 (q, ^2^*J*(C,F) = 2.0 Hz), 129.3 (q, ^1^*J*(C,F) = 306.6 Hz), 129.1, 26.7. ^19^F NMR (375 MHz, CDCl_3_): δ −41.8 (s, 3F).

#### Phenyl(4-((trifluoromethyl)thio)phenyl)methanone (**3m**).^13^

After purification by silica gel column chromatography (PE/EA = 100:1), compound **3m** was isolated as a white solid (48 mg, 68%). *R*_*f*_ (PE/EA = 50:1) = 0.4. ^1^H NMR (400 MHz, CDCl_3_): δ 7.84–7.76 (m, 6H), 7.63 (t, *J* = 7.4 Hz, 1H), 7.51 (t, *J* = 8.4 Hz, 2H). ^13^C{^1^H} NMR (100 MHz, CDCl_3_): δ 195.5, 139.5, 136.8, 135.5, 133.0, 130.6, 130.7, 129.3 (q, ^1^*J*(C,F) = 306.7 Hz), 129.1 (q, ^2^*J*(C,F) = 2.0 Hz), 128.5. ^19^F NMR (375 MHz, CDCl_3_): δ −41.8 (s, 3F).

#### (3-Bromophenyl)(trifluoromethyl)sulfane (**3n**).^22^

After purification by silica gel column chromatography (PE), compound **3n** was isolated as a white solid (31 mg, 48%): *R*_*f*_ (PE) = 0.8. ^1^H NMR (400 MHz, CDCl_3_): δ 7.82 (s, 1H), 7.64–7.59(m, 2H), 7.31 (t, *J* = 8.0 Hz, 1H). ^13^C{^1^H} NMR (100 MHz, CDCl_3_): δ 138.7, 134.7, 134.0, 130.7, 129.3 (q, ^1^*J*(C,F) = 306.5 Hz), 126.3 (q, ^2^*J*(C,F) = 2.1 Hz), 122.9. ^19^F NMR (375 MHz, CDCl_3_): δ −42.4 (s, 3F).

#### (Trifluoromethyl)(3,4,5-trimethoxyphenyl)sulfane (**3o**).^23^

After purification by silica gel column chromatography (PE/EA = 40:1), compound **3o** was isolated as a yellow solid (30 mg, 44%). *R*_*f*_ (PE/EA = 40:1) = 0.35. ^1^H NMR (400 MHz, CDCl_3_): δ 6.86 (s, 2H), 3.88(s, 9H). ^13^C{^1^H} NMR (100 MHz, CDCl_3_): δ 153.4, 140.6, 129.6 (q, ^1^*J*(C,F) = 306.4 Hz), 118.5 (q, ^2^*J*(C,F) = 2.1 Hz), 113.7, 60.9, 56.3. ^19^F NMR (375 MHz, CDCl_3_): δ −43.0 (s, 3F).

#### 5-((Trifluoromethyl)thio)benzo[*d*][1,3]dioxole (**3p**).^21^

After purification by silica gel column chromatography (PE), compound **3p** was isolated as a white solid (16 mg, 29%). *R*_*f*_ (PE) = 0.6. Mp = 113–114 °C. ^1^H NMR (400 MHz, CDCl_3_): δ 7.17 (dd, *J* = 1.72, 8.08 Hz, 1H), 7.09 (d, *J* = 1.56 Hz, 1H), 6.84 (d, *J* = 8.04 Hz, 1H), 6.04 (s, 2H). ^13^C{^1^H} NMR (100 MHz, CDCl_3_): δ 150.3, 148.3, 131.6, 129.5 (q, ^1^*J*(C,F) = 306.3 Hz), 116.2, 116.0 (q, ^2^*J*(C,F) = 2.1 Hz), 109.0. ^19^F NMR (375 MHz, CDCl_3_): δ −43.9 (s, 3F).

#### Naphthalen-2-yl(trifluoromethyl)sulfane (**3q**).^10c^

After purification by silica gel column chromatography (PE), compound **3q** was isolated as a white solid (27 mg, 48%). *R*_*f*_ (PE) = 0.9. ^1^H NMR (400 MHz, CDCl_3_): δ 8.21 (s, 1H), 7.89–7.87 (m, 3H), 7.67 (d, *J* = 8.5 Hz, 1H), 7.61–7.55 (m, 2H). ^13^C{^1^H} NMR (100 MHz, CDCl_3_): δ 137.0, 133.9, 133.4, 131.8, 129.7 (q, ^1^*J*(C,F) = 306.6 Hz), 129.2, 128.2, 127.9, 127.8, 127.0, 121.5 (q, ^2^*J*(C,F) = 2.3 Hz). ^19^F NMR (375 MHz, CDCl_3_): δ −42.5 (s, 3F).

#### Ethyl 5-((Trifluoromethyl)thio)-2,3-dihydrobenzofuran-2-carboxylate (**3r**)

After purification by silica gel column chromatography (PE/EA = 50:1), compound **3r** was isolated as a white solid (49 mg, 67%). *R*_*f*_ (PE/EA = 50:1) = 0.4. Mp = 102–103 °C. ^1^H NMR (400 MHz, CDCl_3_): δ 8.03 (s, 1H), 7.73–7.70 (m, 1H), 7.64–7.62 (m, 1H), 7.53 (s, 1H), 4.46 (q, *J* = 7.16 Hz, 2H), 1.43 (t, *J* = 7.1 Hz, 3H). ^13^C{^1^H} NMR (100 MHz, CDCl_3_): δ 159.0, 156.6, 147.2, 135.3, 131.8, 129.5 (q, ^1^*J*(C,F) = 306.5 Hz), 128.2, 119.5 (q, ^2^*J*(C,F) = 2.1 Hz), 113.5, 113.2, 61.8, 14.3. ^19^F NMR (375 MHz, CDCl_3_): δ −43.3 (s, 3F). HRMS (EM) *m*/*z*: [M −e]^+^ calcd for C_12_H_9_F_3_O_3_S, 290.0224; found, 290.0213.

#### 2-Methyl-5-((trifluoromethyl)thio)benzo[*d*]thiazole (**3s**)

After purification by silica gel column chromatography (PE/EA = 20:1), compound **3s** was isolated as a white solid (19 mg, 30%). *R*_*f*_ (PE/EA = 20:1) = 0.33. Mp = 87–88 °C. ^1^H NMR (400 MHz, CDCl_3_): δ 8.26 (d, *J* = 1.36 Hz, 1H), 7.87 (d, *J* = 8.28 Hz, 1H), 7.61 (dd, *J* = 1.48, 8.32 Hz, 1H), 2.86 (s, 3H). ^13^C{^1^H} NMR (100 MHz, CDCl_3_): δ 168.9, 153.9, 138.7, 131.8, 130.5, 129.6 (q, ^1^*J*(C,F) = 306.4 Hz), 122.1, 131.7, 20.2 (q, ^1^*J*(C,F) = 4.4 Hz). ^19^F NMR (375 MHz, CDCl_3_): δ – 42.9 (s, 3F). HRMS (EI) *m*/*z*: [M + H]^+^ calcd for C_9_H_7_F_3_NS_2_, 249.9967; found, 249.9970.

#### Methyl 5-((Trifluoromethyl)thio)nicotinate (**3t**).^24^

After purification by silica gel column chromatography (PE/EA = 10:1), compound **3t** was isolated as a white solid (25 mg, 43%). *R*_*f*_ (PE/EA = 5:1) = 0.5. ^1^H NMR (400 MHz, CDCl_3_): δ 9.33 (s,1H), 9.01 (s, 1H), 8.59 (s, 1H), 4.00 (s, 3H). ^13^C{^1^H} NMR (100 MHz, CDCl_3_): δ 164.4, 159.0, 152.5, 144.3, 128.9 (q, ^1^*J*(C,F) = 307.1 Hz), 126.9, 122.4, 52.9. ^19^F NMR (375 MHz, CDCl_3_): δ −42.0 (s, 3F).

#### 7-((Trifluoromethyl)thio)quinoline (**3u**)

After purification by silica gel column chromatography (PE/EA = 30:1), compound **3u** was isolated as a yellow oil (17 mg, 30%). *R*_*f*_ (PE/EA = 10:1) = 0.3. ^1^H NMR (400 MHz, CDCl_3_): δ 9.00 (d, *J* = 2.8 Hz, 1H), 8.48 (s, 1H), 8.21 (d, *J* = 8.2 Hz, 1H), 7.87 (d, *J* = 8.5 Hz, 1H), 7.75 (d, *J* = 8.5 Hz, 1H), 7.52–7.49 (s, 1H). ^13^C{^1^H} NMR (100 MHz, CDCl_3_): δ 151.5, 147.8, 137.8, 136.0, 132.3, 129.5 (q, ^1^*J*(C,F) = 306.8 Hz), 129.0, 128.9, 125.9 (q, ^2^*J*(C,F) = 1.8 H), 122.7. ^19^F NMR (375 MHz, CDCl_3_): δ −41.9 (s, 3F). HRMS (ESI) *m*/*z*: [M + H]^+^ calcd for C_10_H_7_F_3_NS, 230.0246; found, 230.0246.

#### 4-Methyl-7-((trifluoromethyl)thio)-2*H*-chromen-2-one (**3v**).^25^

After purification by silica gel column chromatography (PE/EA = 10:1), compound **3v** was isolated as a white solid (19 mg, 29%). *R*_*f*_ (PE/EA = 10:1) = 0.2. ^1^H NMR (400 MHz, CDCl_3_): δ 7.66–7.64 (m,2H), 7.55 (dd, *J* = 1.6, 8.2 Hz, 1H), 6.38 (d, *J* = 1.2 Hz,1H), 2.46 (d, *J* = 1.2 Hz, 3H). ^13^C{^1^H} NMR (100 MHz, CDCl_3_): δ 159.6, 153.3, 151.3, 130.8, 129.2 (q, ^1^*J*(C,F) = 306.8 Hz), 128.3 (q, ^2^*J*(C,F) = 2.1 Hz), 125.4, 123.9, 121.7, 116.7, 18.5. ^19^F NMR (375 MHz, CDCl_3_): δ −41.8 (s, 3F).

#### 9-Ethyl-3-((trifluoromethyl)thio)-9*H*-carbazole (**3w**).^6d^

After purification by silica gel column chromatography (PE), compound **3w** was isolated as a colorless solid (19 mg, 25%). *R*_*f*_ (PE) = 0.4. ^1^H NMR (400 MHz, CDCl_3_): δ 8.39 (d, *J* = 1.6 Hz, 1H), 8.12 (d, *J* = 7.8, Hz, 1H), 7.72 (dd, *J* = 1.6, 8.5 Hz, 1H), 7.55–7.51 (m, 1H), 7.45–7.72 (m, 2H), 7.31–7.27 (m, 1H), 5.14 (q, *J* = 7.2 Hz, 2H), 1.46 (t, *J* = 7.2 Hz, 3H). ^13^C{^1^H} NMR (100 MHz, CDCl_3_): δ 141.0, 140.3, 133.7, 129.9 (q, ^1^*J*(C,F) = 306.4 Hz), 129.6, 126.6, 123.9, 122.2, 120.7, 119.8, 112.5 (q, ^2^*J*(C,F) = 2.0 Hz), 109.2, 108.8, 37.7, 13.7. ^19^F NMR (375 MHz, CDCl_3_): δ −44.1 (s, 3F).

#### 5-((Trifluoromethyl)thio)benzo[*d*]thiazole (**3x**)

After purification by silica gel column chromatography (PE/EA = 50:1), compound **3x** was isolated as a colorless oil (12 mg, 20%). *R*_*f*_ (PE/EA = 50:1) = 0.5. ^1^H NMR (400 MHz, CDCl_3_): δ 9.09 (s,1H), 8.47 (d, *J* = 1.6 Hz, 1H), 8.02 (d, *J* = 8.4 Hz, 1H), 7.71 (dd, *J* = 1.6, 8.4 Hz, 1H). ^13^C{^1^H} NMR (100 MHz, CDCl_3_): δ 155.7, 153.7, 136.7, 132.6, 131.9, 129.5 (q, ^1^*J*(C,F) = 306.4 Hz), 122.7, 122.2 (q, ^2^*J*(C,F)= 2.1 Hz). ^19^F NMR (375 MHz, CDCl_3_): δ −42.7 (s, 3F). HRMS (ESI) *m*/*z*: [M + H]^+^ calcd for C_8_H_5_F_3_NS_2_, 235.9810; found, 235.9811.

#### Irradiation of **1k** in the Presence of TEMPO

A solution of arylazo sulfone **1k** (0.25 M) in DCE (1 mL) was placed in a 10 mL microwave tube, and then *S*-(trifluoromethyl) 4-fluorobenzenesulfonothioate (**2a**, 1 mmol, 4.0 equiv) and TEMPO (4 equiv) were added. The resulting solution was irradiated under stirring at room temperature for 36 h by means of a 21 W blue LED, and then the reaction mixture was concentrated under reduced pressure to evaporate the solvent. Purification by silica gel column chromatography (PE/EA = 30:1), afforded ethyl 4-((2,2,6,6-tetramethylpiperidin-1-yl)oxy)benzoate **4k** as a white solid (23 mg, 30% yield). *R_f_* (PE/EA = 30:1) = 0.4. ^1^H NMR (400 MHz, CDCl_3_): δ 7.93 (d, *J* = 9.1 Hz, 2H), 7.21 (s, 2H), 4.33 (q, *J* = 7.2 Hz, 2H), 1.64–1.56 (m, 5H), 1.44–1.41 (m, 1H), 1.36 (t, *J* = 7.2 Hz, 3H), 1.23 (s, 6H), 0.99 (s, 6H). ^13^C{^1^H} NMR (100 MHz, CDCl_3_): δ 167.4, 166.5, 131.0, 122.4, 113.7, 60.6, 60.4, 39.7, 32.4, 20.4, 17.0, 14.4.

## Supporting Information Available

Mechanistic studies and spectroscopic data for compounds **1**, **2c**, and **3** (PDF)
