# Supplementary material for: Metal-Free Trifluoromethylthiolation of Arylazo Sulfones
Source: J Org Chem. 2020 Dec 22;86(1):1292–9. doi: 10.1021/acs.joc.0c02669 (PMC8765700; doi:10.1021/acs.joc.0c02669)
Supplement: Supplementary file 1 — jo0c02669_si_001.pdf [file jo0c02669_si_001.pdf]

Metal-Free Trifluoromethylthiolation of Arylazo Sulfones

Ankun Li,[a,§] Yuxuan Li, [a,c,§] Junjie Liu, [a,c] Jingqi Chen,[a] Kui Lu,[c] Di Qiu,[a] Maurizio Fagnoni,[b] Stefano Protti*,[b] and Xia Zhao*,[a]

[a]*Tianjin Key Laboratory of Structure and Performance for Functional Molecules, MOE Key Laboratory of Inorganic-Organic Hybrid Functional Materials Chemistry, College of Chemistry, Tianjin Normal University, Tianjin 300387, P. R. China.*

[b]*PhotoGreen Lab, Department of Chemistry, University of Pavia, V. Le Taramelli 12, Pavia, Italy.*

[c]*College of Biotechnology, Tianjin University of Science & Technology, Tianjin 300457, China.*

[§]*These authors contributed equally to this work.*

CONTENTS

1) Mechanistic insight into the visible-light driven trifluoromethylthiolation process **S2**

2) Copy of the ^{1}H, ^{13}C and ^{19}F NMR spectra of the prepared compounds **S4**

1. **Mechanistic insight into the visible-light driven trifluoromethylthiolation process.**

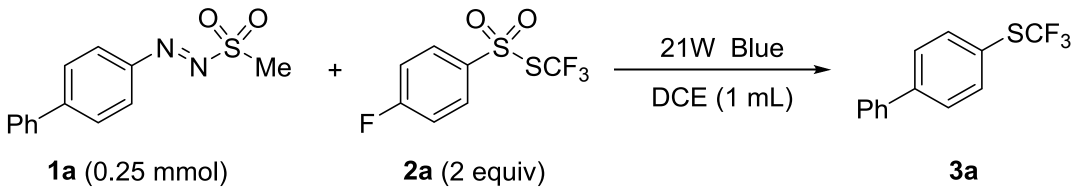

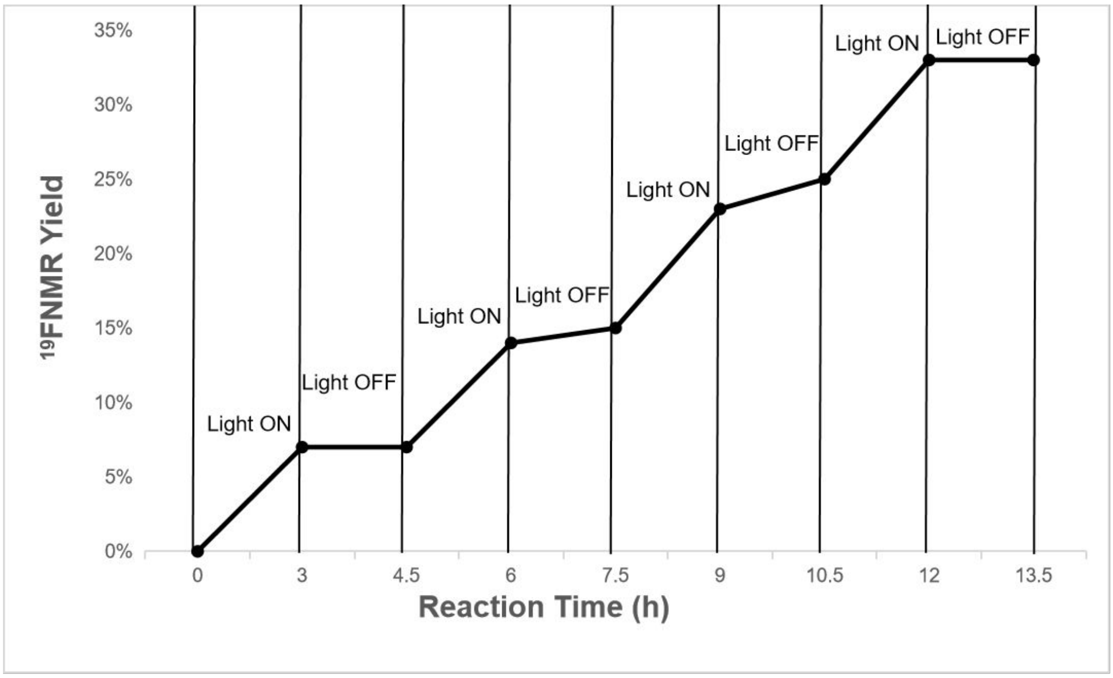

Figure S1. On-Off experiments carried out on the visible-light driven formation of arylthiotrifluoromethyl derivative **3a** from aryl azosulfone **1a.**

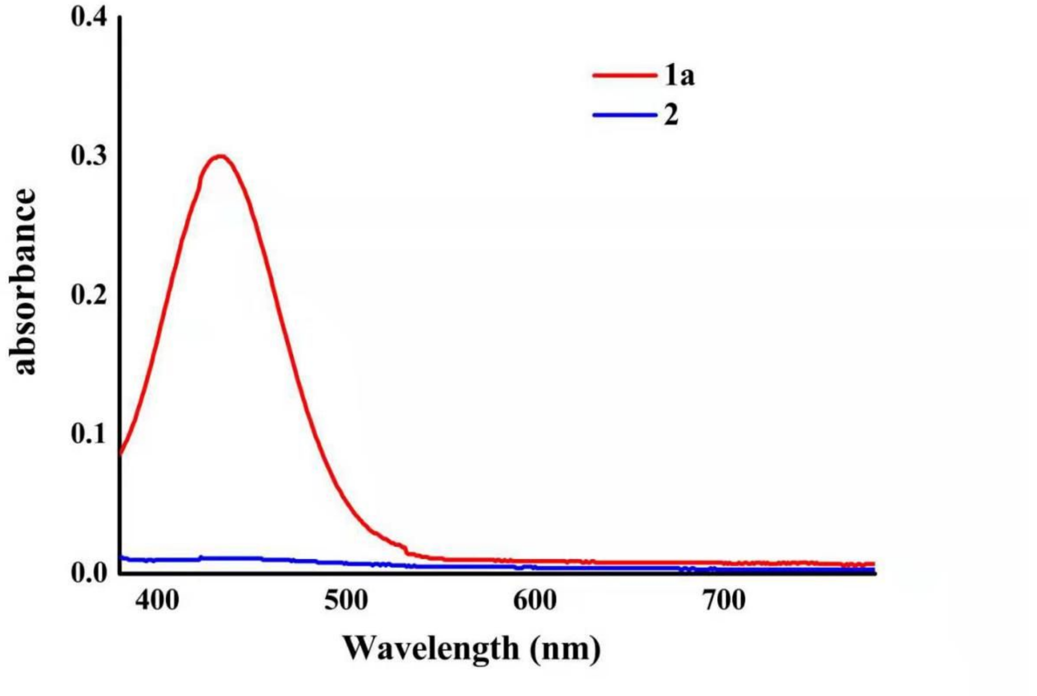

Figure S2. UV/Vis absorption spectra of DCE solutions of ethyl 4-((methylsulfonyl)diazenyl)benzoate (**1a**, 4×10^{-4} M, Red Line) and *S*-(trifluoromethyl) 4-fluorobenzenesulfonothioate (**2a**, 8×10^{-4} M, Blue Line).

2. Copy of the ^1H, ^{13}C and ^{19}F NMR spectra of the prepared compounds

^1H NMR spectra of ***1-([1,1'-Biphenyl]-4-yl)-2-(methylsulfonyl)diazene (1a)*** in $CDCl_3$

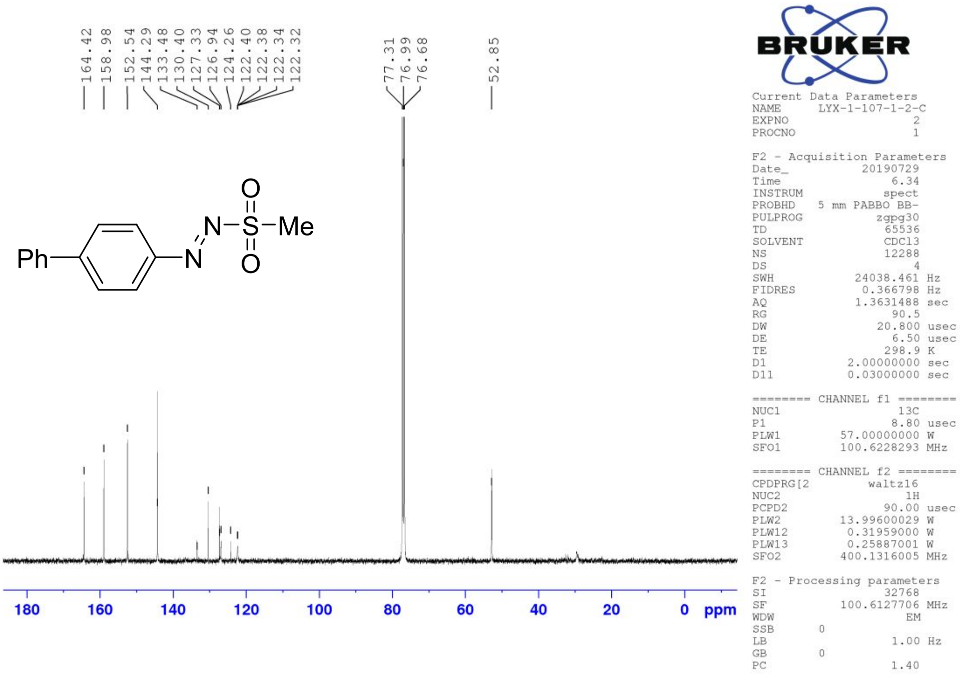

^1H, and ^{13}C NMR spectra of ***1-([1,1'-biphenyl]-4-yl)-2-((4-nitrophenyl)sulfonyl)diazene (1a')*** in $CDCl_3$

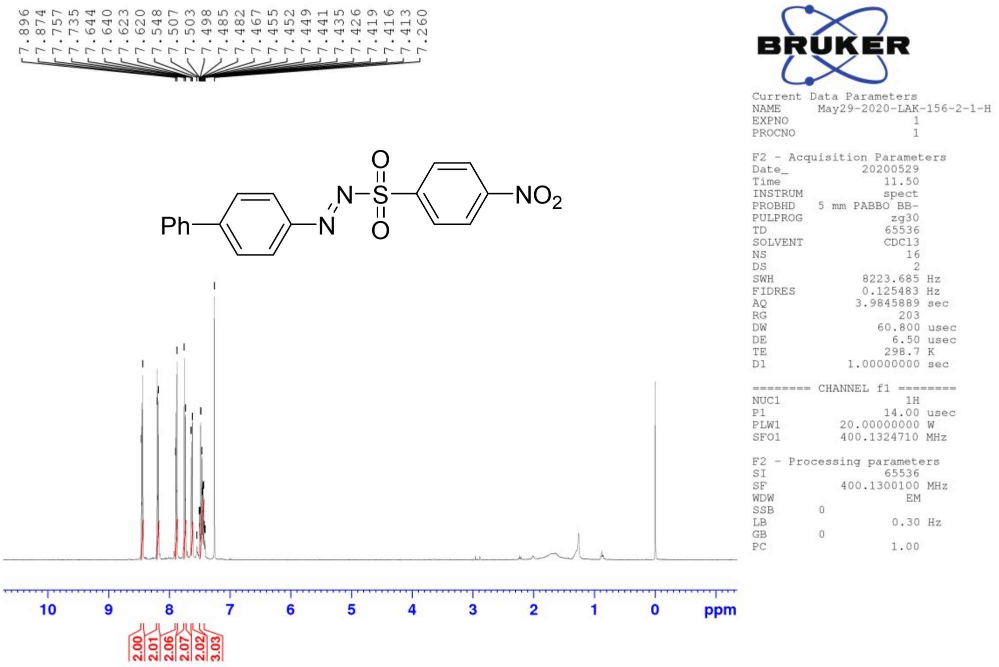

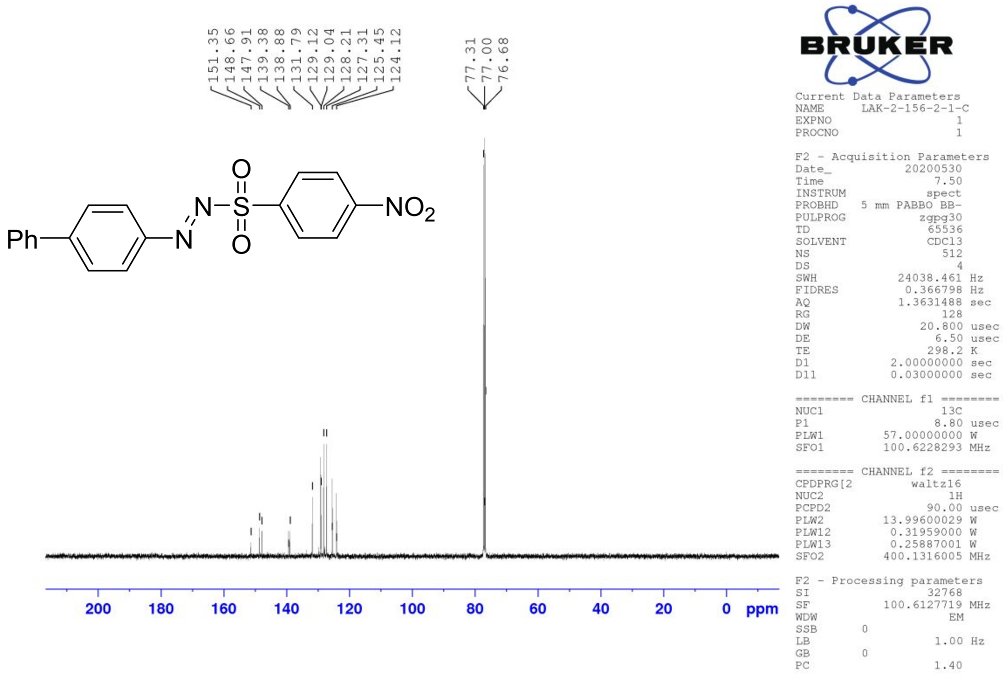

^{1}H, and ^{13}C NMR spectra of ***4-(4-((methylsulfonyl)diazenyl)phenyl)morpholine (1b)*** in $CDCl_3$

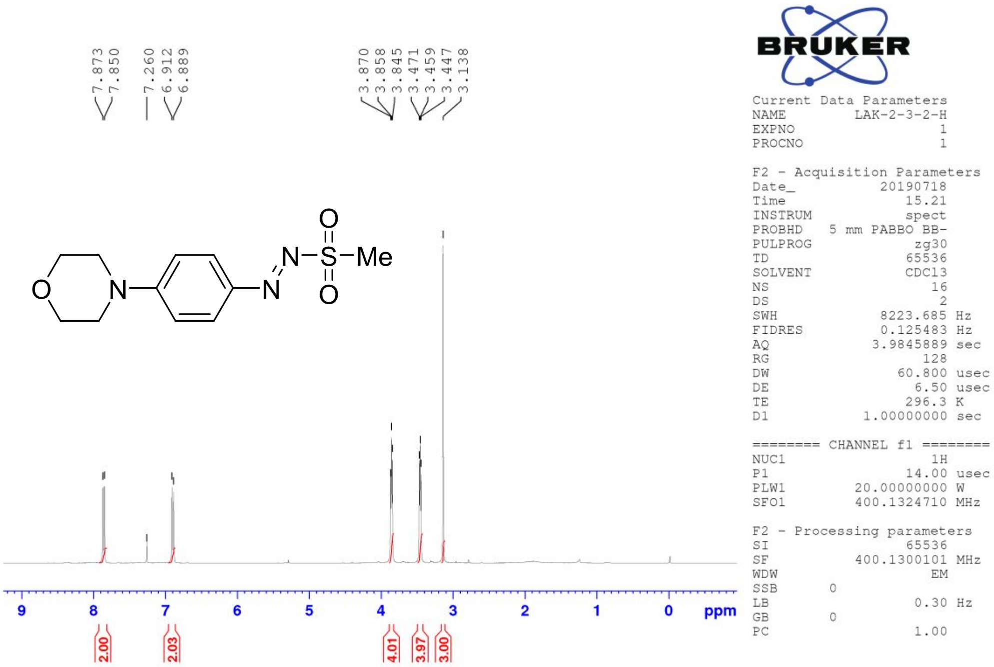

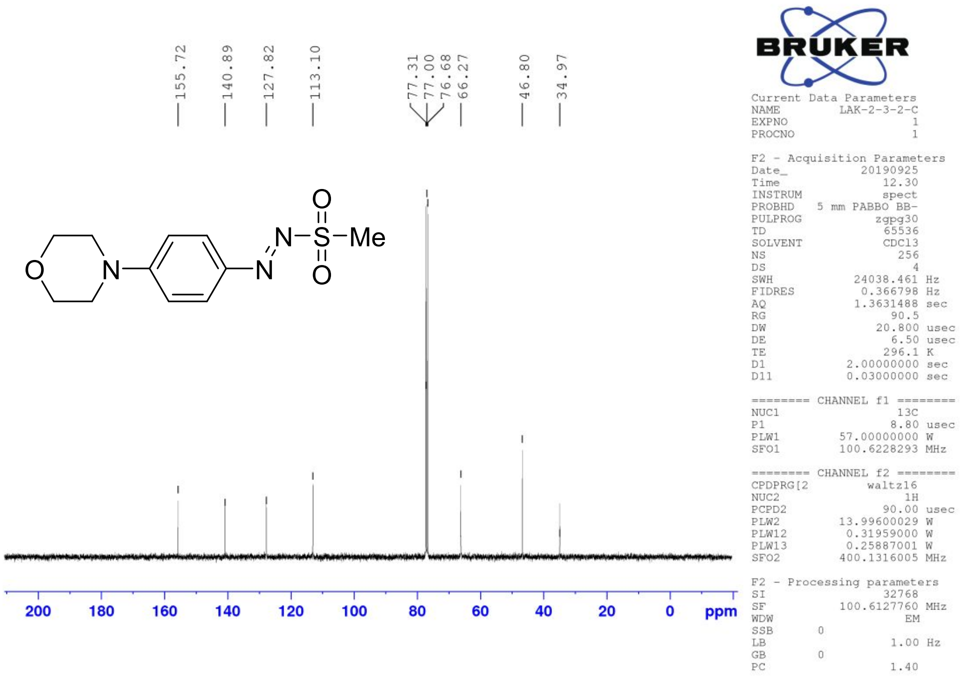

^{1}H, and ^{13}C NMR spectra of ***1-(methylsulfonyl)-2-(4-phenoxyphenyl)diazene (1c)*** in $CDCl_3$

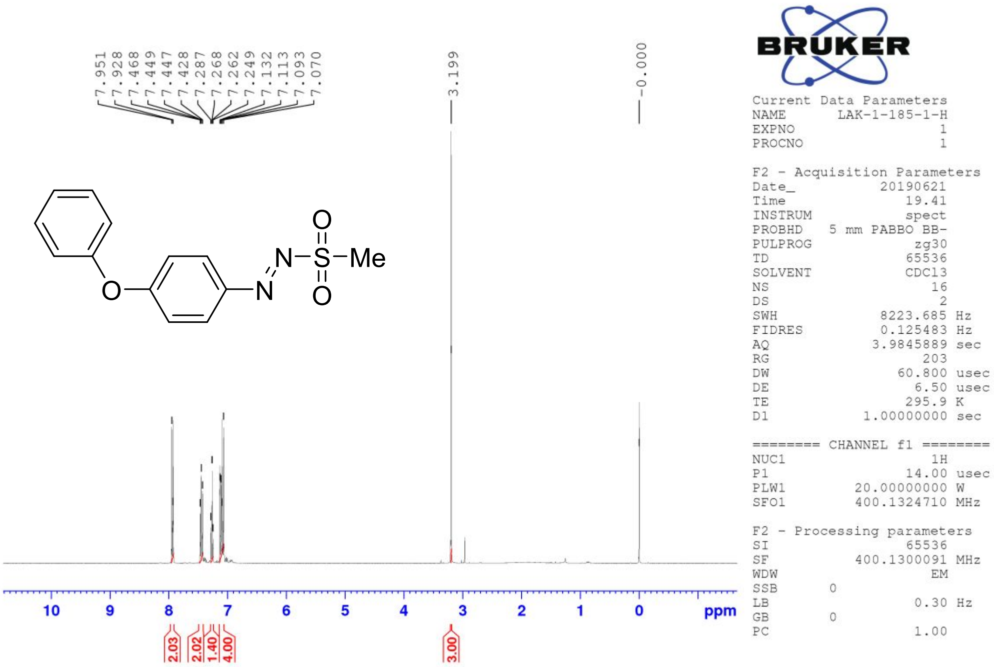

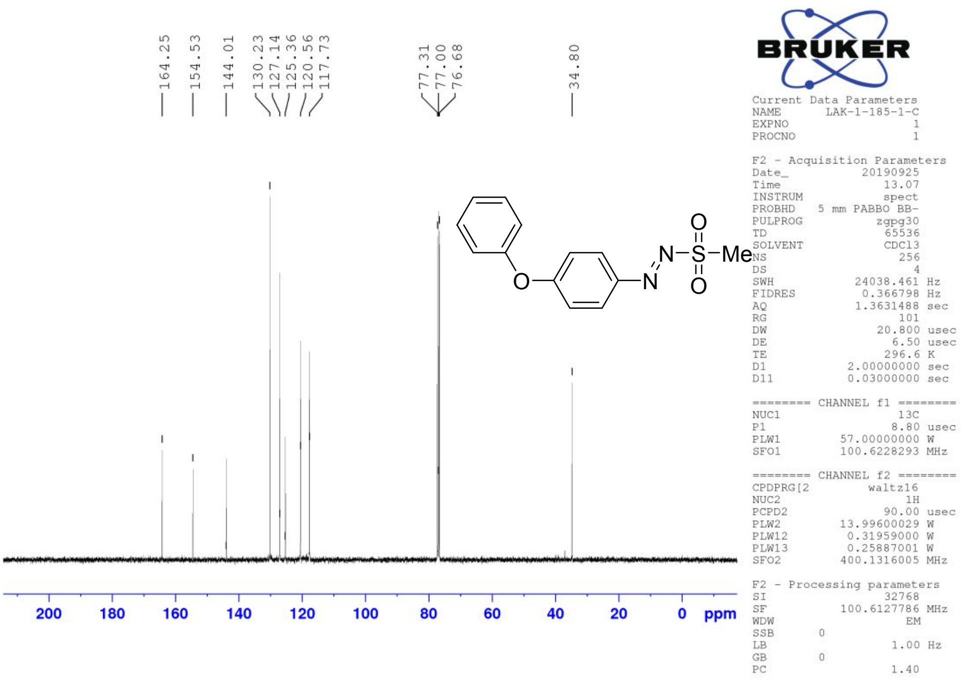

^{1}H, and ^{13}C NMR spectra of ***(4-(benzyloxy)phenyl)-2-(methylsulfonyl)diazene (1d)*** in $CDCl_3$

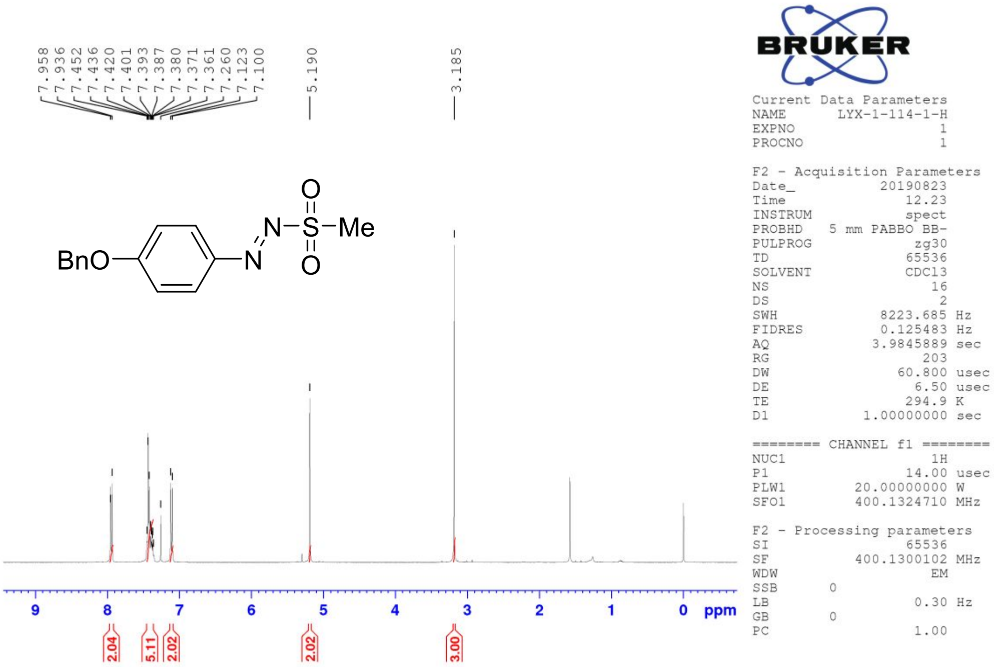

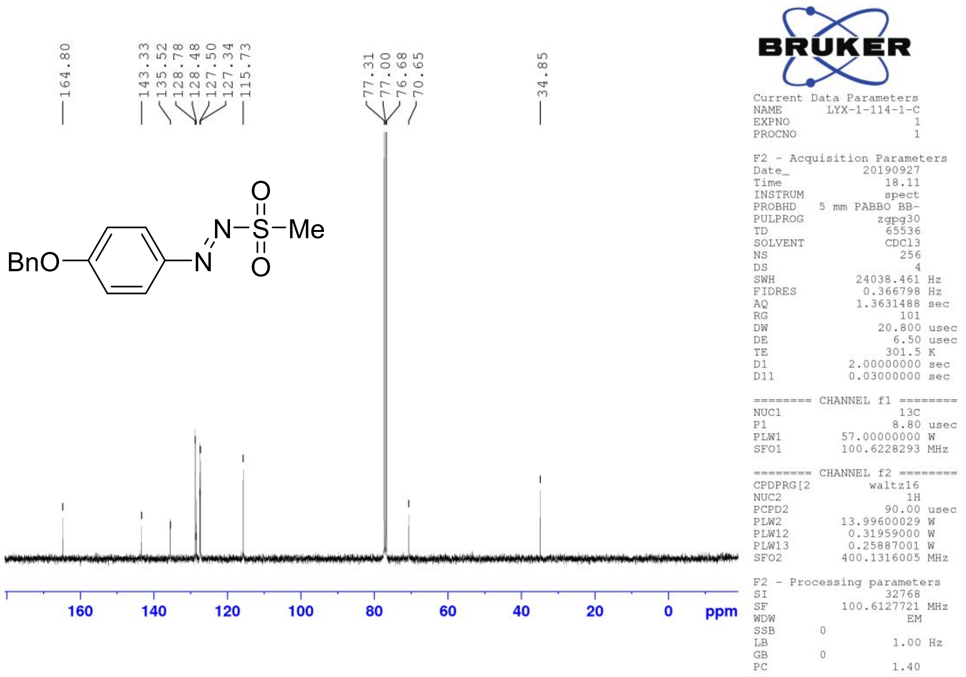

^{1}H, and ^{13}C NMR spectra of ***1-(methylsulfonyl)-2-(4-(methylthio)phenyl)diazene (1e)*** in $CDCl_3$

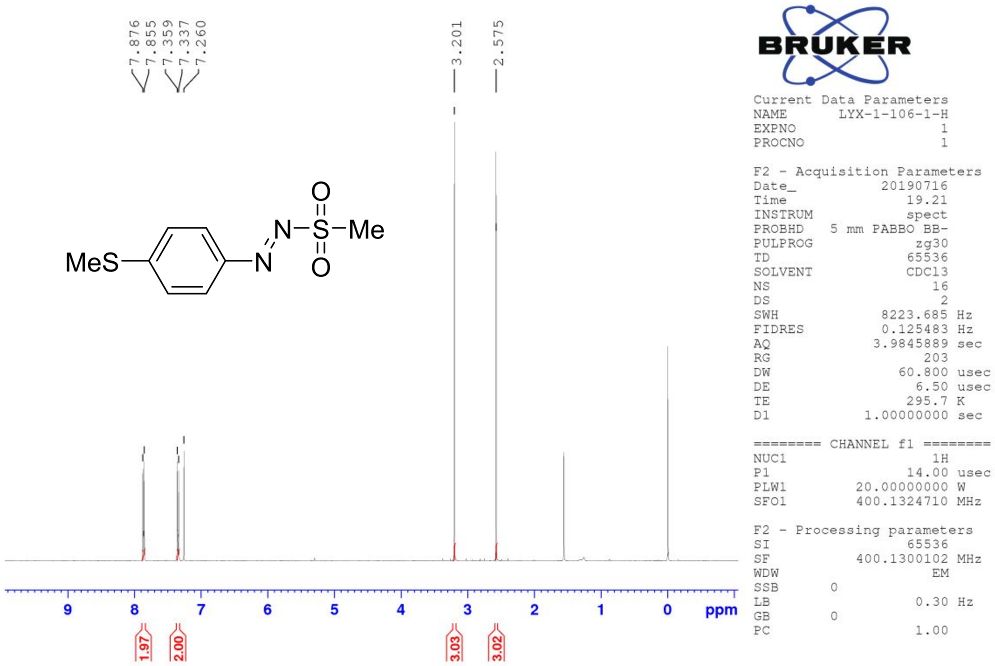

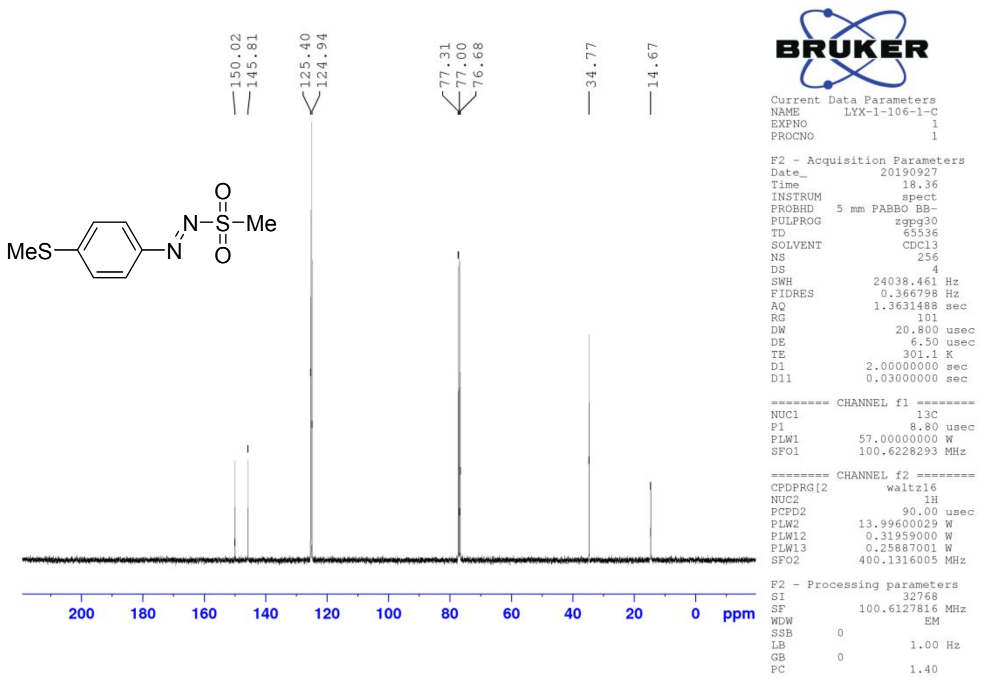

^{1}H NMR spectra of ***1-(4-(tert-Butyl)phenyl)-2-(methylsulfonyl)diazene (1f)*** in $CDCl_3$

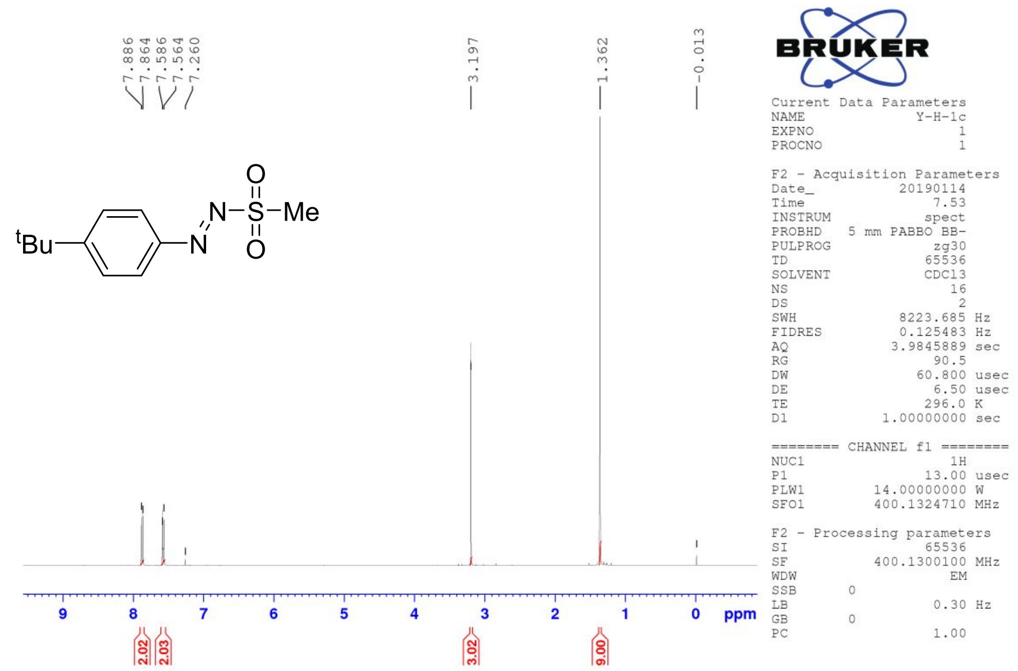

^{1}H NMR spectra of ***Ethyl 3-(4-((E)-(methylsulfonyl)diazenyl)phenyl)acrylate (1g)*** in $CDCl_3$

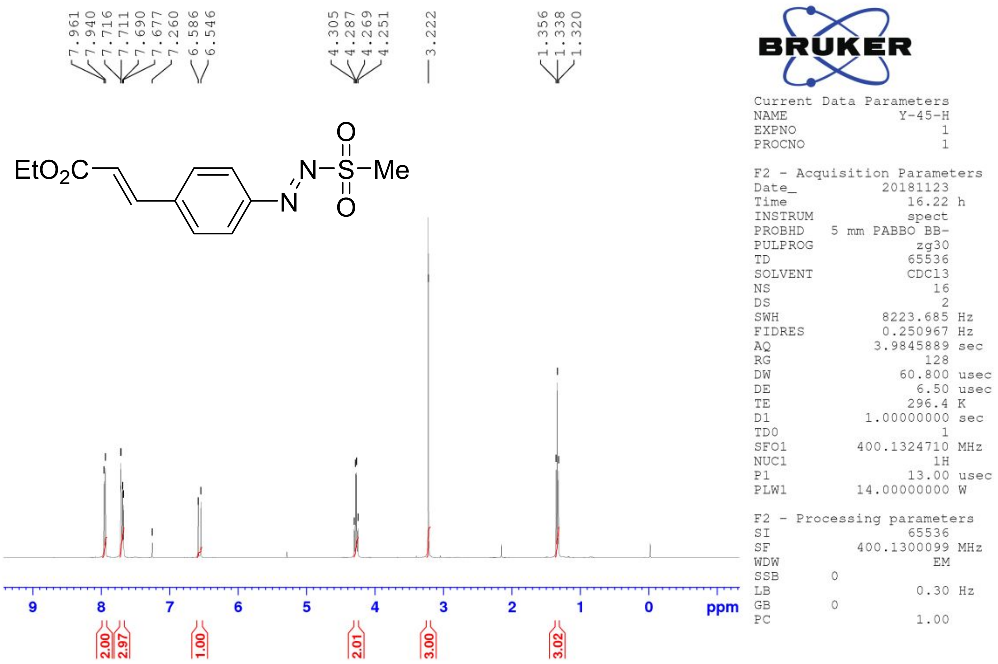

^{1}H NMR spectra of ***1-(Methylsulfonyl)-2-(4-((triisopropylsilyl)ethynyl)phenyl)diazene (1h)*** in $CDCl_3$

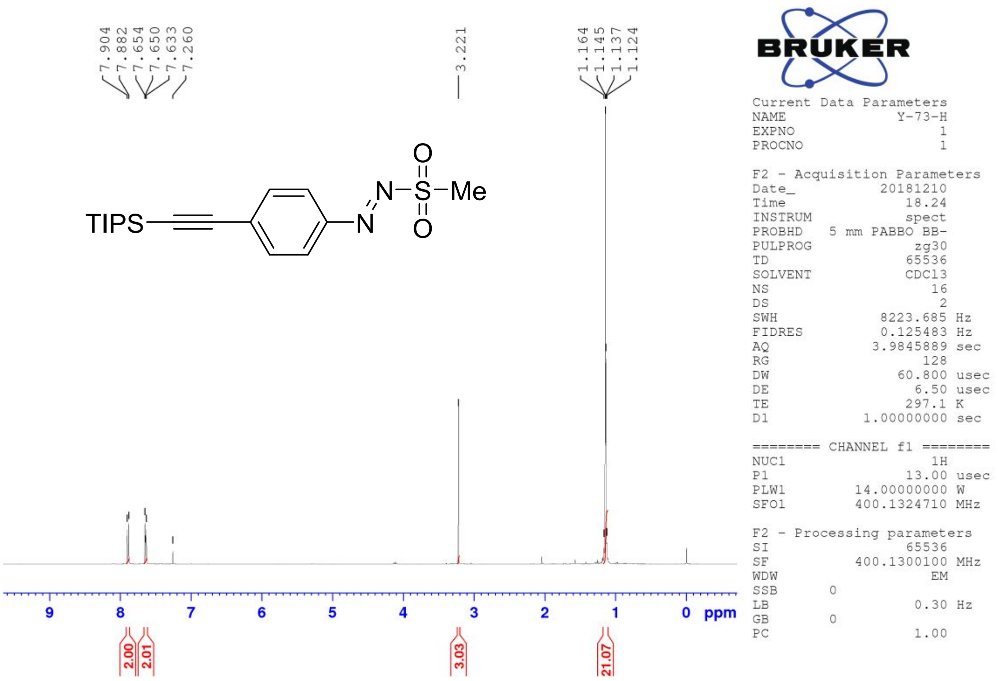

^{1}H NMR spectra of ***1-(4-Bromophenyl)-2-(methylsulfonyl)diazene (1i)*** in $CDCl_3$

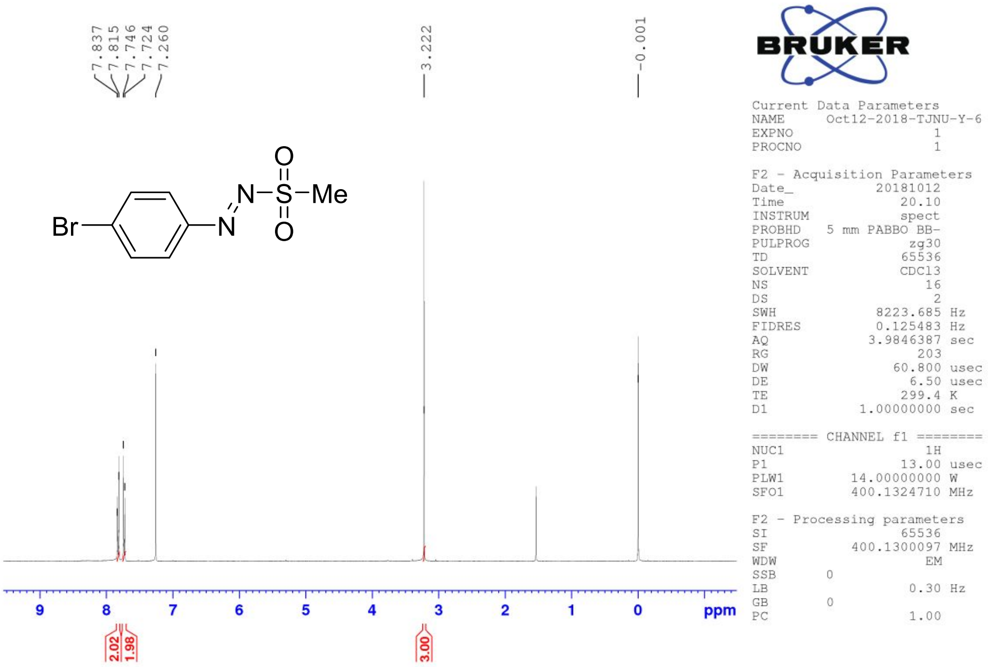

^{1}H NMR spectra of ***4-((Methylsulfonyl)diazenyl)benzonitrile (1j)*** in $CDCl_3$

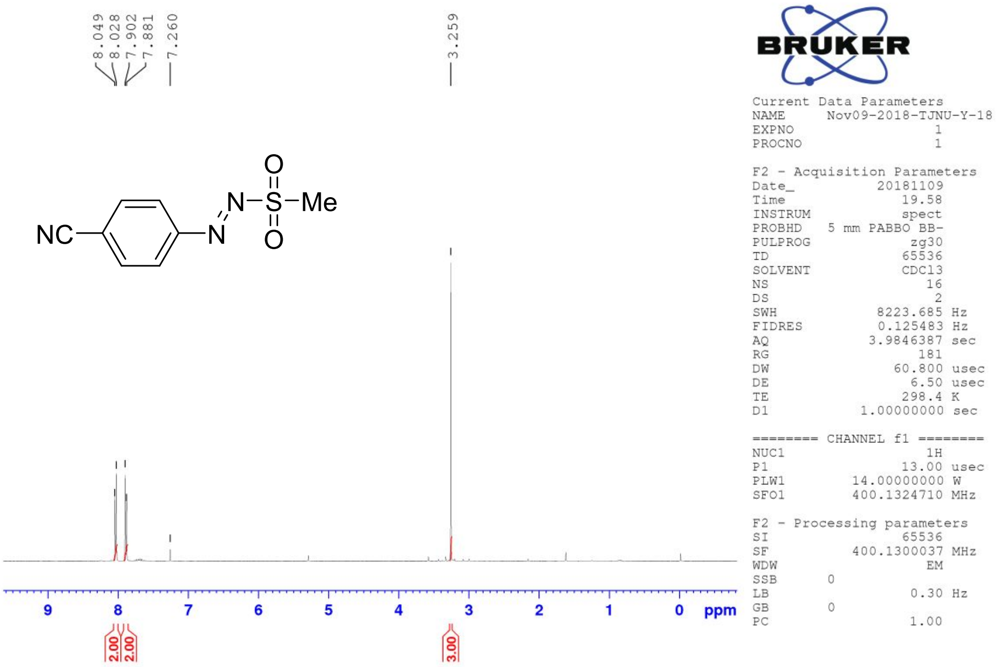

^{1}H NMR spectra of ***Ethyl 4 -((methylsulfonyl)diazenyl)benzoate (1k)*** in $CDCl_3$

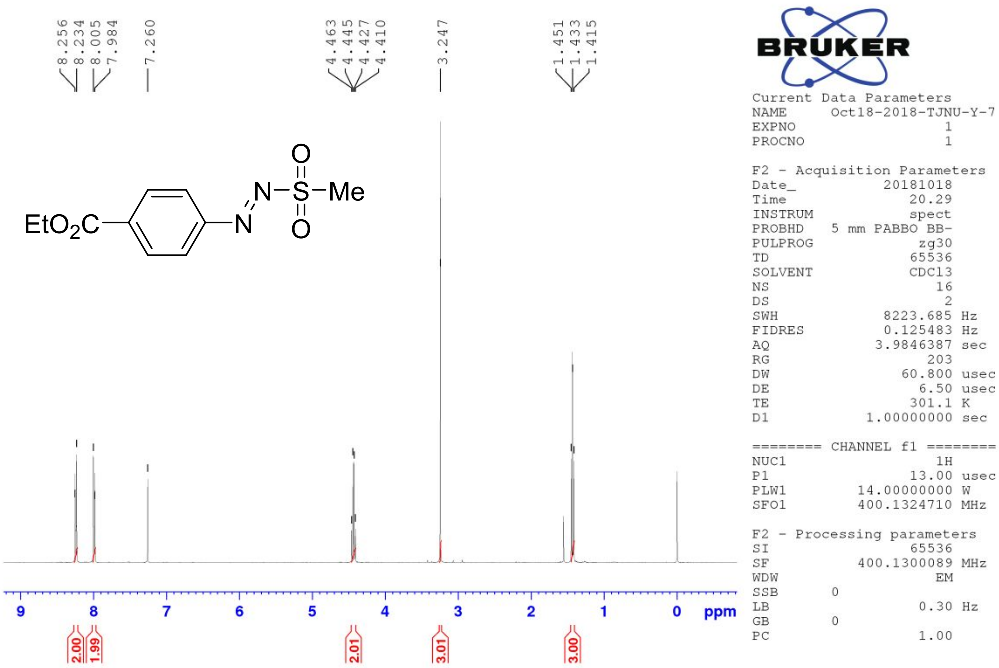

^{1}H NMR spectra of ***1-(4-((Methylsulfonyl)diazenyl)phenyl)ethanone (1l)*** in $CDCl_3$

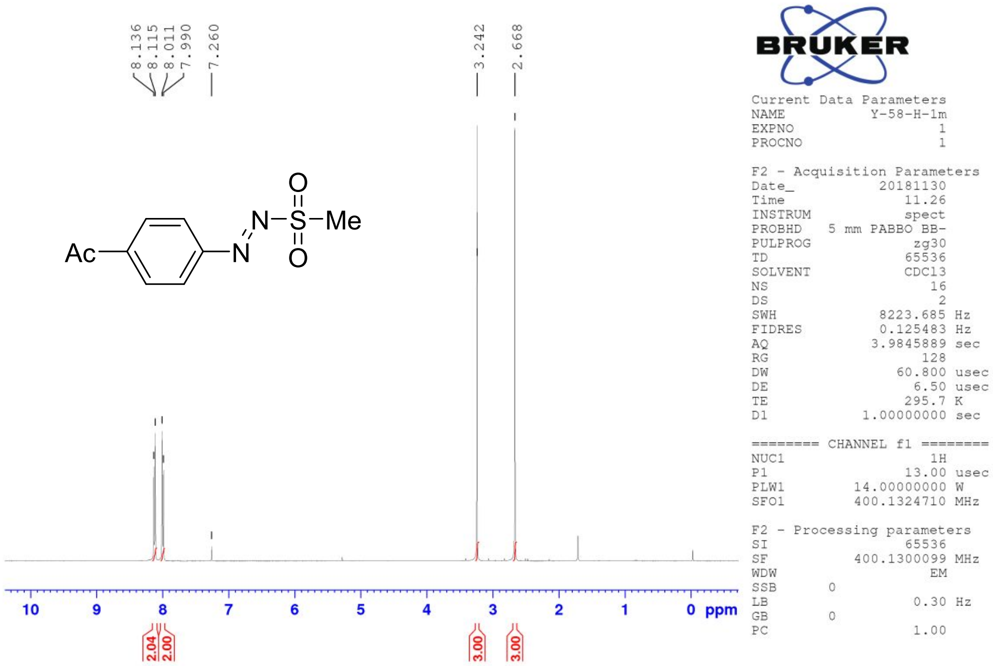

^{1}H, and ^{13}C NMR spectra of ***(4-((methylsulfonyl)diazenyl)phenyl)(phenyl)methanone (1m)*** in $CDCl_3$

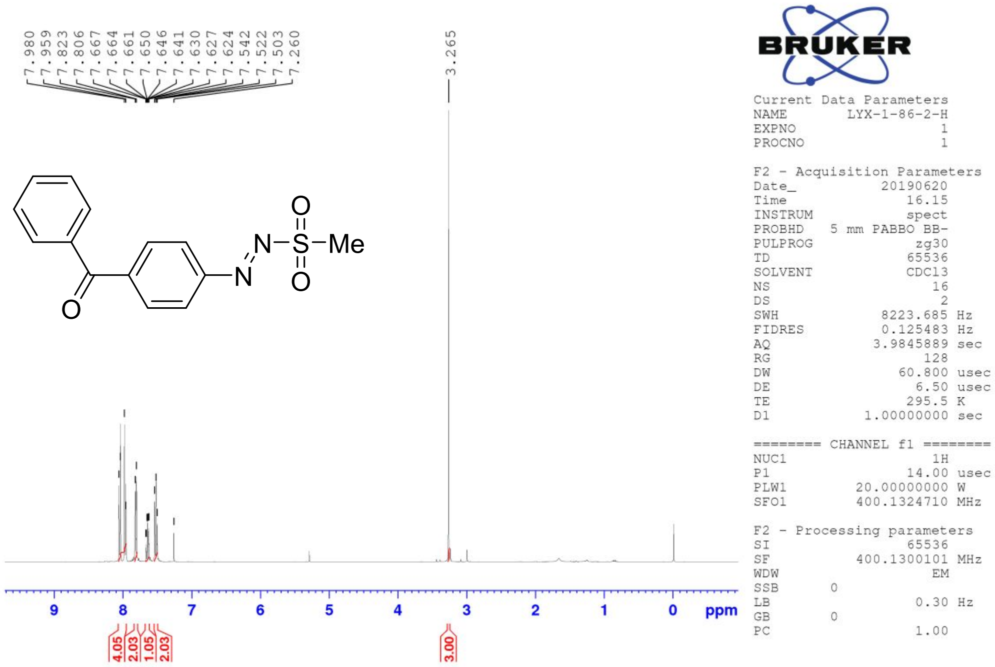

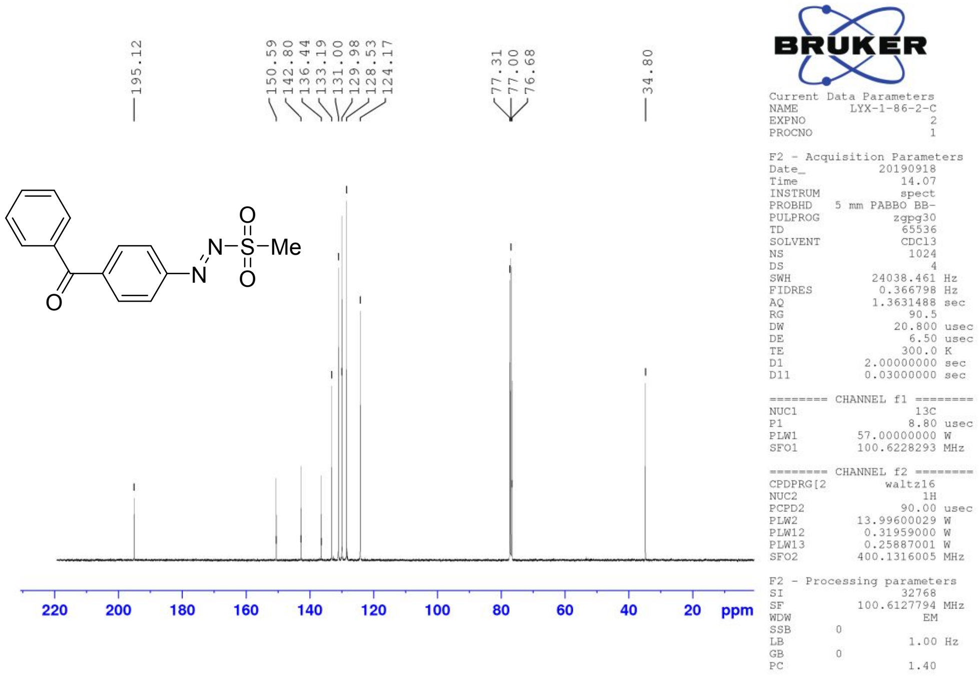

^{1}H NMR spectra of ***1-(3-Bromophenyl)-2-(methylsulfonyl)diazene (1n)*** in $CDCl_3$

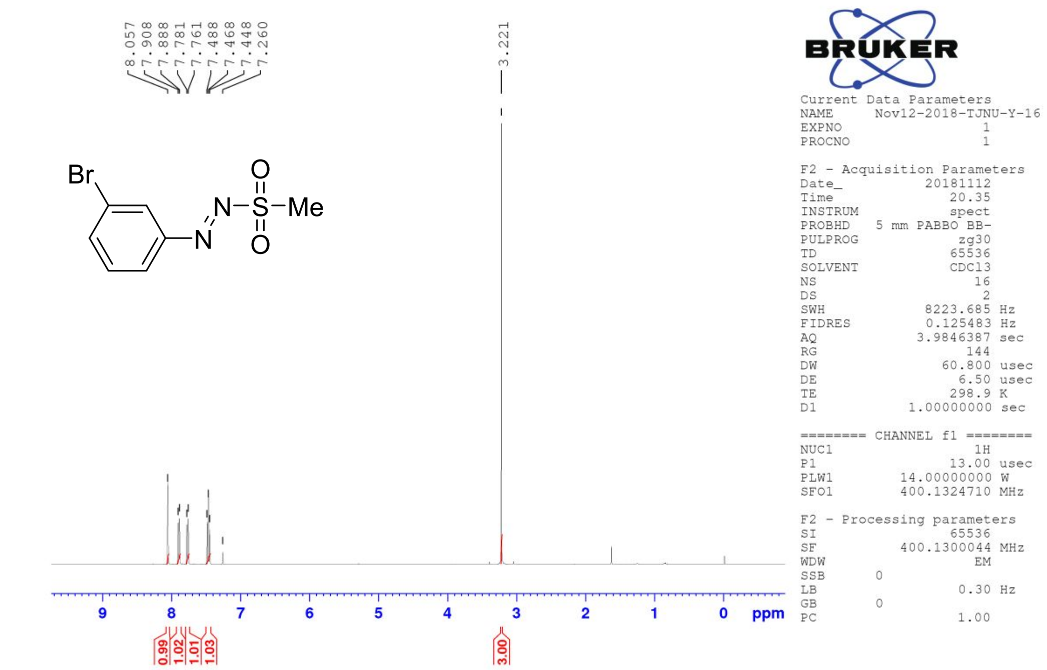

^{1}H, and ^{13}C NMR spectra of ***1-(methylsulfonyl)-2-(3,4,5-trimethoxyphenyl)diazene (1o)*** in $CDCl_3$

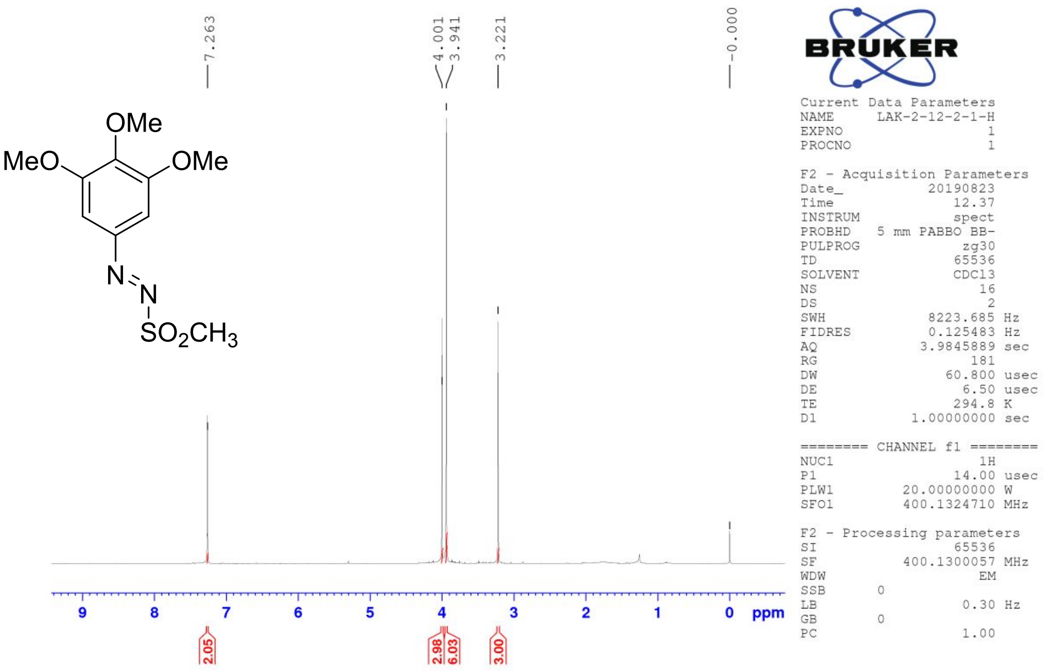

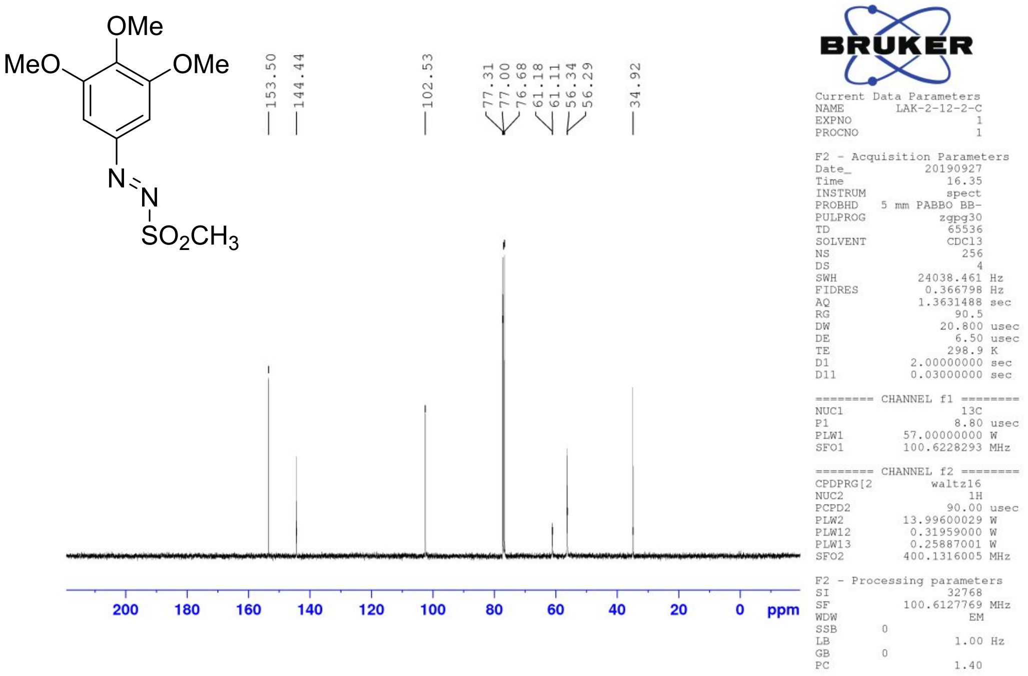

^{1}H, and ^{13}C NMR spectra of ***1-(benzo[d][1,3]dioxol-5-yl)-2-(methylsulfonyl)diazene (1p)*** in $CDCl_3$

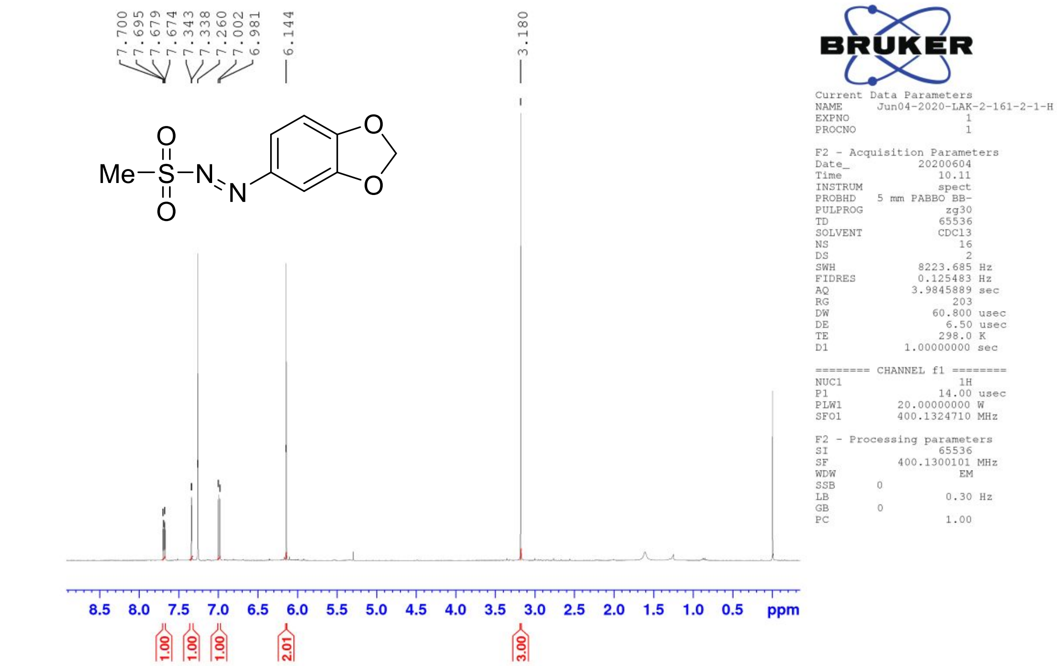

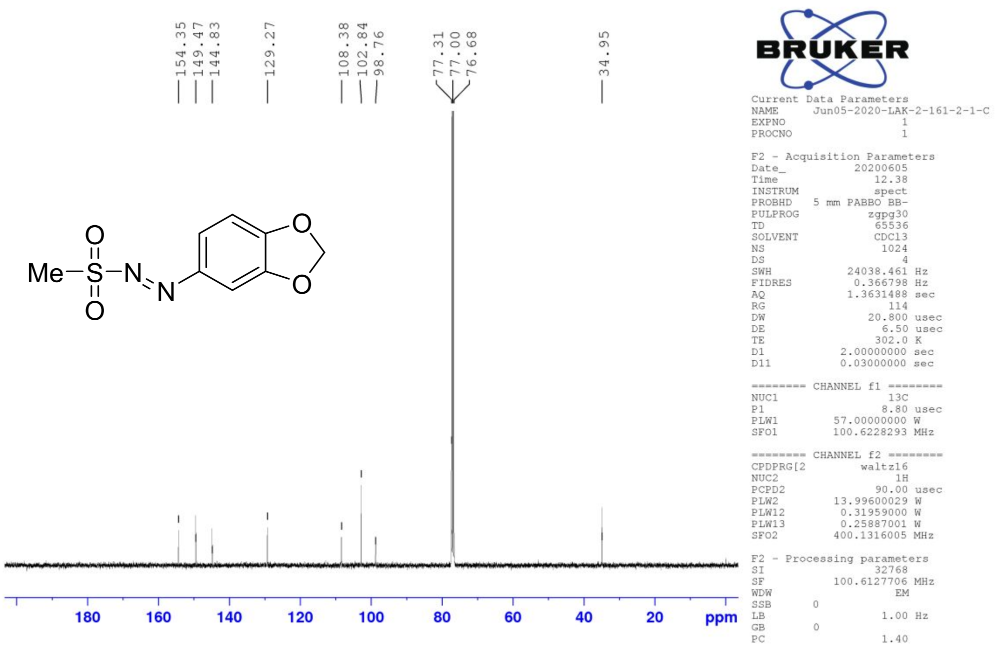

^{1}H NMR spectra of ***1-(Methylsulfonyl)-2-(naphthalen-2-yl)diazene (1q)*** in $CDCl_3$

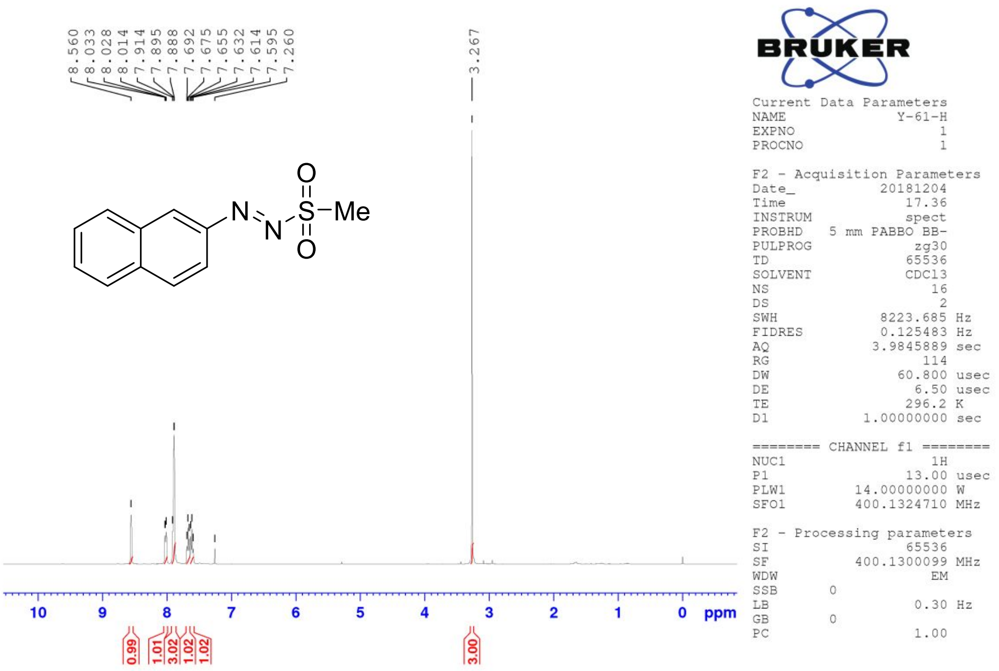

^{1}H NMR spectra of ***Ethyl 5-((methylsulfonyl)diazenyl)benzofuran-2-carboxylate (1r)*** in $CDCl_3$

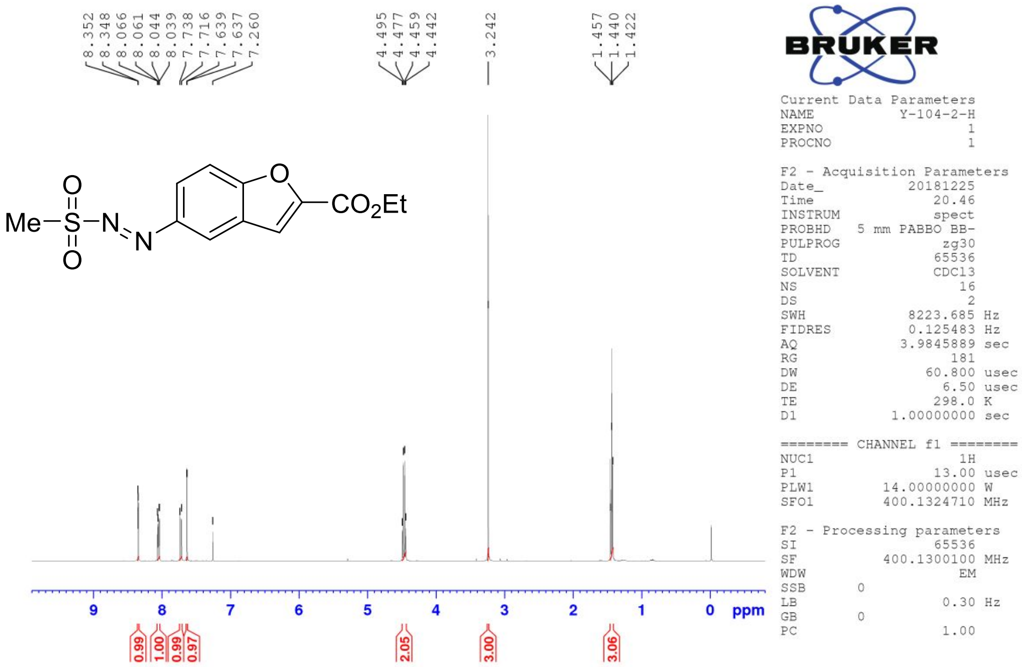

^{1}H NMR spectra of ***2-Methyl-5-((methylsulfonyl)diazenyl)benzo[d]thiazole (1s)*** in $CDCl_3$

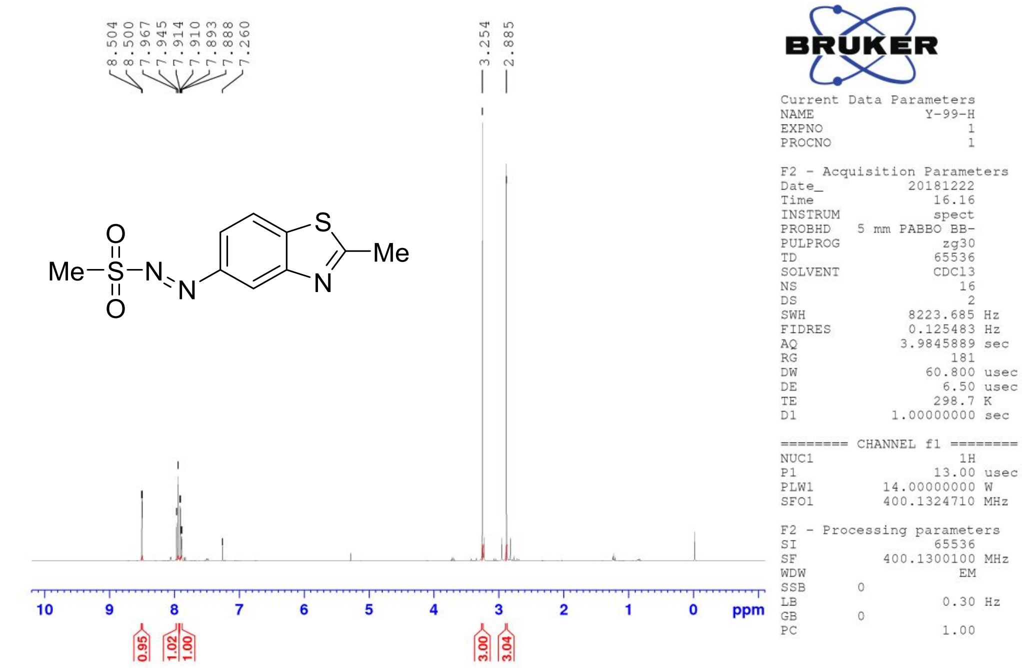

^1H NMR spectra of ***Methyl 5-((methylsulfonyl)diazenyl)nicotinate (1t)*** in $CDCl_3$

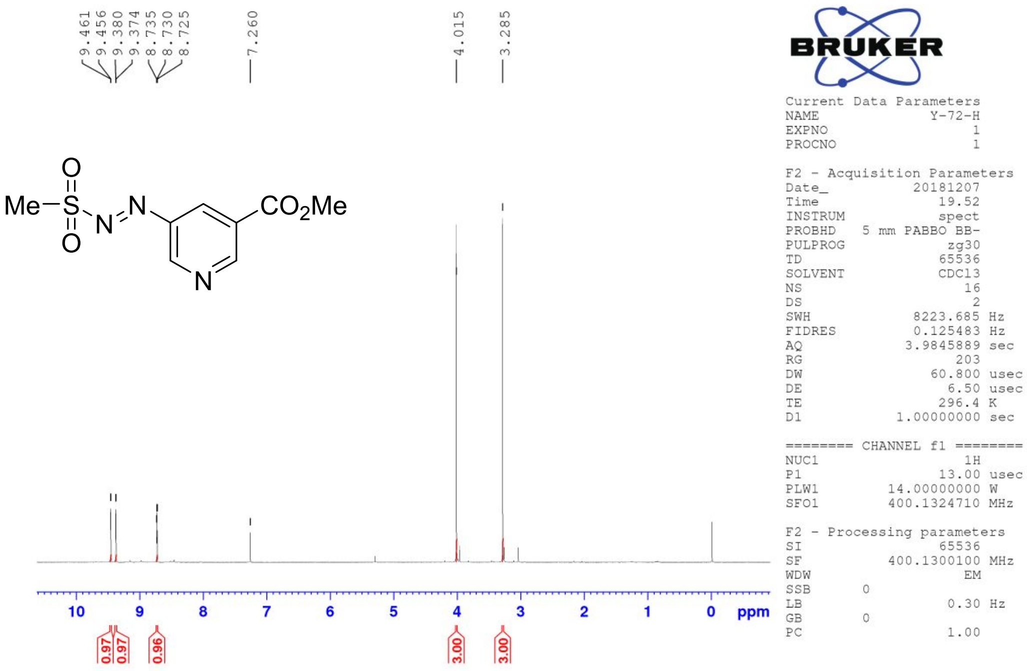

^1H, and ^{13}C NMR spectra of ***7-((methylsulfonyl)diazenyl)quinoline (1u)*** in $CDCl_3$

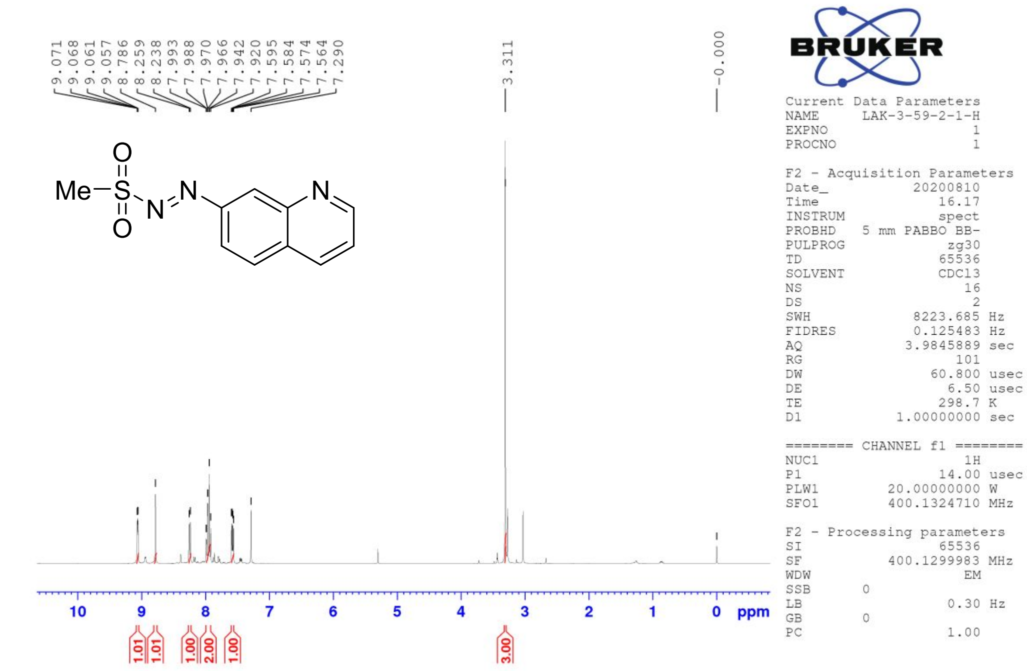

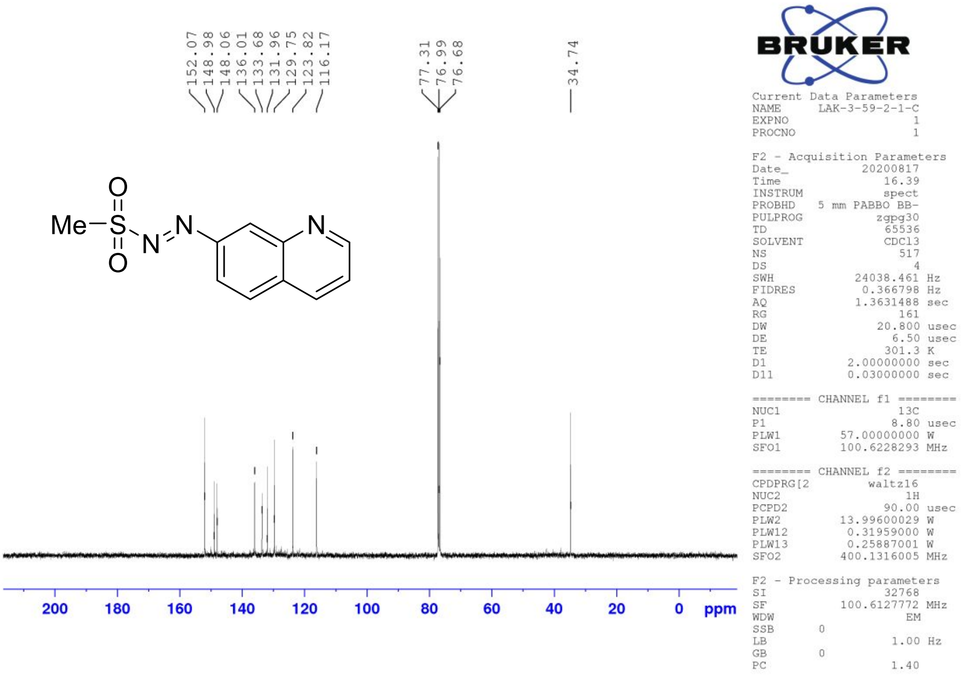

^{1}H, and ^{13}C NMR spectra of ***4-methyl-7-((methylsulfonyl)diazenyl)-2H-chromen-2-one (1v)*** in $CDCl_3$

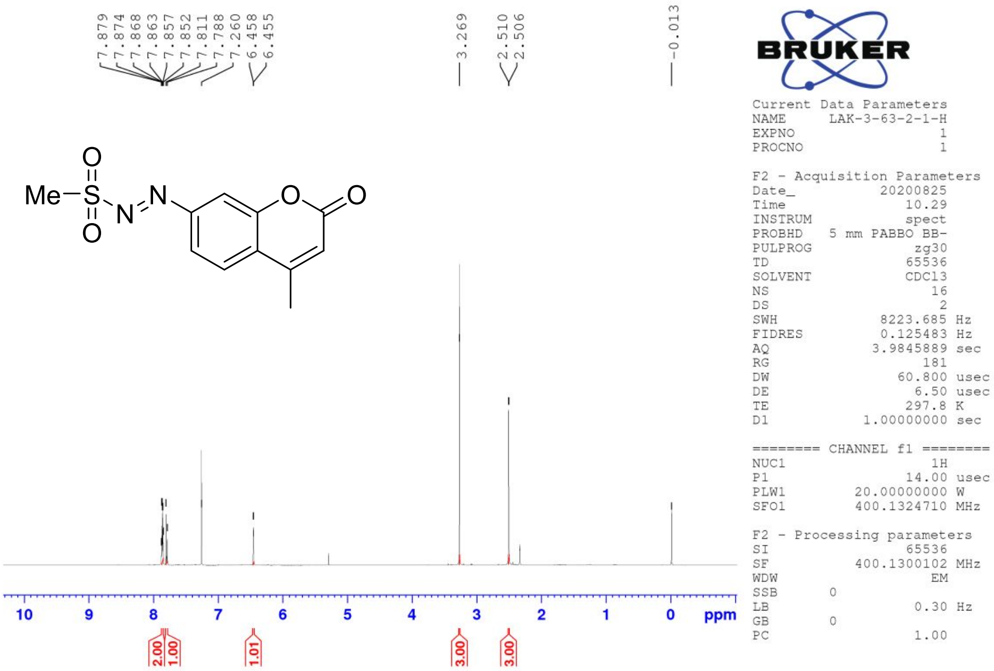

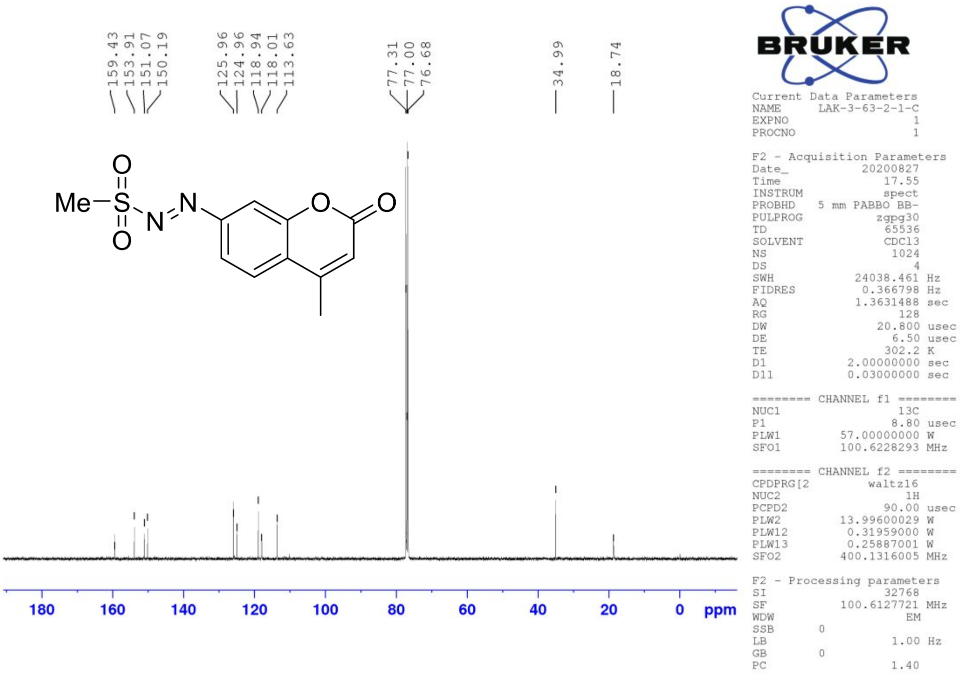

^{1}H, and ^{13}C NMR spectra of ***9-ethyl-3-((methylsulfonyl)diazenyl)-9H-carbazole (1w)*** in $CDCl_3$

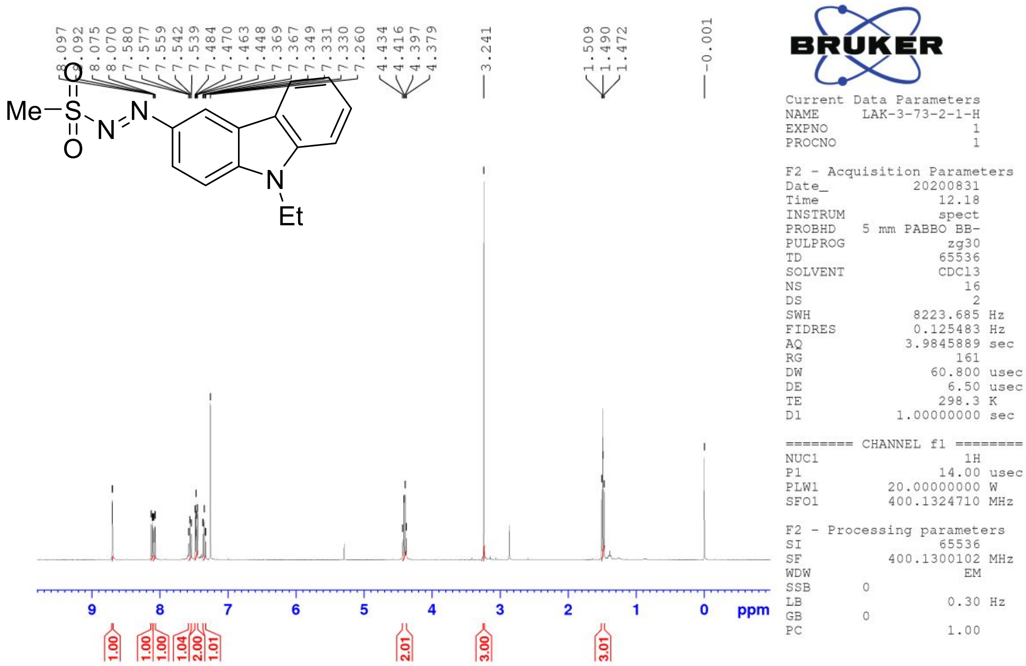

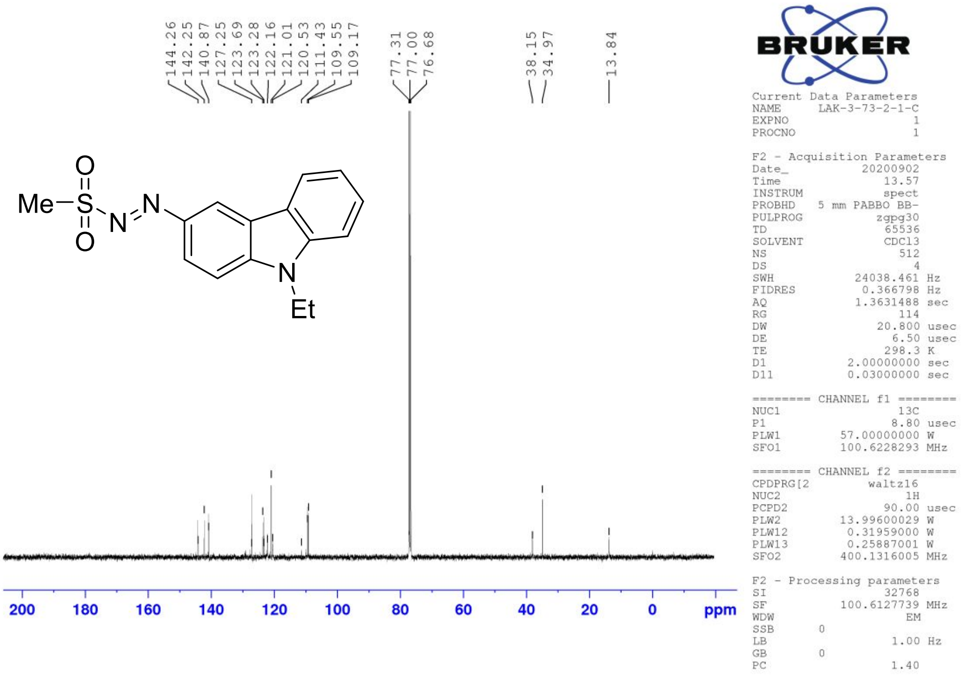

^{1}H, and ^{13}C NMR spectra of ***5-((methylsulfonyl)diazenyl)benzo[d]thiazole (1x)*** in $CDCl_3$

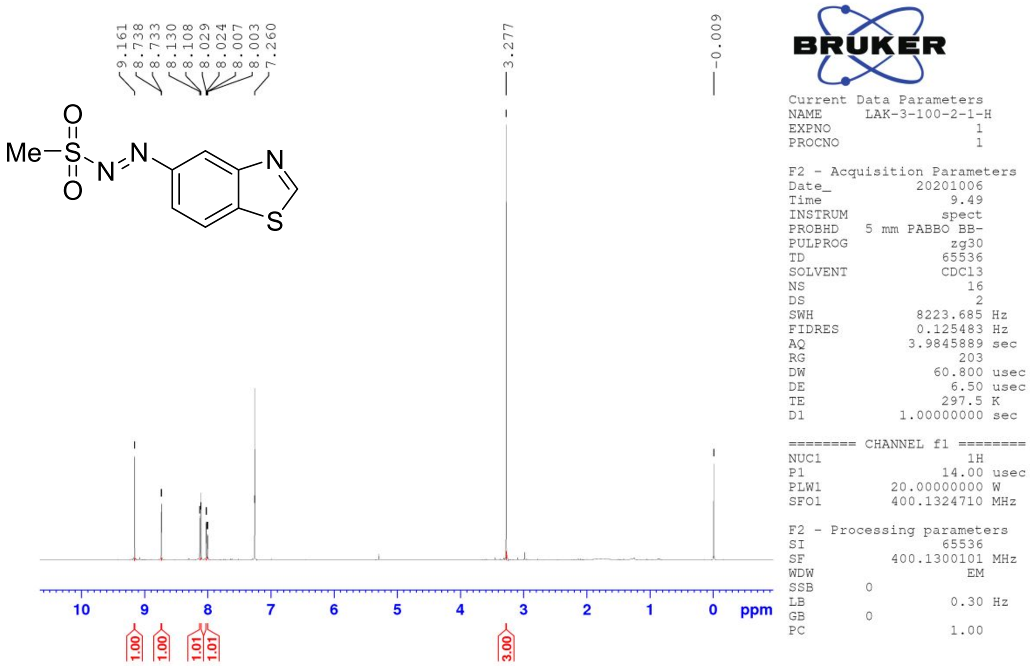

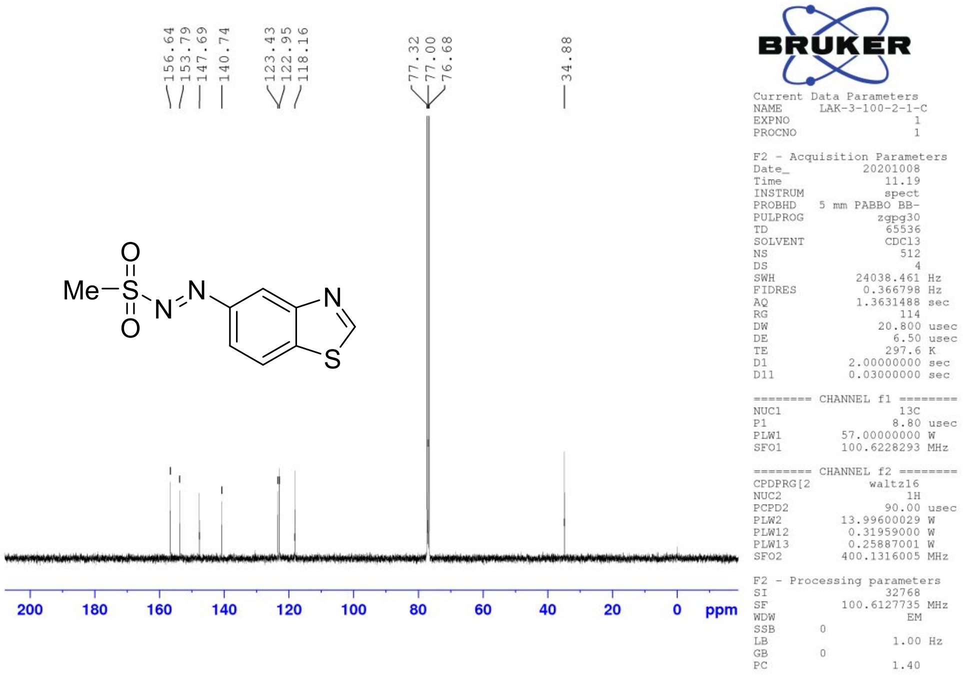
156.64
153.79
147.69
140.74
123.43
122.95
118.16
77.32
77.00
76.68
34.88
Me
S
O
O
N
N
N
S
200 180 160 140 120 100 80 60 40 20 0 ppm
BRUKER
Current Data Parameters
NAME LAK-3-100-2-1-C
EXPNO 1
PROCNO 1
F2 - Acquisition Parameters
Date_ 20201008
Time 11.19
INSTRUM spect
PROBHD 5 mm PABBO BB-
PULPROG zgpg30
TD 65536
SOLVENT CDCl3
NS 512
DS 4
SWH 24038.461 Hz
FIDRES 0.366798 Hz
AQ 1.3631488 sec
RG 114
DW 20.800 usec
DE 6.50 usec
TE 297.6 K
D1 2.00000000 sec
D11 0.03000000 sec
======== CHANNEL f1 ========
NUC1 13C
P1 8.80 usec
PLW1 57.00000000 W
SFO1 100.6228293 MHz
======== CHANNEL f2 ========
CPDPRG[2 waltz16
NUC2 1H
PCPD2 90.00 usec
PLW2 13.99600029 W
PLW12 0.31959000 W
PLW13 0.25887001 W
SFO2 400.1316005 MHz
F2 - Processing parameters
SI 32768
SF 100.6127735 MHz
WDW EM
SSB 0
LB 1.00 Hz
GB 0
PC 1.40

^{1}H, ^{13}C and ^{19}F NMR spectra of ***S-(trifluoromethyl) 3,5-difluorobenzenesulfonothioate (2c)*** in $CDCl_3$

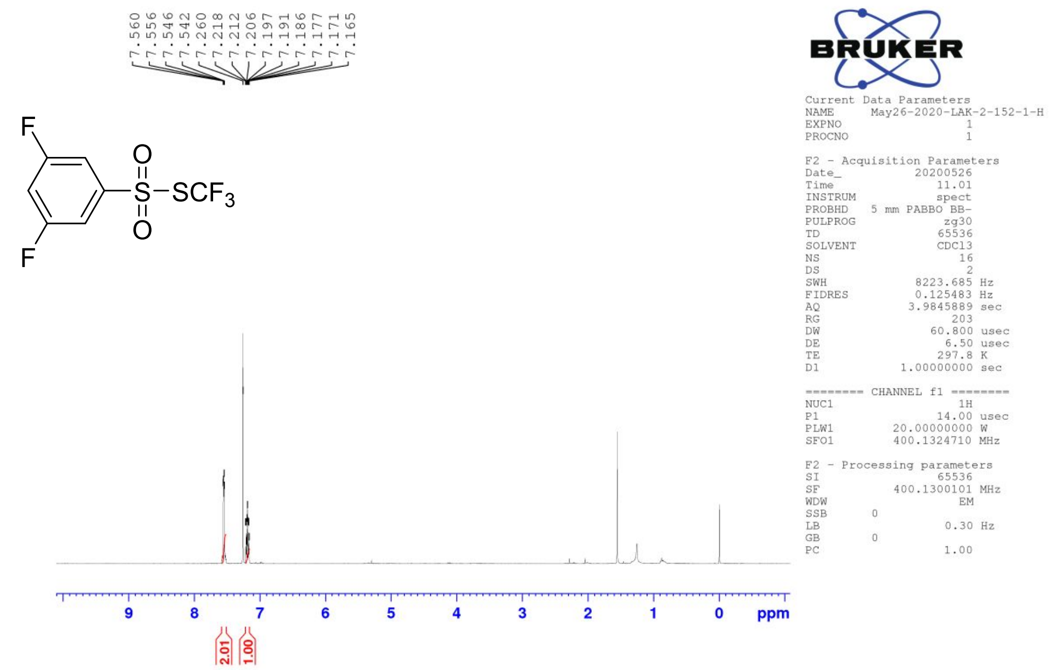

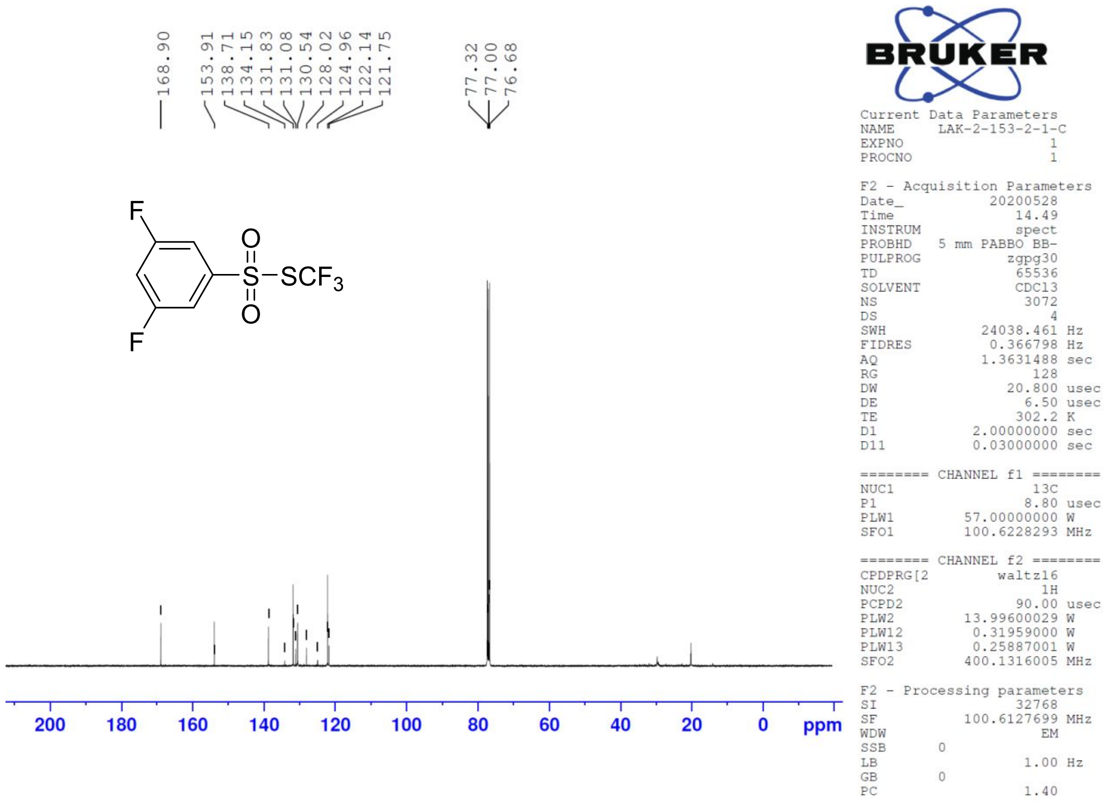

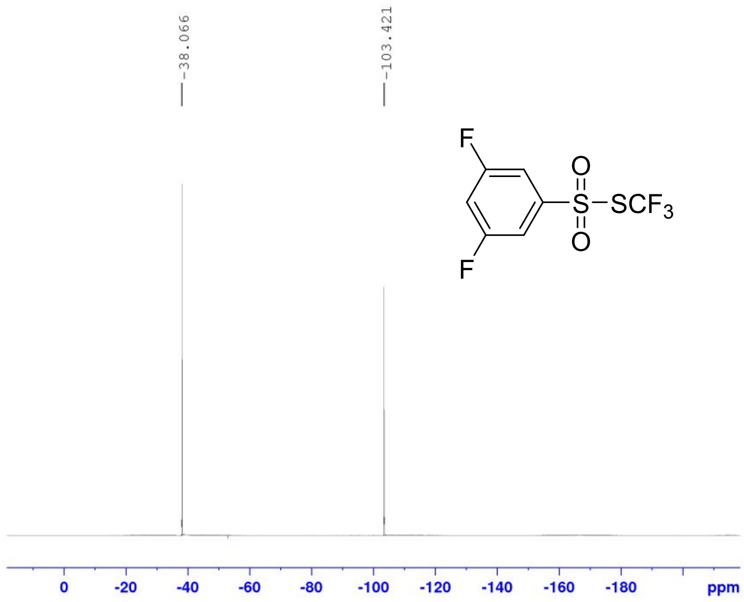


```
Current Data Parameters
NAME      May26-2020-LAK-2-152-1-F
EXPNO                    1
PROCNO                   1

F2 - Acquisition Parameters
Date_             20200526
Time                 11.04
INSTRUM              spect
PROBHD   5 mm PABBO BB-
PULPROG           zgfhigqn
TD                  131072
SOLVENT              CDCl3
NS                      16
DS                       4
SWH             89285.711 Hz
FIDRES           0.681196 Hz
AQ              0.7340032 sec
RG                     203
DW                   5.600 usec
DE                    6.50 usec
TE                   297.8 K
D1              1.00000000 sec
D11             0.03000000 sec
D12             0.00002000 sec

======== CHANNEL f1 ========
NUC1                   19F
P1                   13.20 usec
PLW1           18.19700050 W
SFO1           376.4607164 MHz

======== CHANNEL f2 ========
CPDPRG[2           waltz16
NUC2                    1H
PCPD2                90.00 usec
PLW2           13.99600029 W
PLW12           0.31959000 W
SFO2           400.1316005 MHz

F2 - Processing parameters
SI                   65536
SF             376.4983660 MHz
WDW                     EM
SSB      0
LB                    0.30 Hz
GB       0
PC                    1.00
```

^{1}H, ^{13}C and ^{19}F NMR spectra of ***[1,1'-biphenyl]-4-yl(trifluoromethyl)sulfane (3a)*** in $CDCl_3$

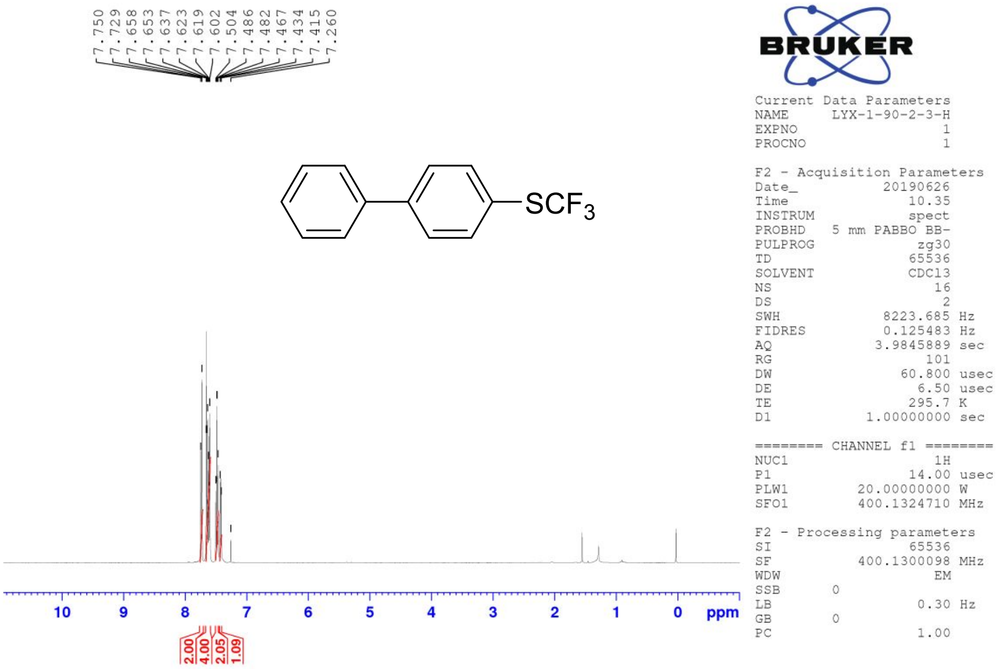

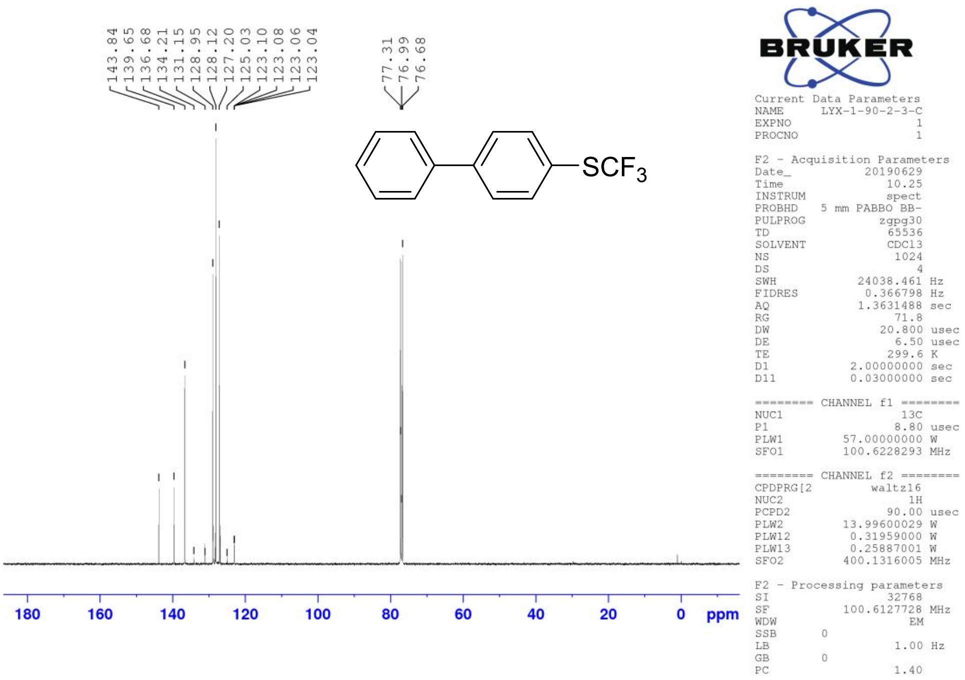

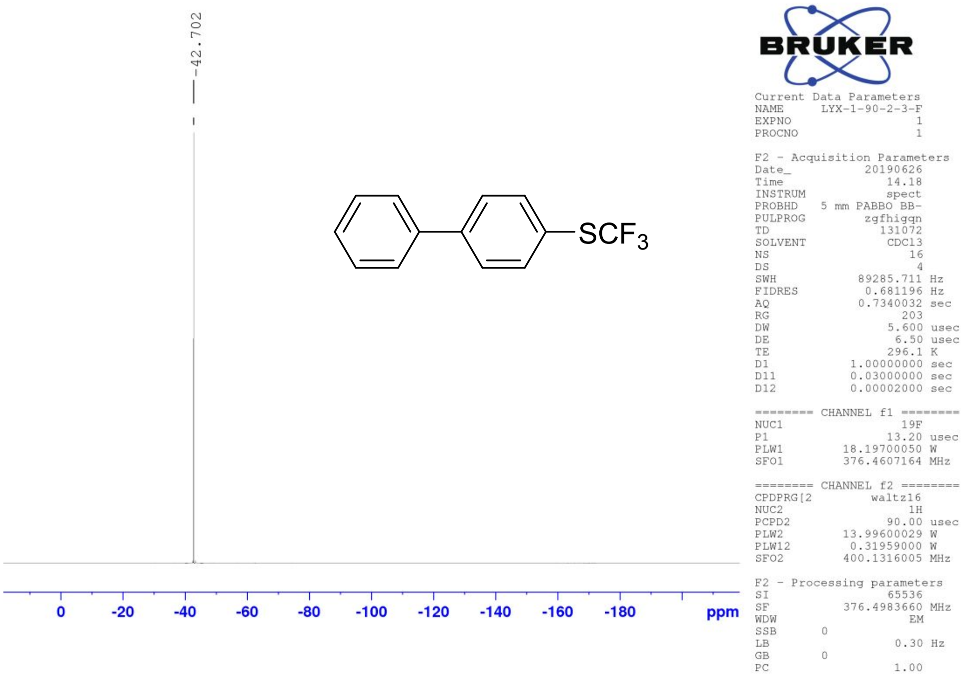

^{1}H, ^{13}C and ^{19}F NMR spectra of ***4-(4-((trifluoromethyl)thio)phenyl)morpholine (3b)*** in $CDCl_3$

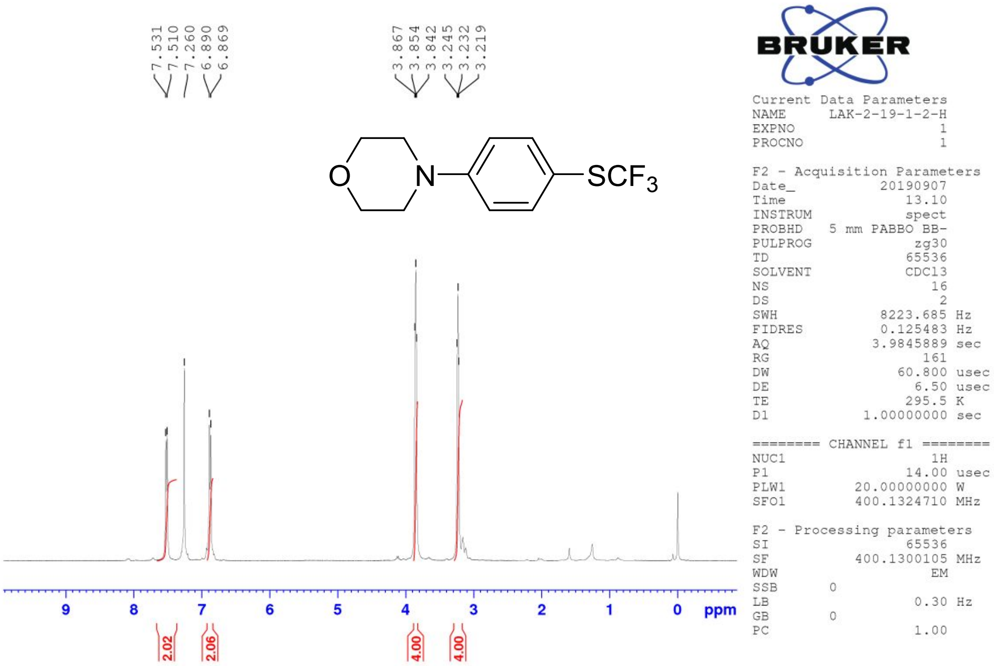

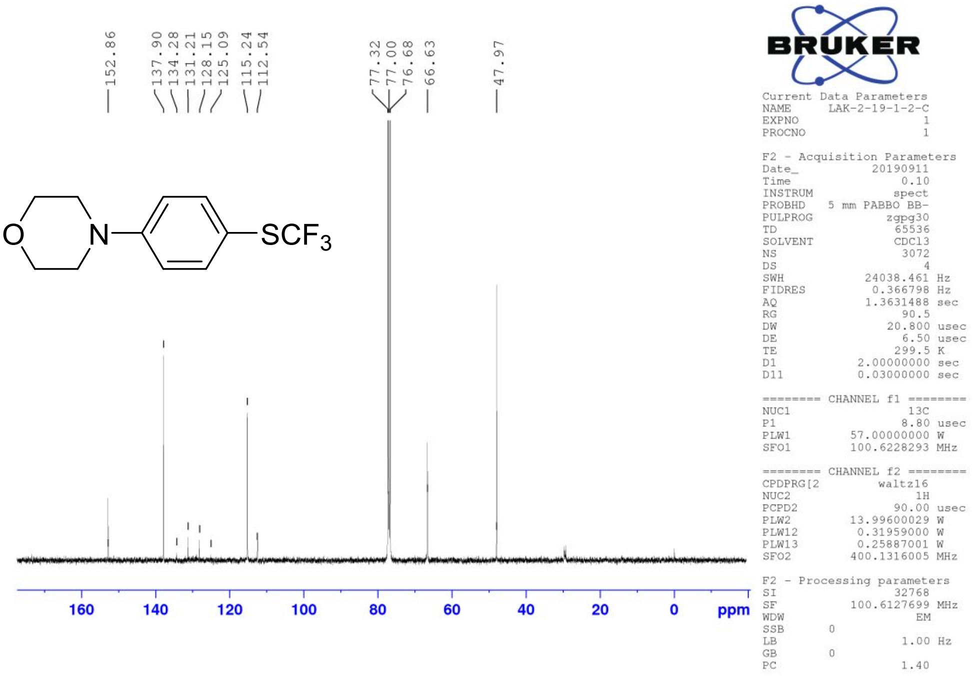
152.86
137.90
134.28
131.21
128.15
125.09
115.24
112.54
77.32
77.00
76.68
66.63
47.97
O
N
SCF3
160 140 120 100 80 60 40 20 0 ppm
BRUKER
Current Data Parameters
NAME LAK-2-19-1-2-C
EXPNO 1
PROCNO 1
F2 - Acquisition Parameters
Date_ 20190911
Time 0.10
INSTRUM spect
PROBHD 5 mm PABBO BB-
PULPROG zgpg30
TD 65536
SOLVENT CDCl3
NS 3072
DS 4
SWH 24038.461 Hz
FIDRES 0.366798 Hz
AQ 1.3631488 sec
RG 90.5
DW 20.800 usec
DE 6.50 usec
TE 299.5 K
D1 2.00000000 sec
D11 0.03000000 sec
======== CHANNEL f1 ========
NUC1 13C
P1 8.80 usec
PLW1 57.00000000 W
SFO1 100.6228293 MHz
======== CHANNEL f2 ========
CPDPRG[2 waltz16
NUC2 1H
PCPD2 90.00 usec
PLW2 13.99600029 W
PLW12 0.31959000 W
PLW13 0.25887001 W
SFO2 400.1316005 MHz
F2 - Processing parameters
SI 32768
SF 100.6127699 MHz
WDW EM
SSB 0
LB 1.00 Hz
GB 0
PC 1.40

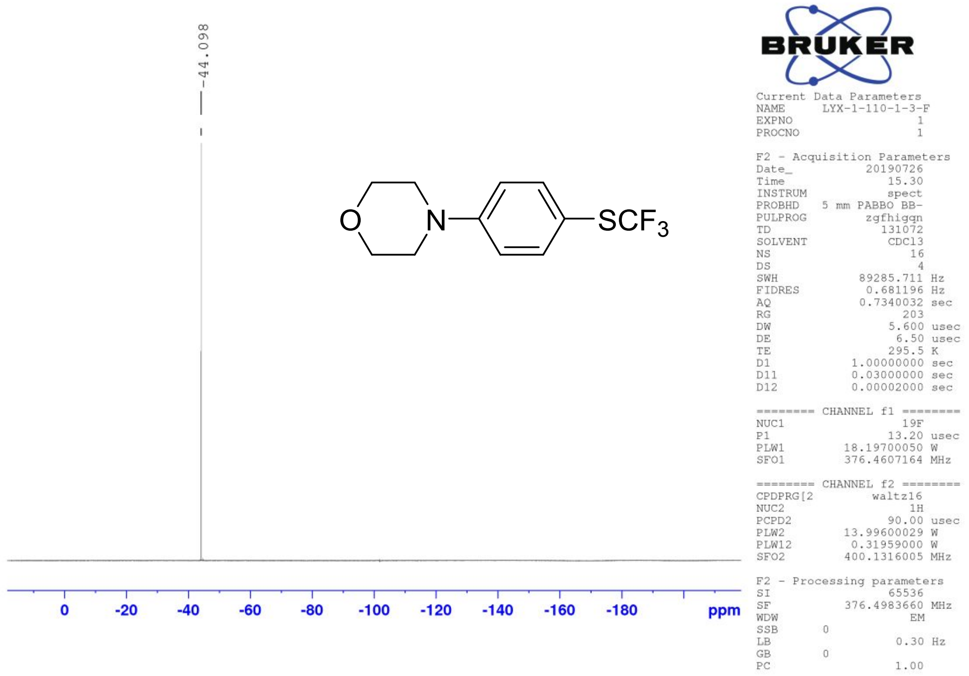
-44.098
O
N
SCF3
0 -20 -40 -60 -80 -100 -120 -140 -160 -180 ppm
BRUKER
Current Data Parameters
NAME LYX-1-110-1-3-F
EXPNO 1
PROCNO 1
F2 - Acquisition Parameters
Date_ 20190726
Time 15.30
INSTRUM spect
PROBHD 5 mm PABBO BB-
PULPROG zgfhigqn
TD 131072
SOLVENT CDCl3
NS 16
DS 4
SWH 89285.711 Hz
FIDRES 0.681196 Hz
AQ 0.7340032 sec
RG 203
DW 5.600 usec
DE 6.50 usec
TE 295.5 K
D1 1.00000000 sec
D11 0.03000000 sec
D12 0.00002000 sec
======== CHANNEL f1 ========
NUC1 19F
P1 13.20 usec
PLW1 18.19700050 W
SFO1 376.4607164 MHz
======== CHANNEL f2 ========
CPDPRG[2 waltz16
NUC2 1H
PCPD2 90.00 usec
PLW2 13.99600029 W
PLW12 0.31959000 W
SFO2 400.1316005 MHz
F2 - Processing parameters
SI 65536
SF 376.4983660 MHz
WDW EM
SSB 0
LB 0.30 Hz
GB 0
PC 1.00

^{1}H, ^{13}C and ^{19}F NMR spectra of ***(4-phenoxyphenyl)(trifluoromethyl)sulfane (3c)*** in $CDCl_3$

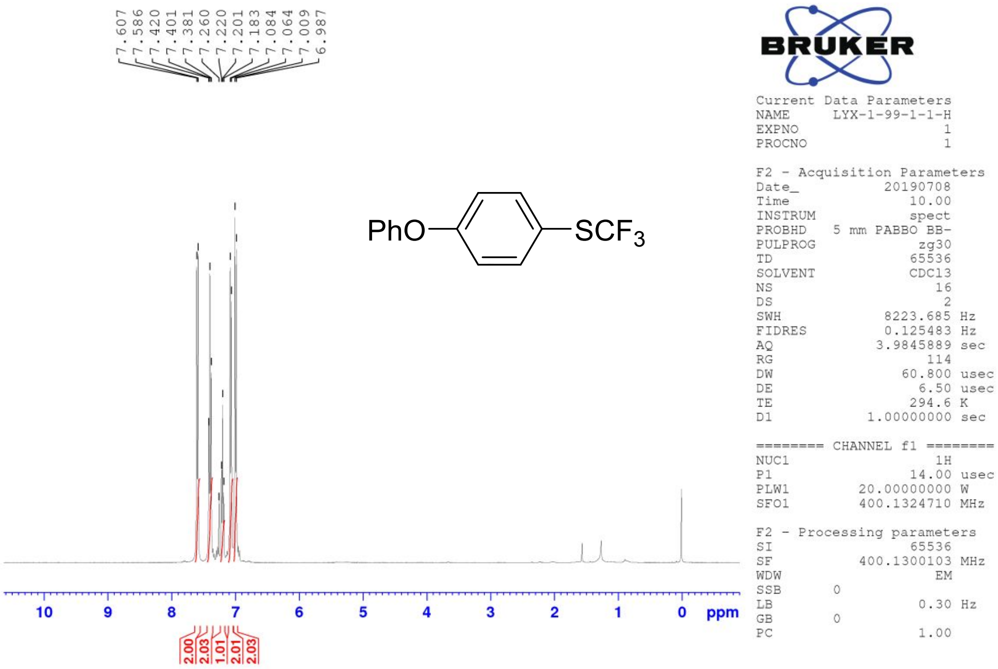

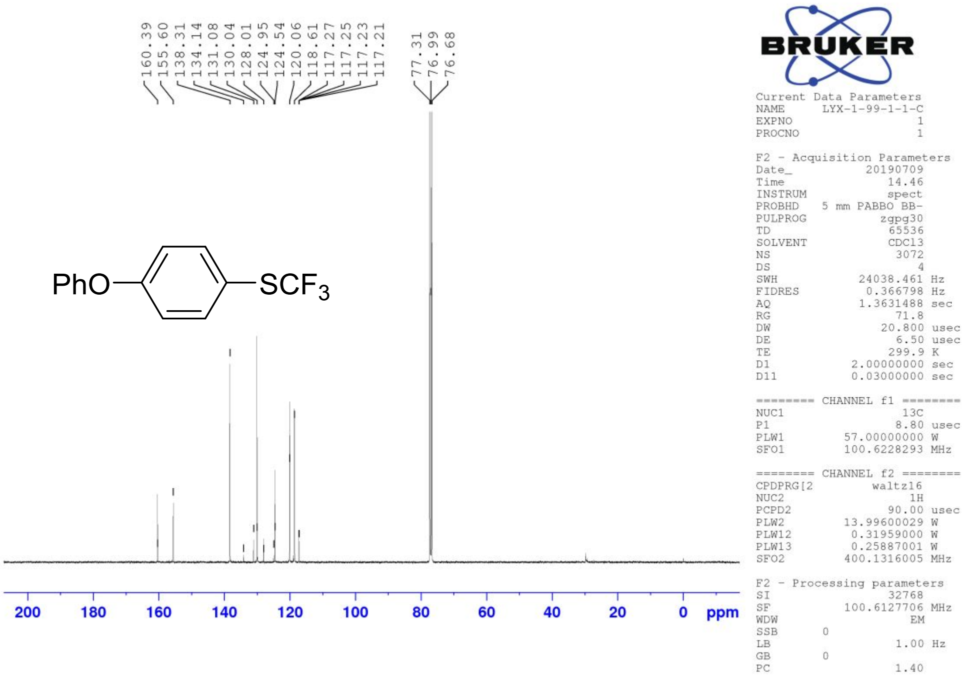

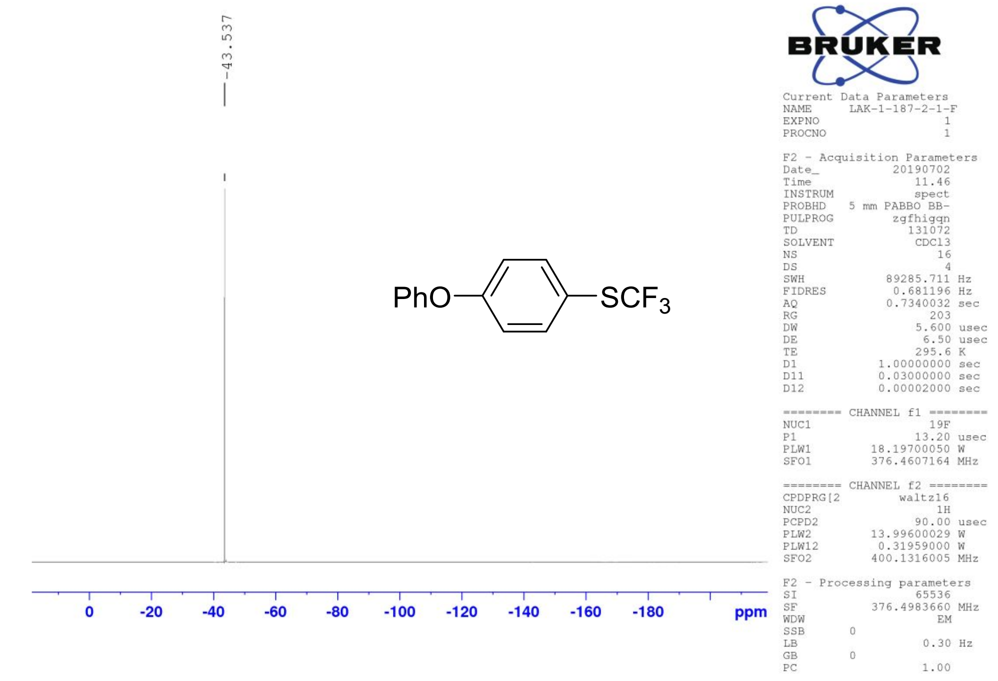

^{1}H, ^{13}C and ^{19}F NMR spectra of ***(4-(benzyloxy)phenyl)(trifluoromethyl)sulfane (3d)*** in $CDCl_3$

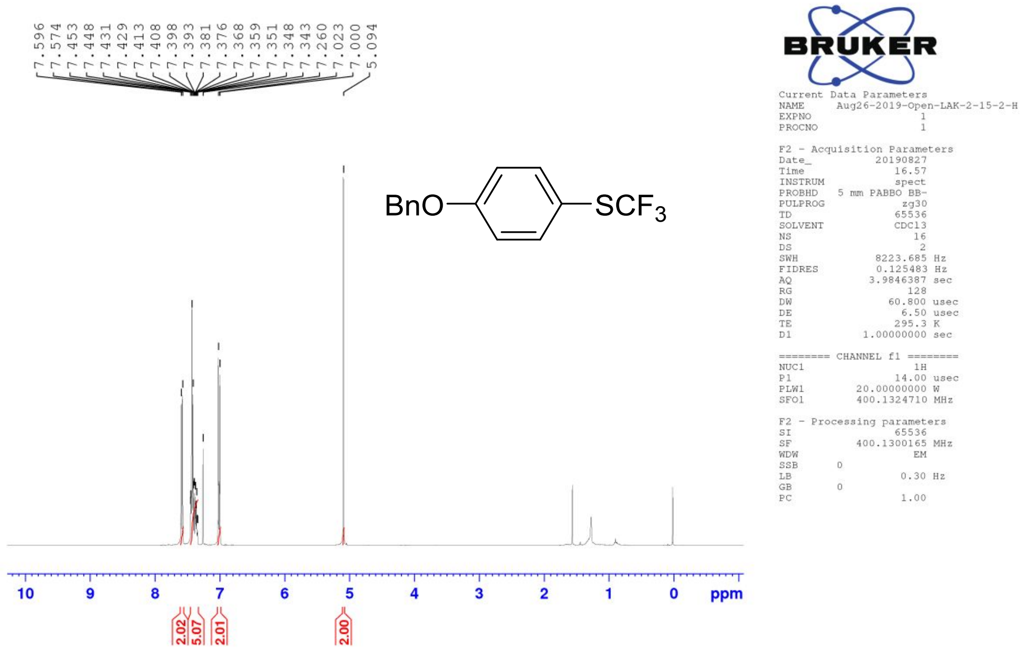

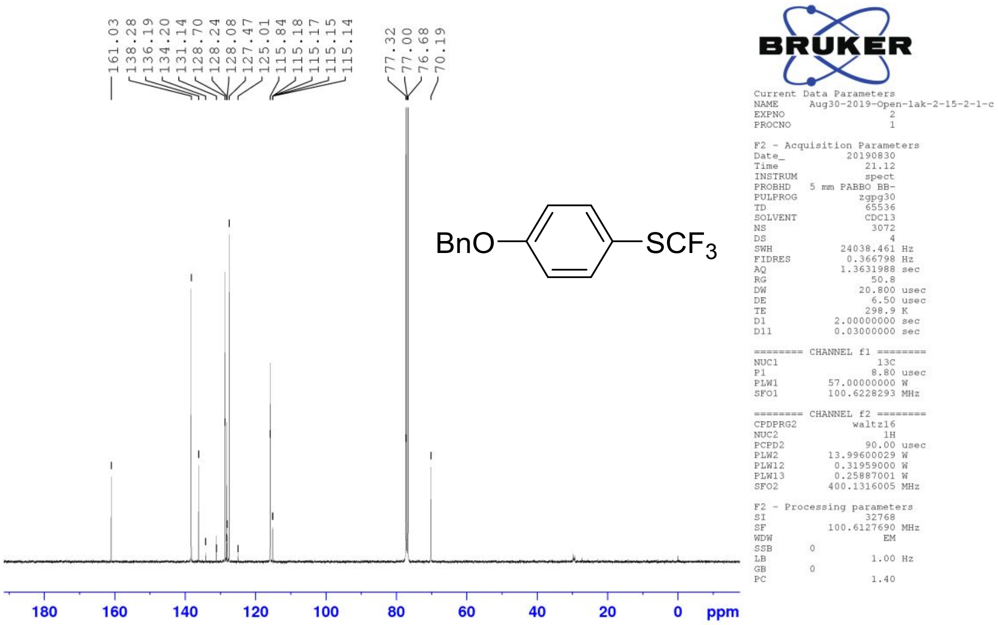
161.03
138.28
136.19
134.20
131.14
128.70
128.24
128.08
127.47
125.01
115.84
115.18
115.17
115.15
115.14
77.32
77.00
76.68
70.19
BnO
SCF3
180 160 140 120 100 80 60 40 20 0 ppm
BRUKER
Current Data Parameters
NAME Aug30-2019-Open-lak-2-15-2-1-c
EXPNO 2
PROCNO 1
F2 - Acquisition Parameters
Date_ 20190830
Time 21.12
INSTRUM spect
PROBHD 5 mm PABBO BB-
PULPROG zgpg30
TD 65536
SOLVENT CDCl3
NS 3072
DS 4
SWH 24038.461 Hz
FIDRES 0.366798 Hz
AQ 1.3631988 sec
RG 50.8
DW 20.800 usec
DE 6.50 usec
TE 298.9 K
D1 2.00000000 sec
D11 0.03000000 sec
======== CHANNEL f1 ========
NUC1 13C
P1 8.80 usec
PLW1 57.00000000 W
SFO1 100.6228293 MHz
======== CHANNEL f2 ========
CPDPRG2 waltz16
NUC2 1H
PCPD2 90.00 usec
PLW2 13.99600029 W
PLW12 0.31959000 W
PLW13 0.25887001 W
SFO2 400.1316005 MHz
F2 - Processing parameters
SI 32768
SF 100.6127690 MHz
WDW EM
SSB 0
LB 1.00 Hz
GB 0
PC 1.40

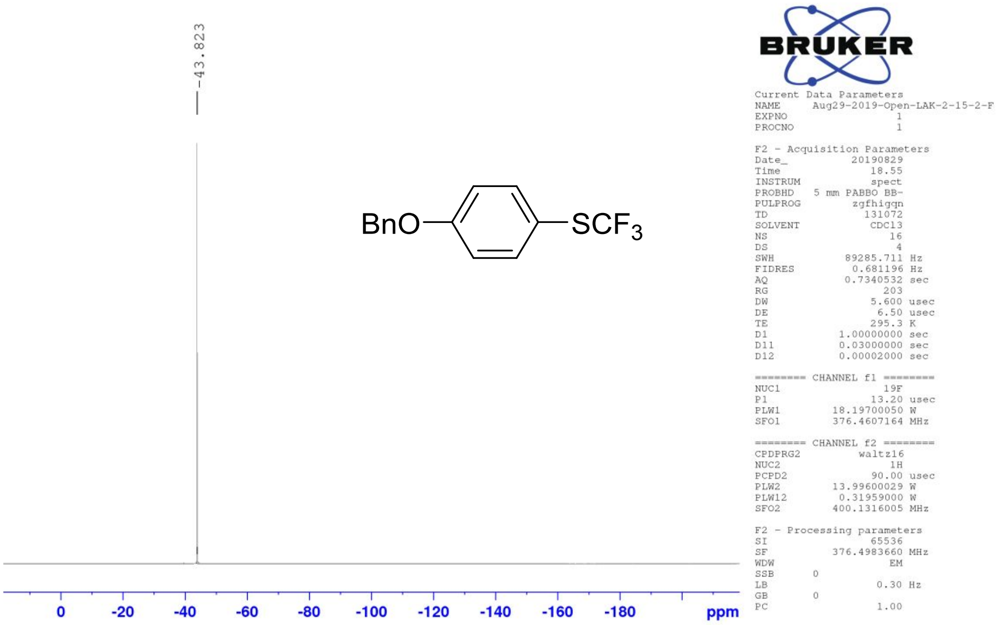
-43.823
BnO
SCF3
0 -20 -40 -60 -80 -100 -120 -140 -160 -180 ppm
BRUKER
Current Data Parameters
NAME Aug29-2019-Open-LAK-2-15-2-F
EXPNO 1
PROCNO 1
F2 - Acquisition Parameters
Date_ 20190829
Time 18.55
INSTRUM spect
PROBHD 5 mm PABBO BB-
PULPROG zgfhigqn
TD 131072
SOLVENT CDCl3
NS 16
DS 4
SWH 89285.711 Hz
FIDRES 0.681196 Hz
AQ 0.7340532 sec
RG 203
DW 5.600 usec
DE 6.50 usec
TE 295.3 K
D1 1.00000000 sec
D11 0.03000000 sec
D12 0.00002000 sec
======== CHANNEL f1 ========
NUC1 19F
P1 13.20 usec
PLW1 18.19700050 W
SFO1 376.4607164 MHz
======== CHANNEL f2 ========
CPDPRG2 waltz16
NUC2 1H
PCPD2 90.00 usec
PLW2 13.99600029 W
PLW12 0.31959000 W
SFO2 400.1316005 MHz
F2 - Processing parameters
SI 65536
SF 376.4983660 MHz
WDW EM
SSB 0
LB 0.30 Hz
GB 0
PC 1.00

^{1}H, ^{13}C and ^{19}F NMR spectra of ***methyl(4-((trifluoromethyl)thio)phenyl)sulfane (3e)*** in $CDCl_3$

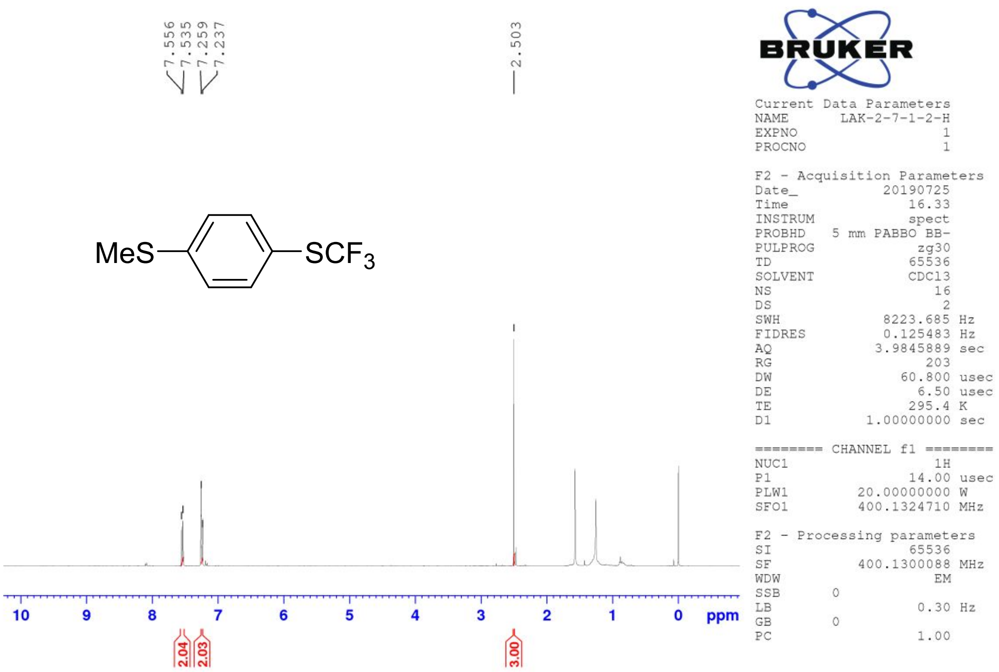

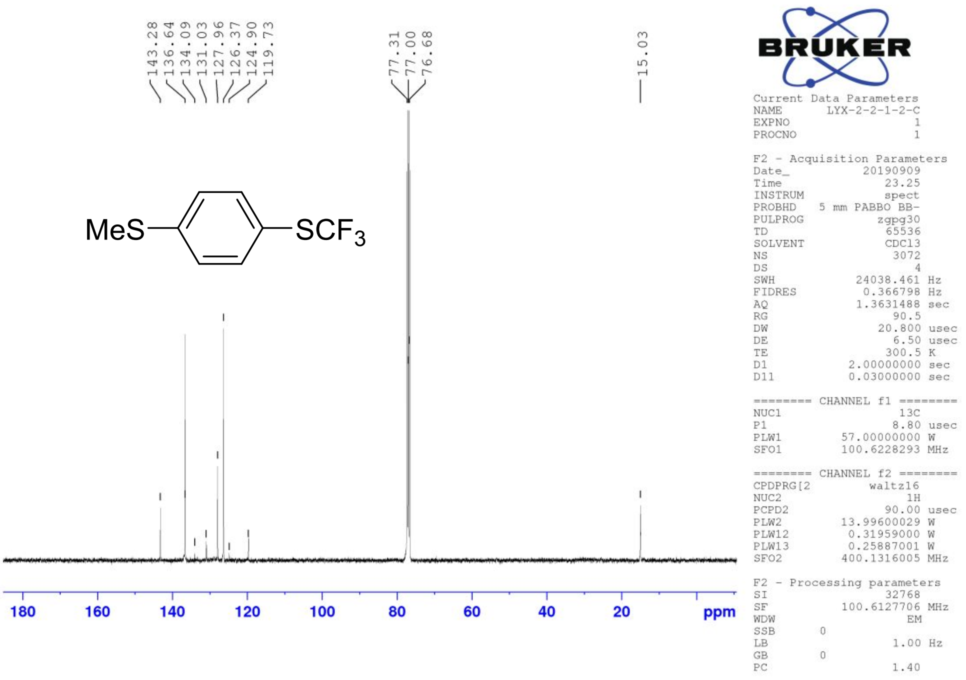

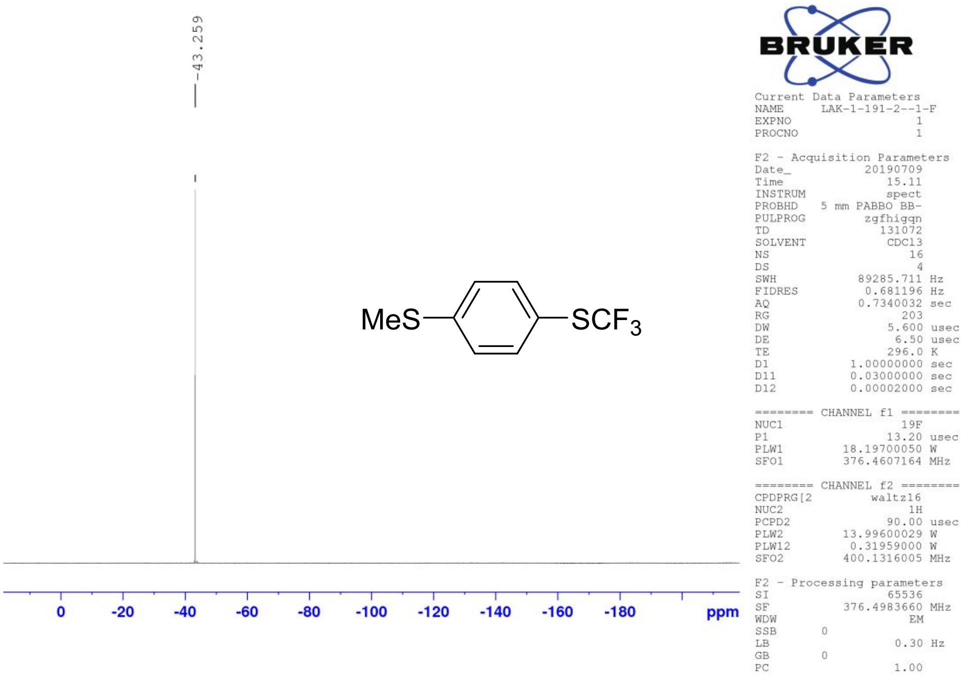

^{1}H, ^{13}C and ^{19}F NMR spectra of ***(4-(tert-butyl)phenyl)(trifluoromethyl)sulfane (3f)*** in $CDCl_3$

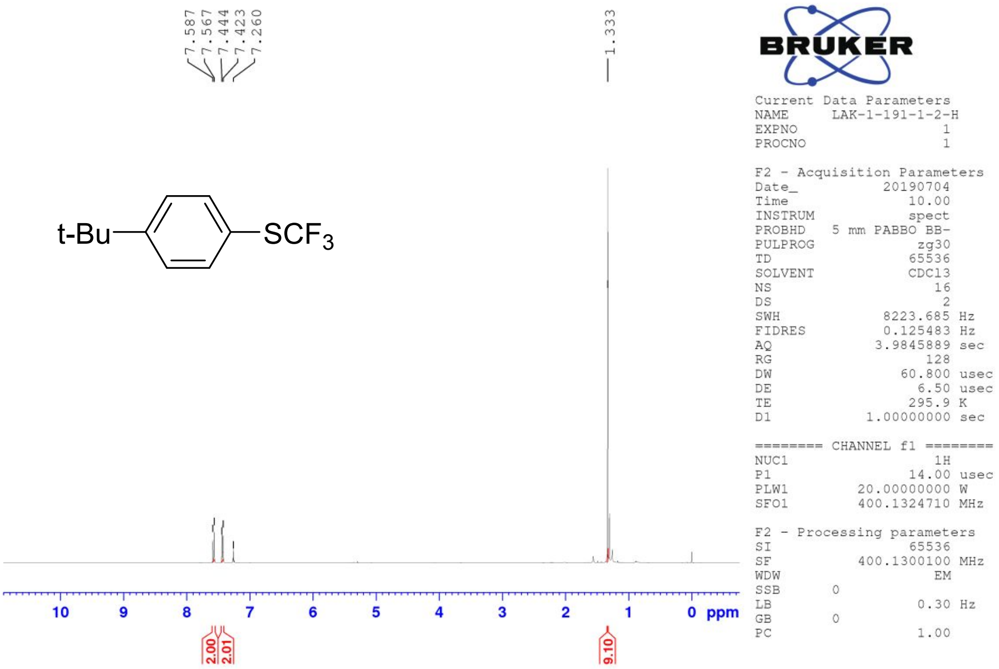

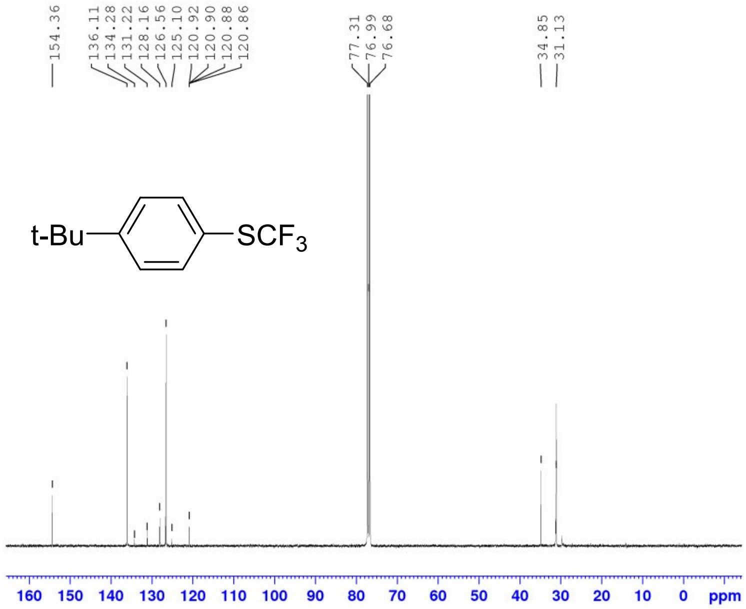

Current Data Parameters
NAME LYX-1-98-1-2--C
EXPNO 1
PROCNO 1

F2 - Acquisition Parameters
Date_ 20190719
Time 23.58
INSTRUM spect
PROBHD 5 mm PABBO BB-
PULPROG zgpg30
TD 65536
SOLVENT CDCl3
NS 3072
DS 4
SWH 24038.461 Hz
FIDRES 0.366798 Hz
AQ 1.3631488 sec
RG 80.6
DW 20.800 usec
DE 6.50 usec
TE 299.0 K
D1 2.00000000 sec
D11 0.03000000 sec

======== CHANNEL f1 ========
NUC1 13C
P1 8.80 usec
PLW1 57.00000000 W
SFO1 100.6228293 MHz

======== CHANNEL f2 ========
CPDPRG[2 waltz16
NUC2 1H
PCPD2 90.00 usec
PLW2 13.99600029 W
PLW12 0.31959000 W
PLW13 0.25887001 W
SFO2 400.1316005 MHz

F2 - Processing parameters
SI 32768
SF 100.6127699 MHz
WDW EM
SSB 0
LB 1.00 Hz
GB 0
PC 1.40

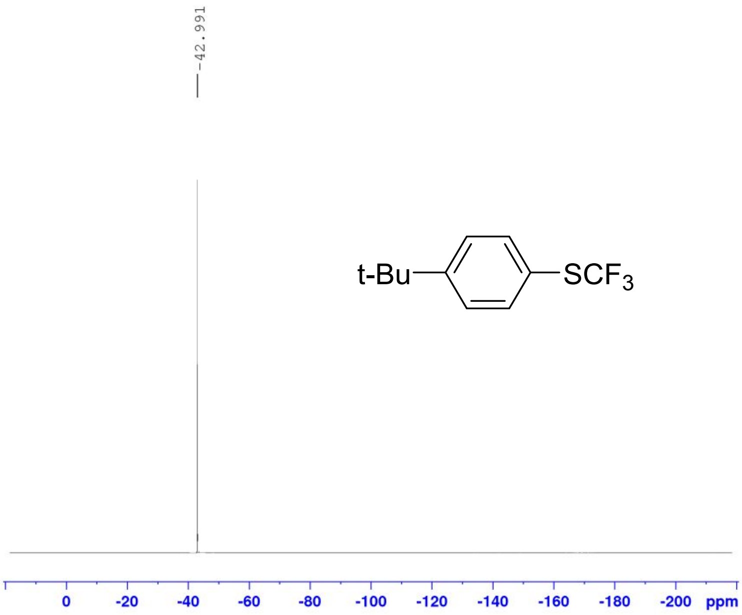

Current Data Parameters
NAME LYX-1-98-1-2-F
EXPNO 1
PROCNO 1

F2 - Acquisition Parameters
Date_ 20190715
Time 14.26
INSTRUM spect
PROBHD 5 mm PABBO BB-
PULPROG zgfhigqn
TD 131072
SOLVENT CDCl3
NS 16
DS 4
SWH 89285.711 Hz
FIDRES 0.681196 Hz
AQ 0.7340032 sec
RG 203
DW 5.600 usec
DE 6.50 usec
TE 296.3 K
D1 1.00000000 sec
D11 0.03000000 sec
D12 0.00002000 sec

======== CHANNEL f1 ========
NUC1 19F
P1 13.20 usec
PLW1 18.19700050 W
SFO1 376.4607164 MHz

======== CHANNEL f2 ========
CPDPRG[2 waltz16
NUC2 1H
PCPD2 90.00 usec
PLW2 13.99600029 W
PLW12 0.31959000 W
SFO2 400.1316005 MHz

F2 - Processing parameters
SI 65536
SF 376.4983660 MHz
WDW EM
SSB 0
LB 0.30 Hz
GB 0
PC 1.00

^{1}H, ^{13}C and ^{19}F NMR spectra of ***ethyl (E)-3-(4-((trifluoromethyl)thio)phenyl)acrylate (3g)*** in $CDCl_3$

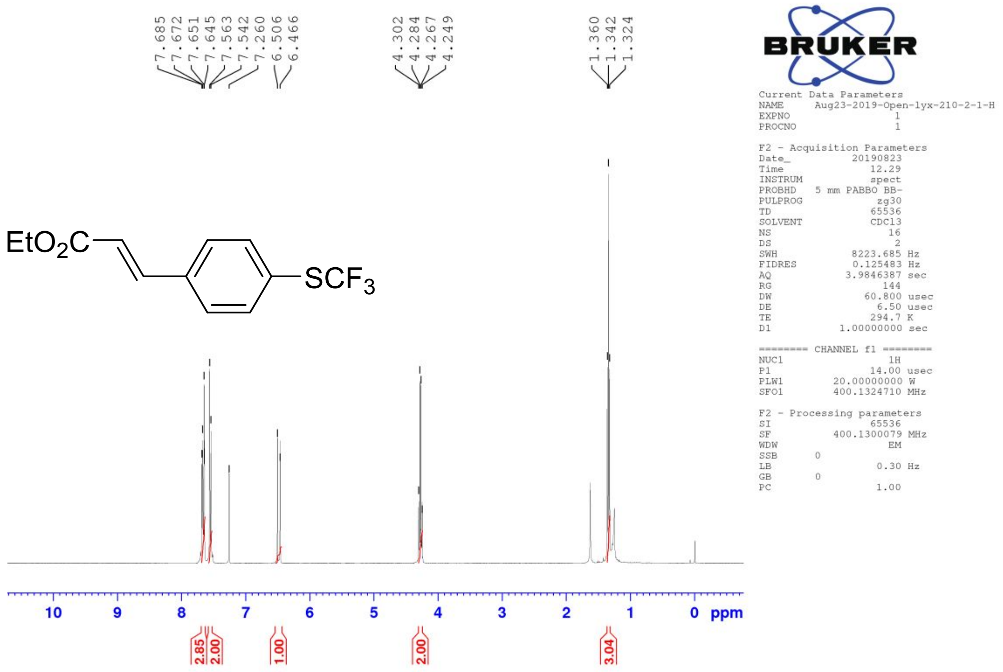

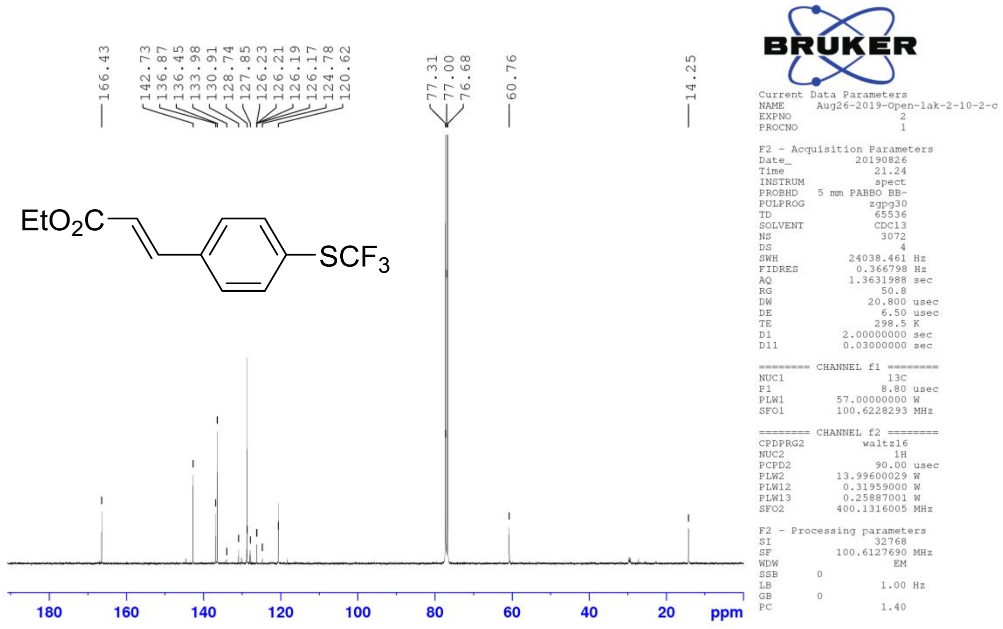

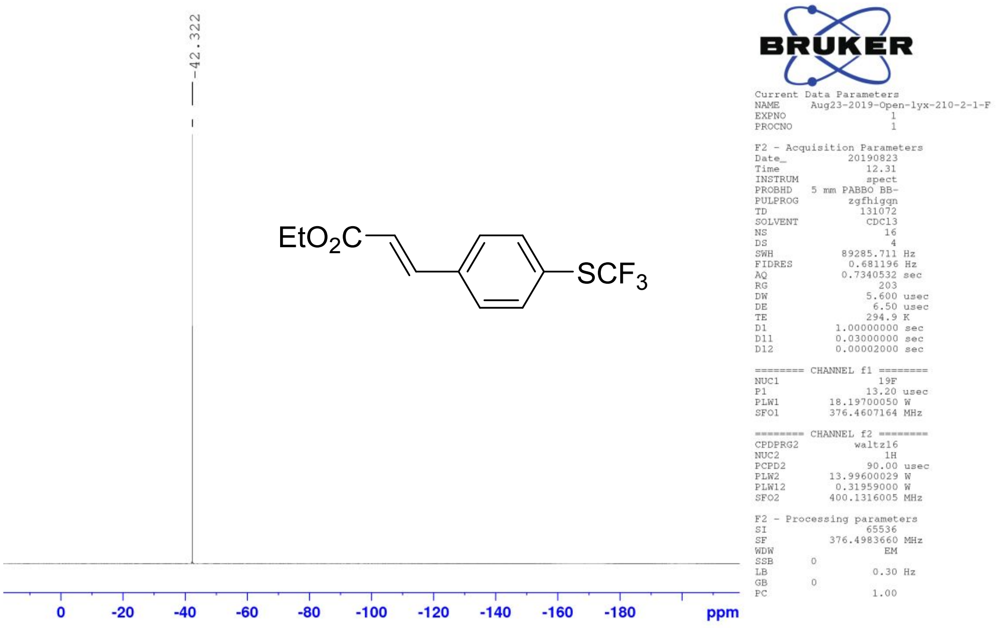

^{1}H, ^{13}C and ^{19}F NMR spectra of ***triisopropyl((4-((trifluoromethyl)thio)phenyl)ethynyl)silane (3h)*** in $CDCl_3$.

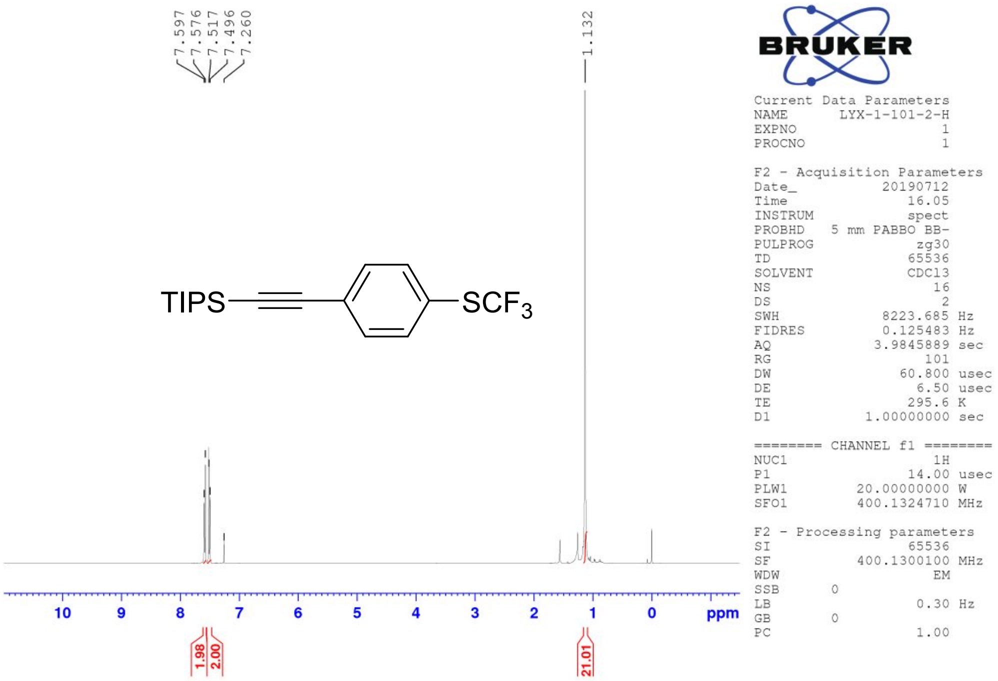

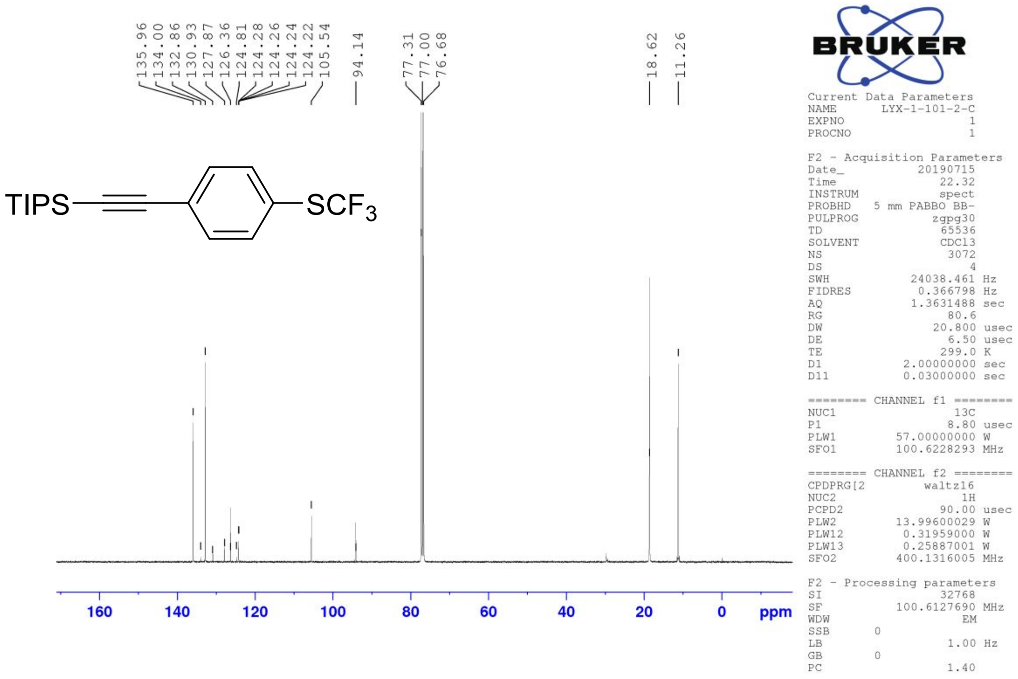
135.96
134.00
132.86
130.93
127.87
126.36
124.81
124.28
124.26
124.24
124.22
105.54
94.14
77.31
77.00
76.68
18.62
11.26
TIPS
SCF3
160 140 120 100 80 60 40 20 0 ppm
BRUKER
Current Data Parameters
NAME LYX-1-101-2-C
EXPNO 1
PROCNO 1
F2 - Acquisition Parameters
Date_ 20190715
Time 22.32
INSTRUM spect
PROBHD 5 mm PABBO BB-
PULPROG zgpg30
TD 65536
SOLVENT CDCl3
NS 3072
DS 4
SWH 24038.461 Hz
FIDRES 0.366798 Hz
AQ 1.3631488 sec
RG 80.6
DW 20.800 usec
DE 6.50 usec
TE 299.0 K
D1 2.00000000 sec
D11 0.03000000 sec
======== CHANNEL f1 ========
NUC1 13C
P1 8.80 usec
PLW1 57.00000000 W
SFO1 100.6228293 MHz
======== CHANNEL f2 ========
CPDPRG[2 waltz16
NUC2 1H
PCPD2 90.00 usec
PLW2 13.99600029 W
PLW12 0.31959000 W
PLW13 0.25887001 W
SFO2 400.1316005 MHz
F2 - Processing parameters
SI 32768
SF 100.6127690 MHz
WDW EM
SSB 0
LB 1.00 Hz
GB 0
PC 1.40

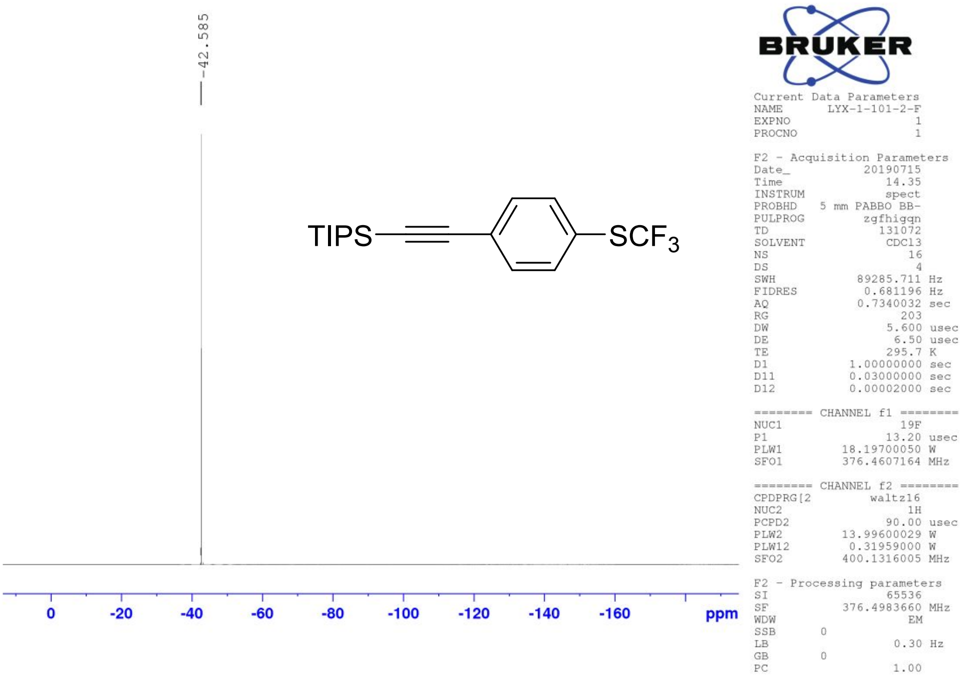
-42.585
TIPS
SCF3
0 -20 -40 -60 -80 -100 -120 -140 -160 ppm
BRUKER
Current Data Parameters
NAME LYX-1-101-2-F
EXPNO 1
PROCNO 1
F2 - Acquisition Parameters
Date_ 20190715
Time 14.35
INSTRUM spect
PROBHD 5 mm PABBO BB-
PULPROG zgfhigqn
TD 131072
SOLVENT CDCl3
NS 16
DS 4
SWH 89285.711 Hz
FIDRES 0.681196 Hz
AQ 0.7340032 sec
RG 203
DW 5.600 usec
DE 6.50 usec
TE 295.7 K
D1 1.00000000 sec
D11 0.03000000 sec
D12 0.00002000 sec
======== CHANNEL f1 ========
NUC1 19F
P1 13.20 usec
PLW1 18.19700050 W
SFO1 376.4607164 MHz
======== CHANNEL f2 ========
CPDPRG[2 waltz16
NUC2 1H
PCPD2 90.00 usec
PLW2 13.99600029 W
PLW12 0.31959000 W
SFO2 400.1316005 MHz
F2 - Processing parameters
SI 65536
SF 376.4983660 MHz
WDW EM
SSB 0
LB 0.30 Hz
GB 0
PC 1.00

^{1}H, ^{13}C and ^{19}F NMR spectra of ***(4-bromophenyl)(trifluoromethyl)sulfane (3i)*** in $CDCl_3$

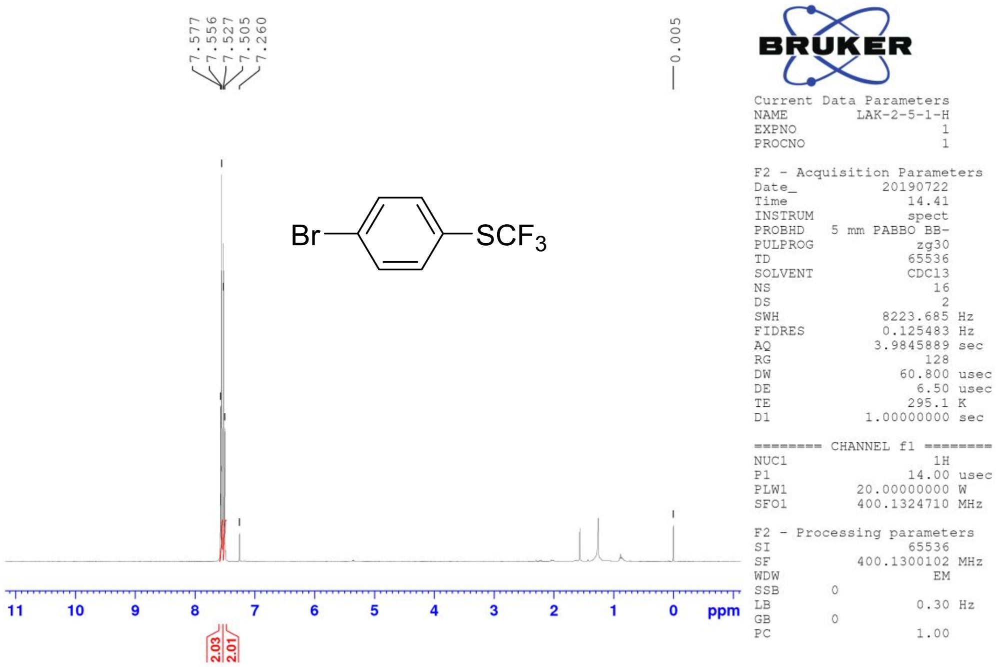

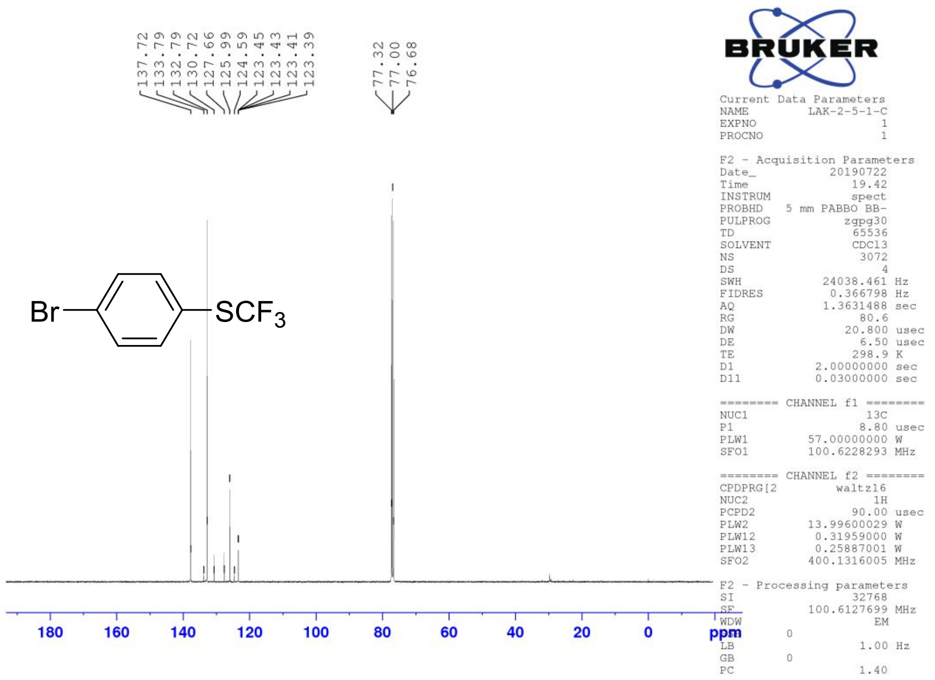

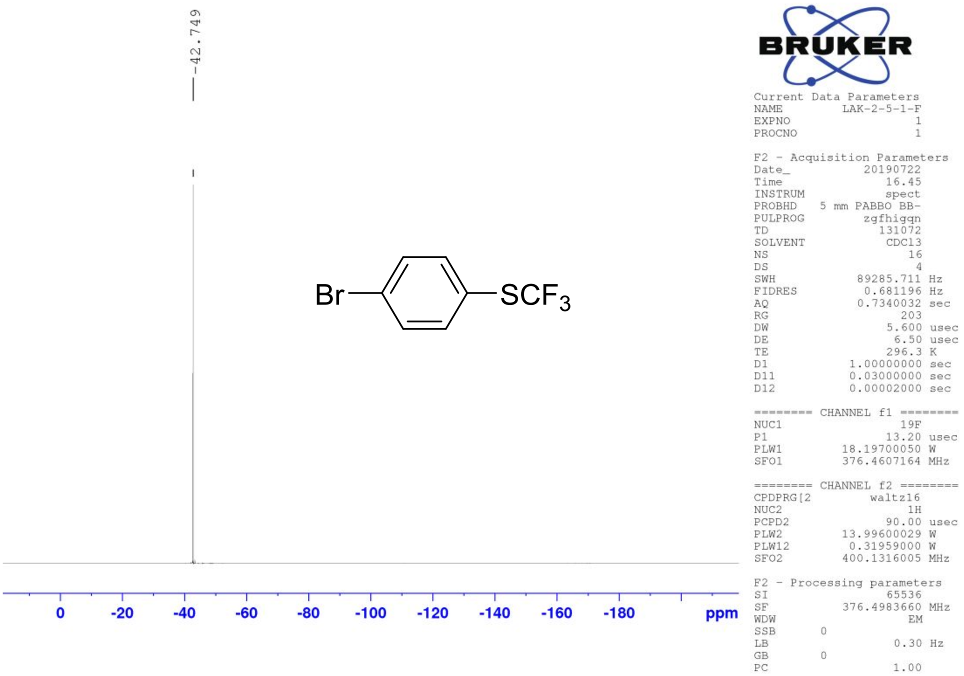

^{1}H, ^{13}C and ^{19}F NMR spectra of ***4-((trifluoromethyl)thio)benzonitrile (3j)*** in $CDCl_3$

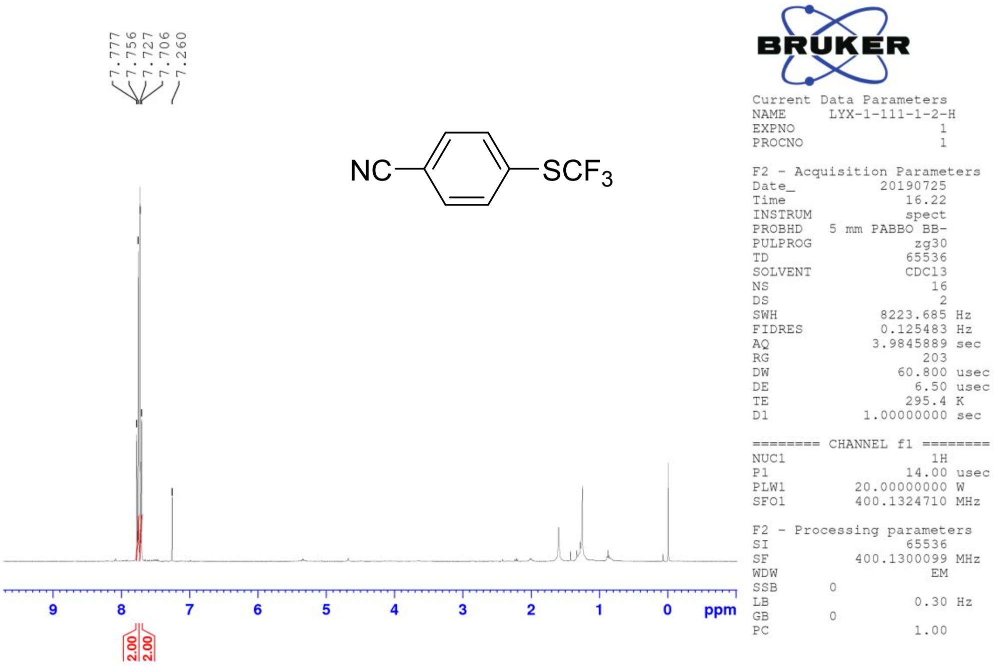

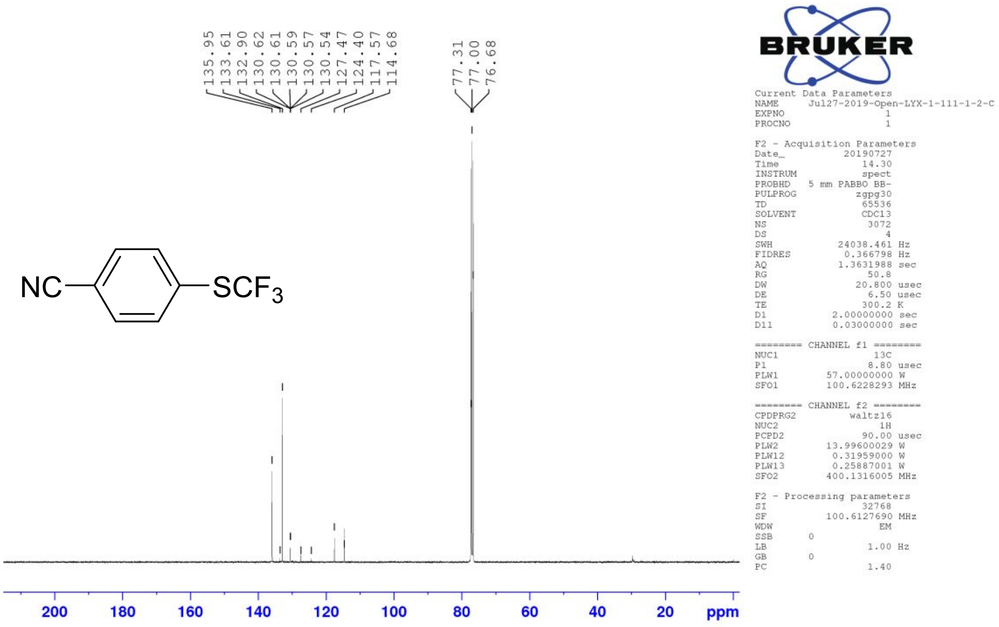

135.95
133.61
132.90
130.62
130.61
130.59
130.57
130.54
127.47
124.40
117.57
114.68
77.31
77.00
76.68
NC
SCF3
BRUKER
Current Data Parameters
NAME Jul27-2019-Open-LYX-1-111-1-2-C
EXPNO 1
PROCNO 1
F2 - Acquisition Parameters
Date_ 20190727
Time 14.30
INSTRUM spect
PROBHD 5 mm PABBO BB-
PULPROG zgpg30
TD 65536
SOLVENT CDCl3
NS 3072
DS 4
SWH 24038.461 Hz
FIDRES 0.366798 Hz
AQ 1.3631988 sec
RG 50.8
DW 20.800 usec
DE 6.50 usec
TE 300.2 K
D1 2.00000000 sec
D11 0.03000000 sec
======== CHANNEL f1 ========
NUC1 13C
P1 8.80 usec
PLW1 57.00000000 W
SFO1 100.6228293 MHz
======== CHANNEL f2 ========
CPDPRG2 waltz16
NUC2 1H
PCPD2 90.00 usec
PLW2 13.99600029 W
PLW12 0.31959000 W
PLW13 0.25887001 W
SFO2 400.1316005 MHz
F2 - Processing parameters
SI 32768
SF 100.6127690 MHz
WDW EM
SSB 0
LB 1.00 Hz
GB 0
PC 1.40
200 180 160 140 120 100 80 60 40 20 ppm

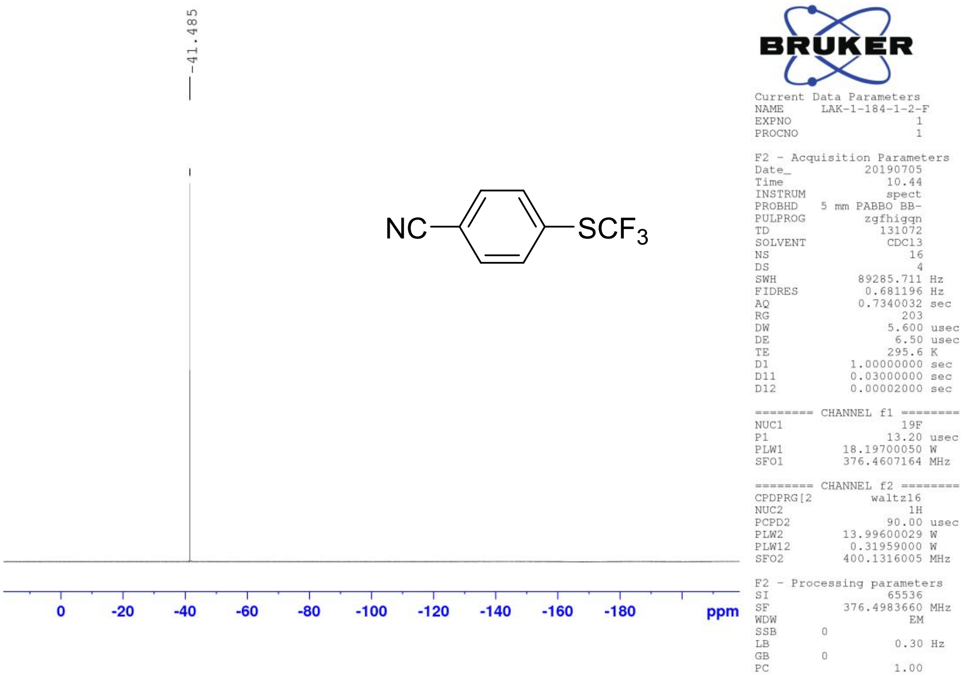

-41.485
NC
SCF3
BRUKER
Current Data Parameters
NAME LAK-1-184-1-2-F
EXPNO 1
PROCNO 1
F2 - Acquisition Parameters
Date_ 20190705
Time 10.44
INSTRUM spect
PROBHD 5 mm PABBO BB-
PULPROG zgfhigqn
TD 131072
SOLVENT CDCl3
NS 16
DS 4
SWH 89285.711 Hz
FIDRES 0.681196 Hz
AQ 0.7340032 sec
RG 203
DW 5.600 usec
DE 6.50 usec
TE 295.6 K
D1 1.00000000 sec
D11 0.03000000 sec
D12 0.00002000 sec
======== CHANNEL f1 ========
NUC1 19F
P1 13.20 usec
PLW1 18.19700050 W
SFO1 376.4607164 MHz
======== CHANNEL f2 ========
CPDPRG[2 waltz16
NUC2 1H
PCPD2 90.00 usec
PLW2 13.99600029 W
PLW12 0.31959000 W
SFO2 400.1316005 MHz
F2 - Processing parameters
SI 65536
SF 376.4983660 MHz
WDW EM
SSB 0
LB 0.30 Hz
GB 0
PC 1.00
0 -20 -40 -60 -80 -100 -120 -140 -160 -180 ppm

^{1}H, ^{13}C and ^{19}F NMR spectra of ***4-((trifluoromethyl)thio)benzoate (3k)*** in $CDCl_3$

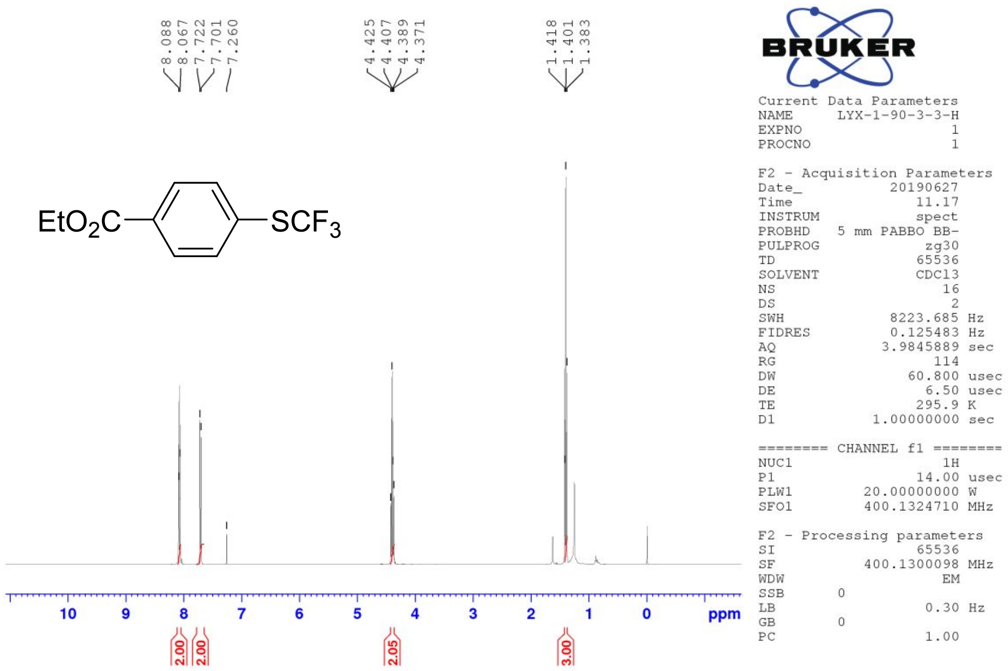

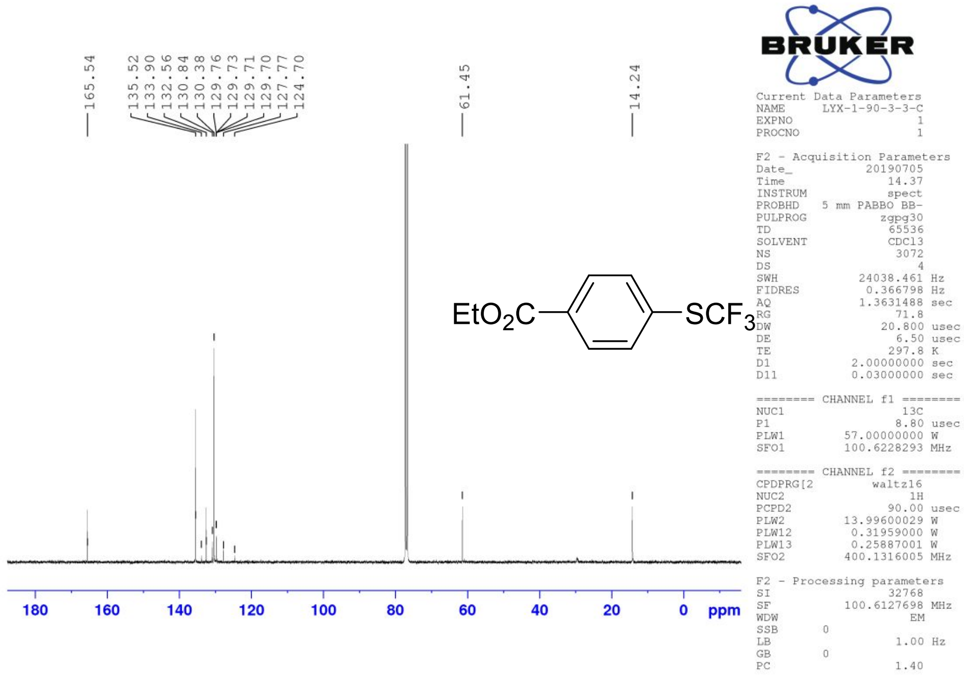

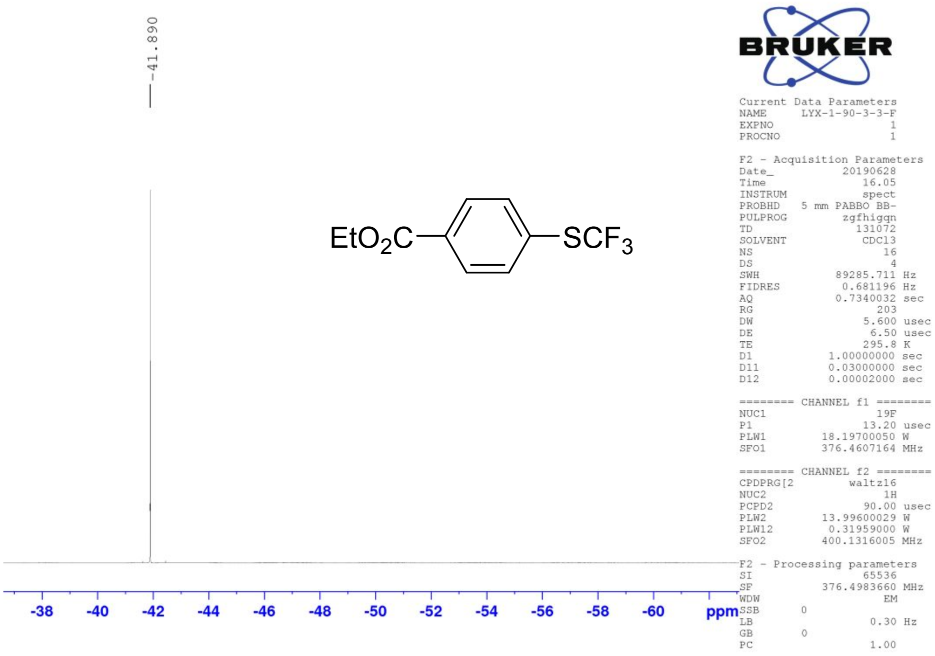

^1H, ^{13}C and ^{19}F NMR spectra of ***1-(4-((trifluoromethyl)thio)phenyl)ethanone (3l)*** in $CDCl_3$

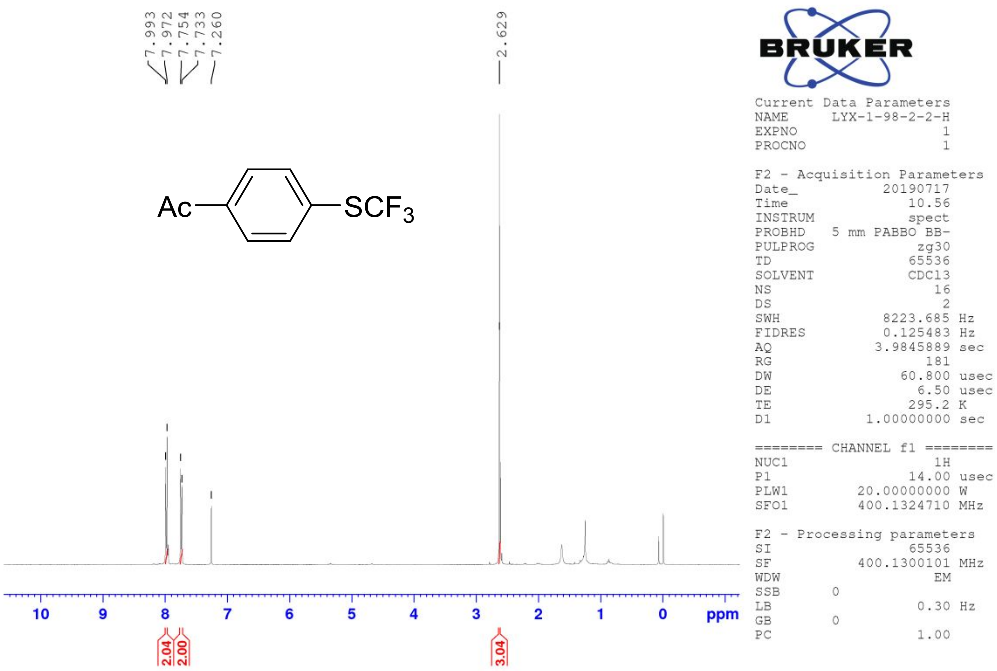

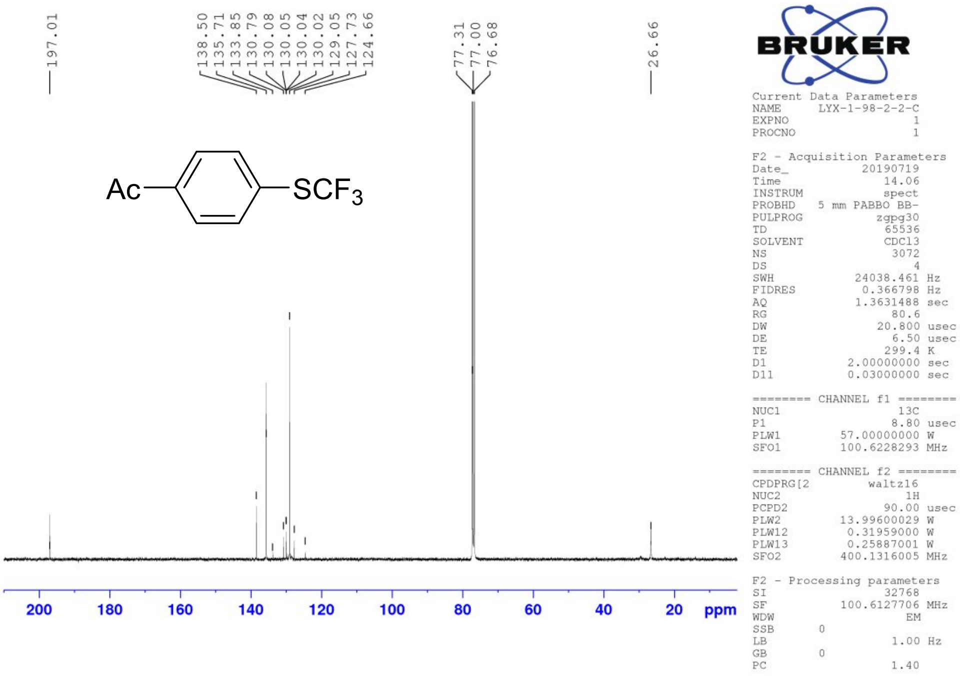
197.01
138.50
135.71
133.85
130.79
130.08
130.05
130.04
130.02
129.05
127.73
124.66
77.31
77.00
76.68
26.66
Ac
SCF3
200 180 160 140 120 100 80 60 40 20 ppm
BRUKER
Current Data Parameters
NAME LYX-1-98-2-2-C
EXPNO 1
PROCNO 1
F2 - Acquisition Parameters
Date_ 20190719
Time 14.06
INSTRUM spect
PROBHD 5 mm PABBO BB-
PULPROG zgpg30
TD 65536
SOLVENT CDCl3
NS 3072
DS 4
SWH 24038.461 Hz
FIDRES 0.366798 Hz
AQ 1.3631488 sec
RG 80.6
DW 20.800 usec
DE 6.50 usec
TE 299.4 K
D1 2.00000000 sec
D11 0.03000000 sec
======== CHANNEL f1 ========
NUC1 13C
P1 8.80 usec
PLW1 57.00000000 W
SFO1 100.6228293 MHz
======== CHANNEL f2 ========
CPDPRG[2 waltz16
NUC2 1H
PCPD2 90.00 usec
PLW2 13.99600029 W
PLW12 0.31959000 W
PLW13 0.25887001 W
SFO2 400.1316005 MHz
F2 - Processing parameters
SI 32768
SF 100.6127706 MHz
WDW EM
SSB 0
LB 1.00 Hz
GB 0
PC 1.40

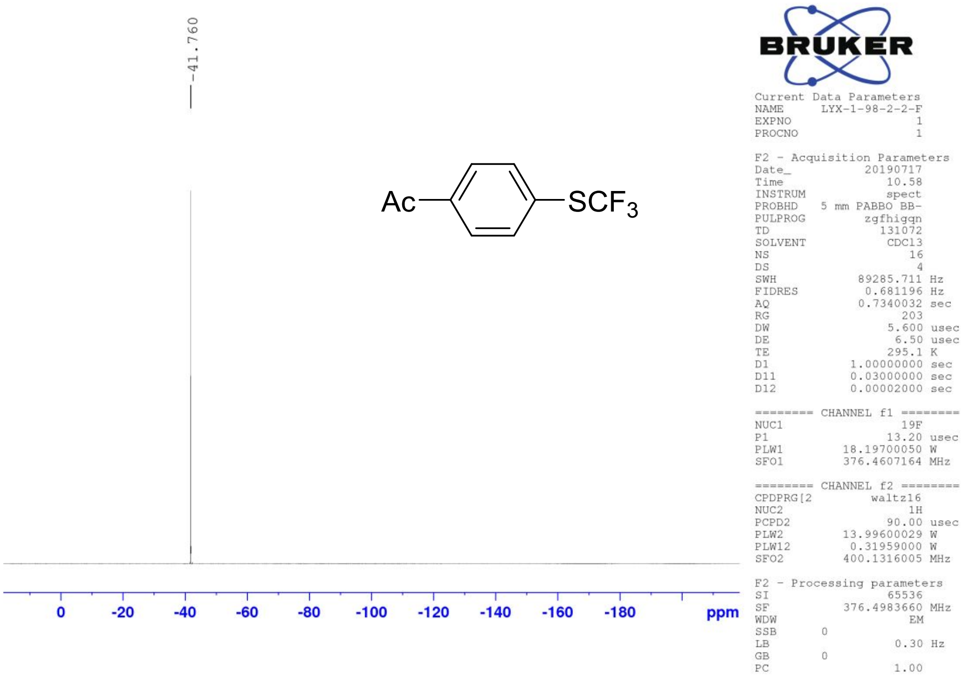
-41.760
Ac
SCF3
0 -20 -40 -60 -80 -100 -120 -140 -160 -180 ppm
BRUKER
Current Data Parameters
NAME LYX-1-98-2-2-F
EXPNO 1
PROCNO 1
F2 - Acquisition Parameters
Date_ 20190717
Time 10.58
INSTRUM spect
PROBHD 5 mm PABBO BB-
PULPROG zgfhigqn
TD 131072
SOLVENT CDCl3
NS 16
DS 4
SWH 89285.711 Hz
FIDRES 0.681196 Hz
AQ 0.7340032 sec
RG 203
DW 5.600 usec
DE 6.50 usec
TE 295.1 K
D1 1.00000000 sec
D11 0.03000000 sec
D12 0.00002000 sec
======== CHANNEL f1 ========
NUC1 19F
P1 13.20 usec
PLW1 18.19700050 W
SFO1 376.4607164 MHz
======== CHANNEL f2 ========
CPDPRG[2 waltz16
NUC2 1H
PCPD2 90.00 usec
PLW2 13.99600029 W
PLW12 0.31959000 W
SFO2 400.1316005 MHz
F2 - Processing parameters
SI 65536
SF 376.4983660 MHz
WDW EM
SSB 0
LB 0.30 Hz
GB 0
PC 1.00

^{1}H, ^{13}C and ^{19}F NMR spectra of ***phenyl(4-((trifluoromethyl)thio)phenyl)methanone (3m)*** in $CDCl_3$

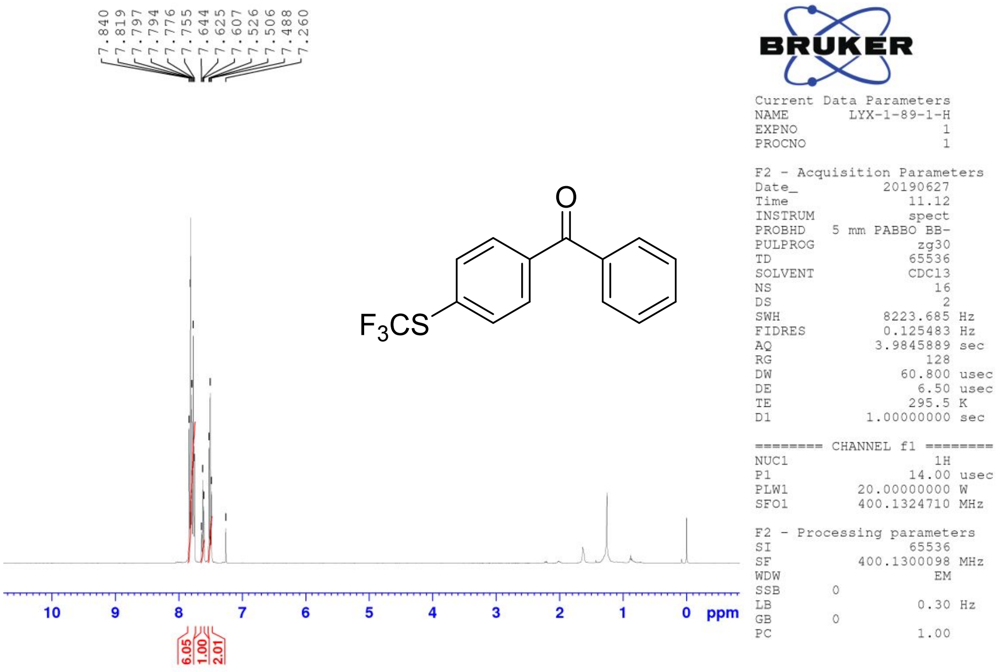

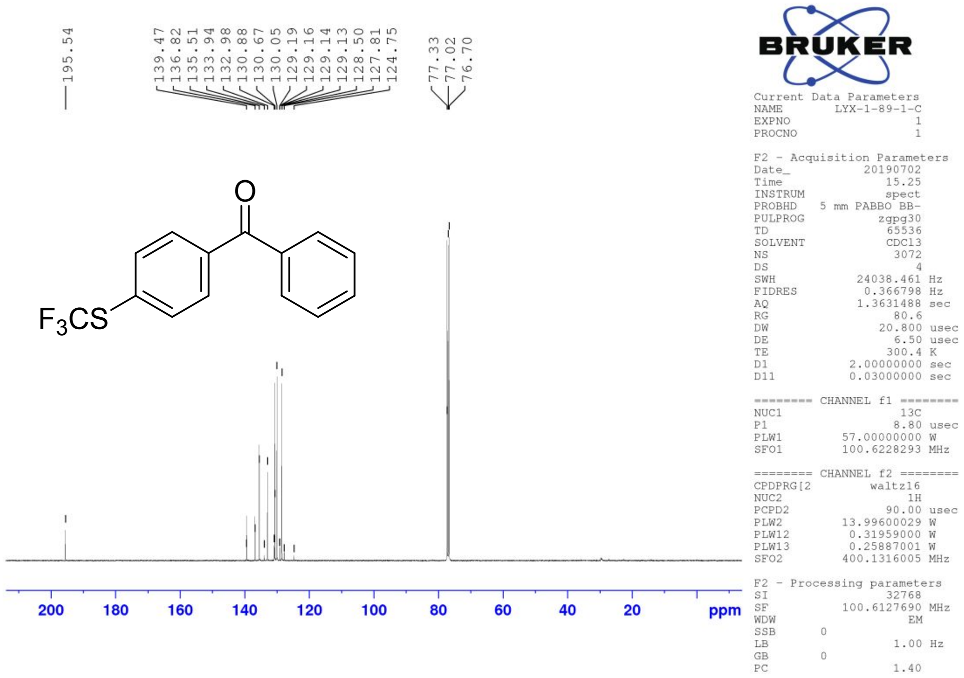

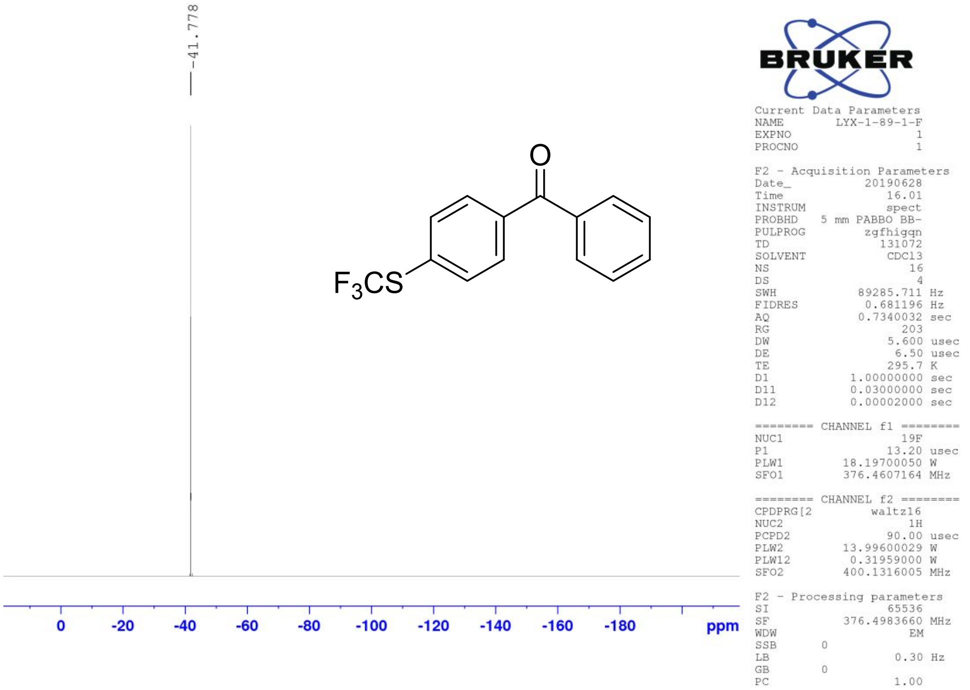

^1H, ^{13}C and ^{19}F NMR spectra of ***(3-bromophenyl)(trifluoromethyl)sulfane (3n)*** in $CDCl_3$

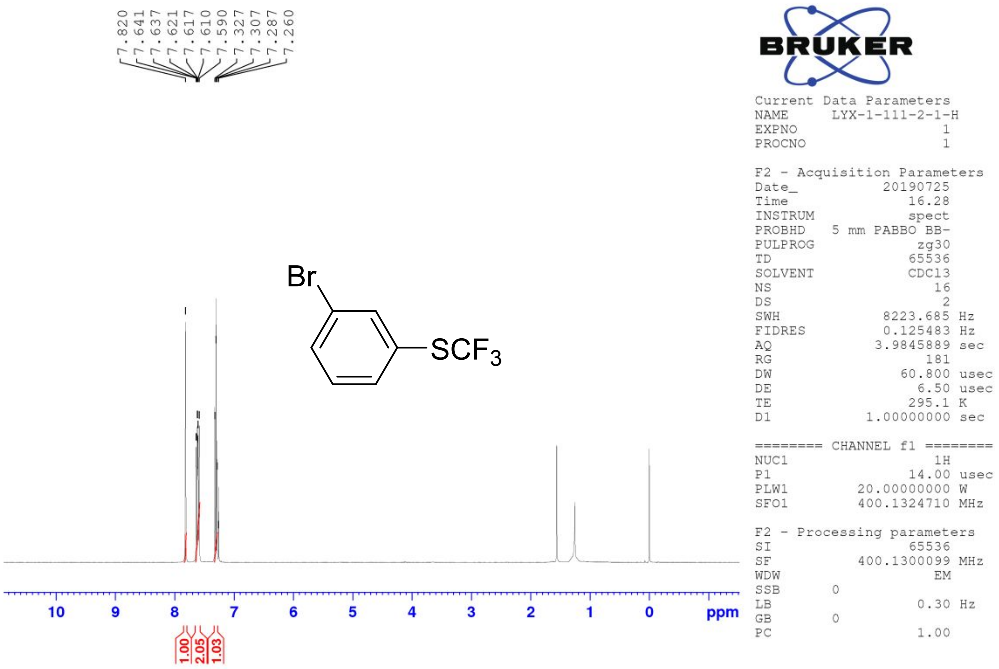

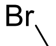

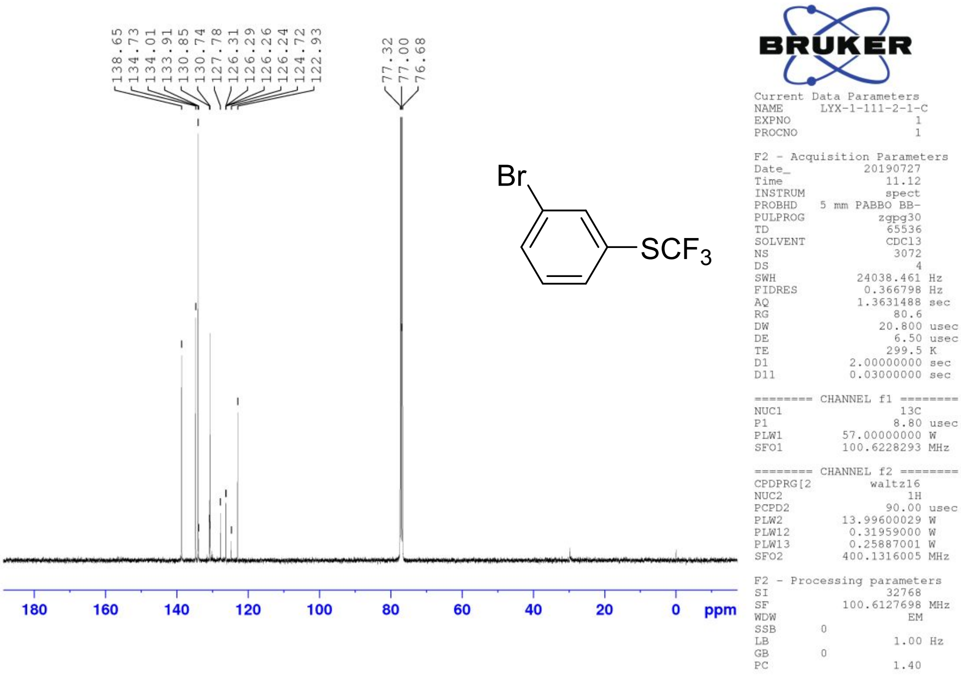
138.65
134.73
134.01
133.91
130.85
130.74
127.78
126.31
126.29
126.26
126.24
124.72
122.93
77.32
77.00
76.68
Br
SCF3
180 160 140 120 100 80 60 40 20 0 ppm
BRUKER
Current Data Parameters
NAME LYX-1-111-2-1-C
EXPNO 1
PROCNO 1
F2 - Acquisition Parameters
Date_ 20190727
Time 11.12
INSTRUM spect
PROBHD 5 mm PABBO BB-
PULPROG zgpg30
TD 65536
SOLVENT CDCl3
NS 3072
DS 4
SWH 24038.461 Hz
FIDRES 0.366798 Hz
AQ 1.3631488 sec
RG 80.6
DW 20.800 usec
DE 6.50 usec
TE 299.5 K
D1 2.00000000 sec
D11 0.03000000 sec
======== CHANNEL f1 ========
NUC1 13C
P1 8.80 usec
PLW1 57.00000000 W
SFO1 100.6228293 MHz
======== CHANNEL f2 ========
CPDPRG[2 waltz16
NUC2 1H
PCPD2 90.00 usec
PLW2 13.99600029 W
PLW12 0.31959000 W
PLW13 0.25887001 W
SFO2 400.1316005 MHz
F2 - Processing parameters
SI 32768
SF 100.6127698 MHz
WDW EM
SSB 0
LB 1.00 Hz
GB 0
PC 1.40

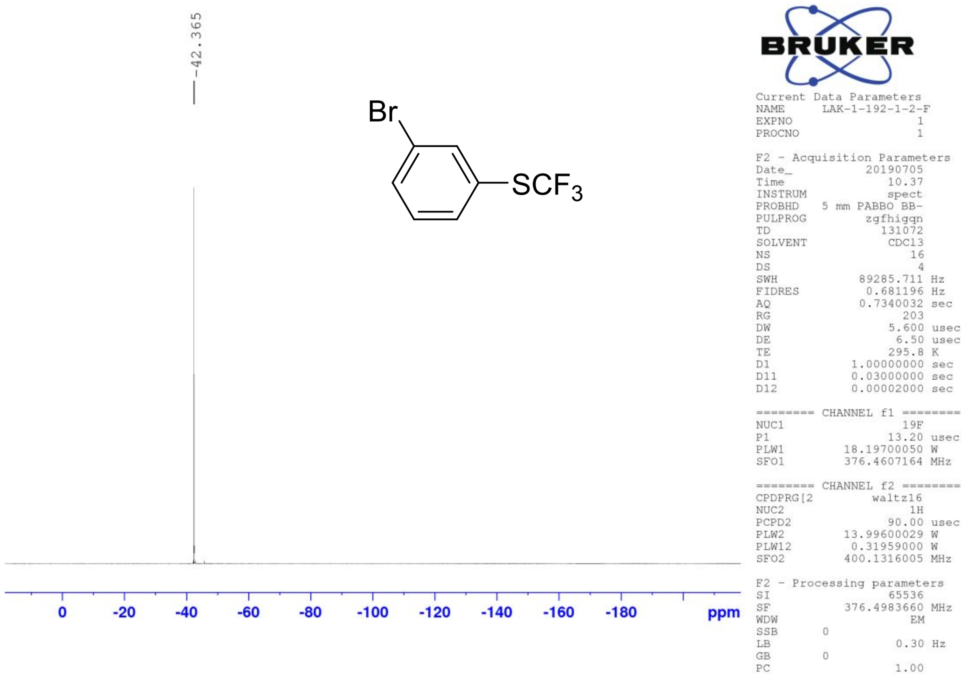
-42.365
Br
SCF3
0 -20 -40 -60 -80 -100 -120 -140 -160 -180 ppm
BRUKER
Current Data Parameters
NAME LAK-1-192-1-2-F
EXPNO 1
PROCNO 1
F2 - Acquisition Parameters
Date_ 20190705
Time 10.37
INSTRUM spect
PROBHD 5 mm PABBO BB-
PULPROG zgfhigqn
TD 131072
SOLVENT CDCl3
NS 16
DS 4
SWH 89285.711 Hz
FIDRES 0.681196 Hz
AQ 0.7340032 sec
RG 203
DW 5.600 usec
DE 6.50 usec
TE 295.8 K
D1 1.00000000 sec
D11 0.03000000 sec
D12 0.00002000 sec
======== CHANNEL f1 ========
NUC1 19F
P1 13.20 usec
PLW1 18.19700050 W
SFO1 376.4607164 MHz
======== CHANNEL f2 ========
CPDPRG[2 waltz16
NUC2 1H
PCPD2 90.00 usec
PLW2 13.99600029 W
PLW12 0.31959000 W
SFO2 400.1316005 MHz
F2 - Processing parameters
SI 65536
SF 376.4983660 MHz
WDW EM
SSB 0
LB 0.30 Hz
GB 0
PC 1.00

^{1}H, ^{13}C and ^{19}F NMR spectra of ***(trifluoromethyl)(3,4,5-trimethoxyphenyl)sulfane (3o)*** in $CDCl_3$

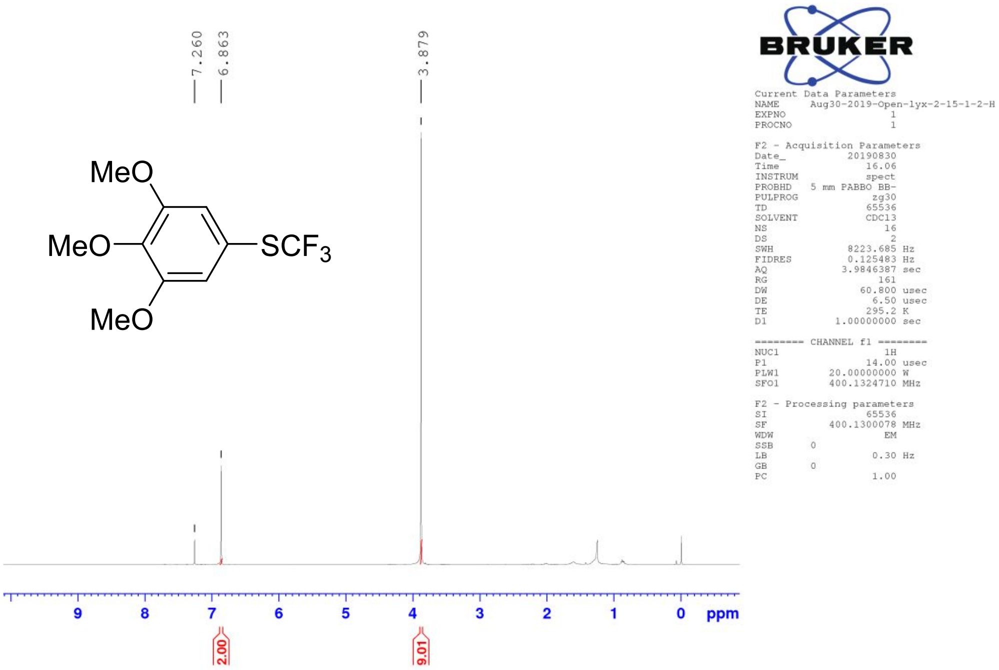

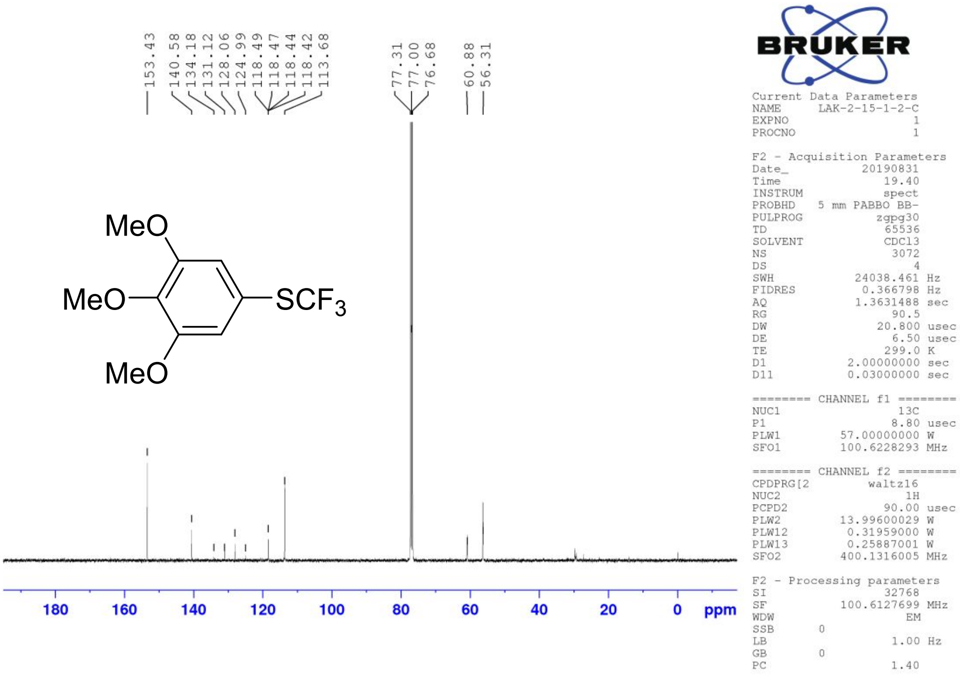

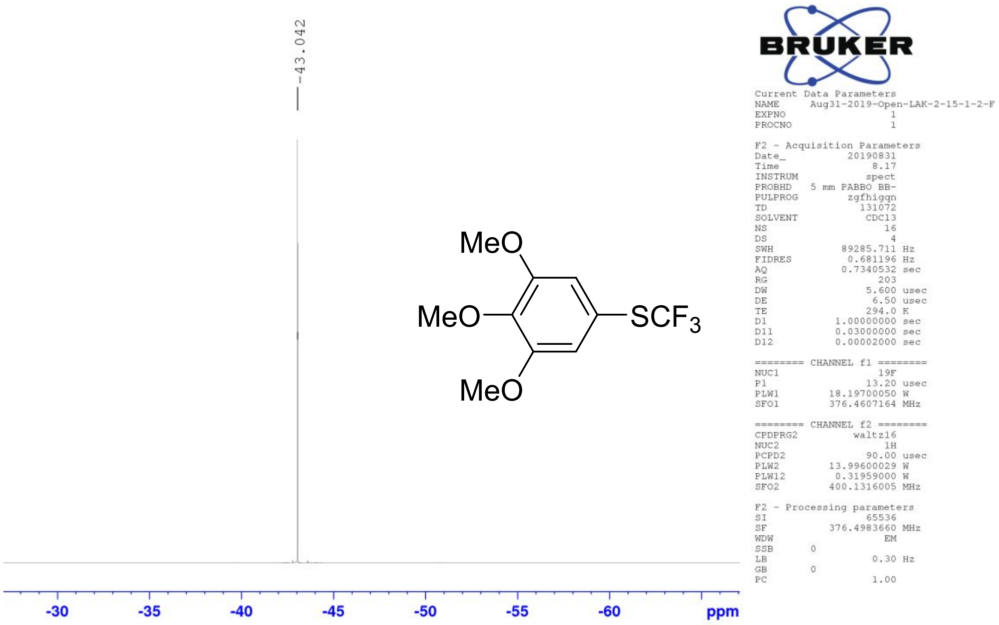

^1H, ^{13}C and ^{19}F NMR spectra of ***5-((trifluoromethyl)thio)benzo[d][1,3]dioxole (3p)*** in $CDCl_3$

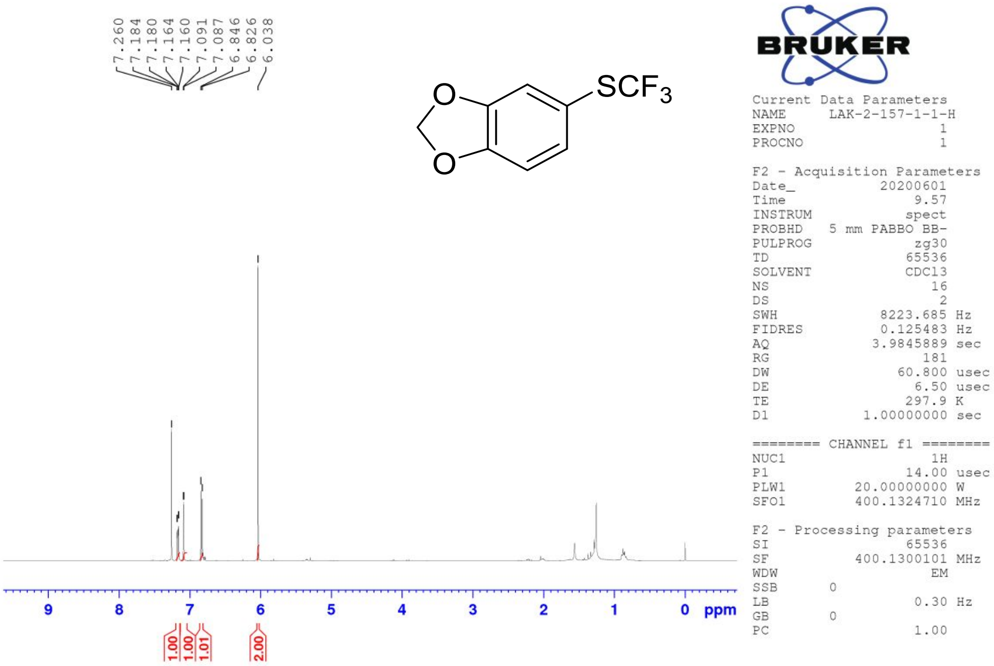

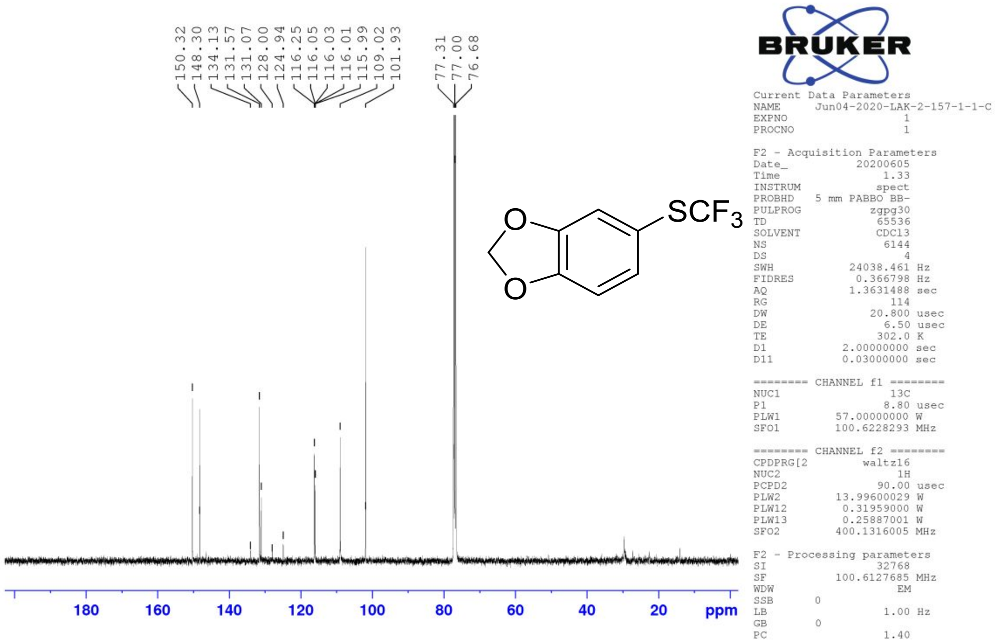
150.32
148.30
134.13
131.57
131.07
128.00
124.94
116.25
116.05
116.03
116.01
115.99
109.02
101.93
77.31
77.00
76.68
SCF3
O
O
BRUKER
Current Data Parameters
NAME Jun04-2020-LAK-2-157-1-1-C
EXPNO 1
PROCNO 1
F2 - Acquisition Parameters
Date_ 20200605
Time 1.33
INSTRUM spect
PROBHD 5 mm PABBO BB-
PULPROG zgpg30
TD 65536
SOLVENT CDCl3
NS 6144
DS 4
SWH 24038.461 Hz
FIDRES 0.366798 Hz
AQ 1.3631488 sec
RG 114
DW 20.800 usec
DE 6.50 usec
TE 302.0 K
D1 2.00000000 sec
D11 0.03000000 sec
======== CHANNEL f1 ========
NUC1 13C
P1 8.80 usec
PLW1 57.00000000 W
SFO1 100.6228293 MHz
======== CHANNEL f2 ========
CPDPRG[2 waltz16
NUC2 1H
PCPD2 90.00 usec
PLW2 13.99600029 W
PLW12 0.31959000 W
PLW13 0.25887001 W
SFO2 400.1316005 MHz
F2 - Processing parameters
SI 32768
SF 100.6127685 MHz
WDW EM
SSB 0
LB 1.00 Hz
GB 0
PC 1.40
180
160
140
120
100
80
60
40
20
ppm

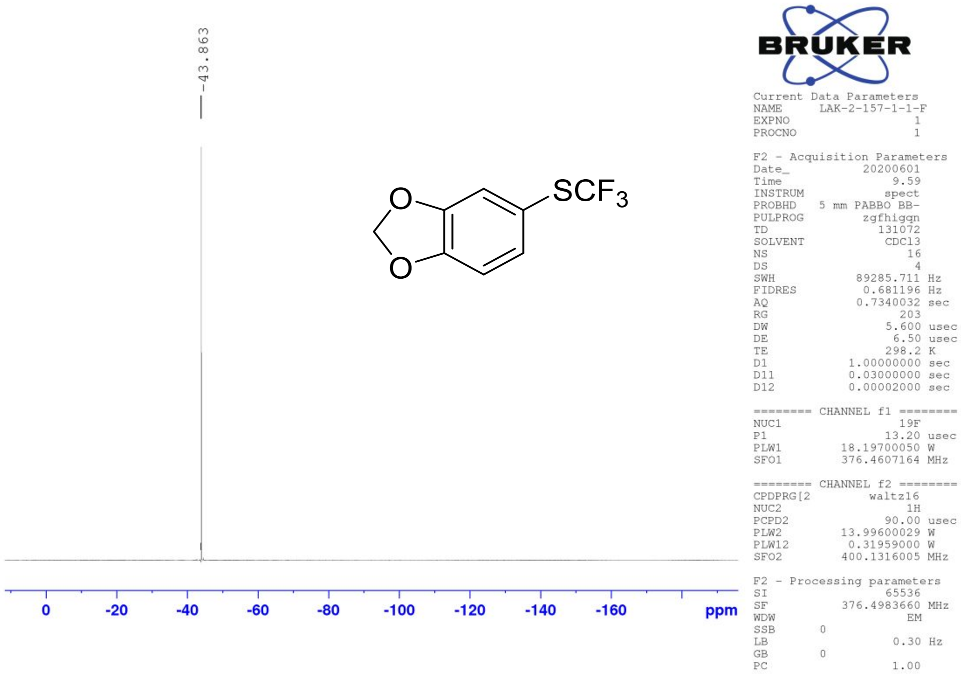
-43.863
SCF3
O
O
BRUKER
Current Data Parameters
NAME LAK-2-157-1-1-F
EXPNO 1
PROCNO 1
F2 - Acquisition Parameters
Date_ 20200601
Time 9.59
INSTRUM spect
PROBHD 5 mm PABBO BB-
PULPROG zgfhigqn
TD 131072
SOLVENT CDCl3
NS 16
DS 4
SWH 89285.711 Hz
FIDRES 0.681196 Hz
AQ 0.7340032 sec
RG 203
DW 5.600 usec
DE 6.50 usec
TE 298.2 K
D1 1.00000000 sec
D11 0.03000000 sec
D12 0.00002000 sec
======== CHANNEL f1 ========
NUC1 19F
P1 13.20 usec
PLW1 18.19700050 W
SFO1 376.4607164 MHz
======== CHANNEL f2 ========
CPDPRG[2 waltz16
NUC2 1H
PCPD2 90.00 usec
PLW2 13.99600029 W
PLW12 0.31959000 W
SFO2 400.1316005 MHz
F2 - Processing parameters
SI 65536
SF 376.4983660 MHz
WDW EM
SSB 0
LB 0.30 Hz
GB 0
PC 1.00
0
-20
-40
-60
-80
-100
-120
-140
-160
ppm

^{1}H, ^{13}C and ^{19}F NMR spectra of ***naphthalen-2-yl(trifluoromethyl)sulfane (3q)*** in $CDCl_3$

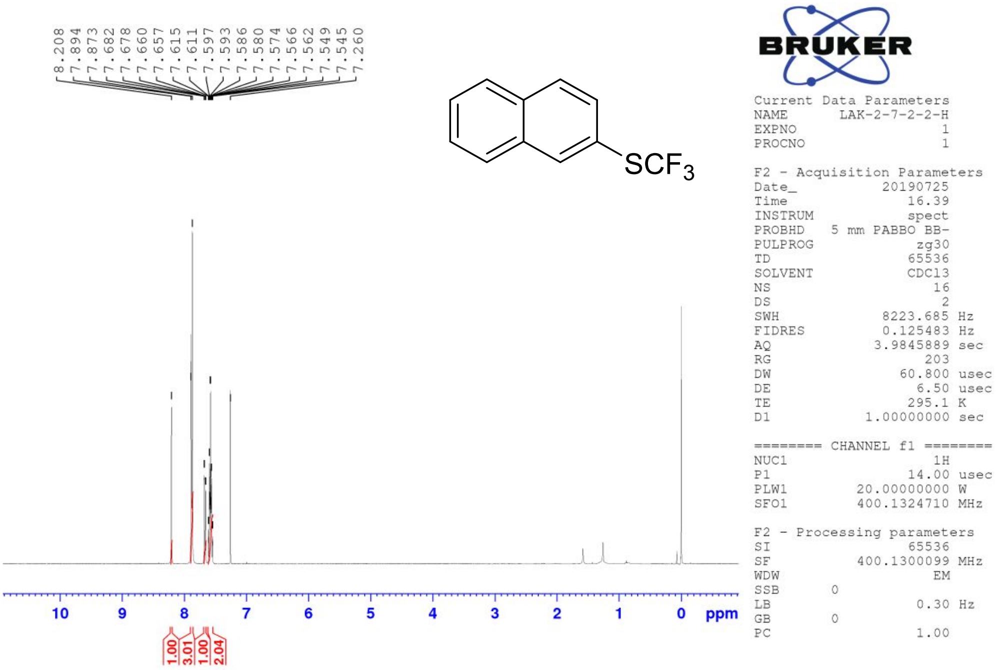

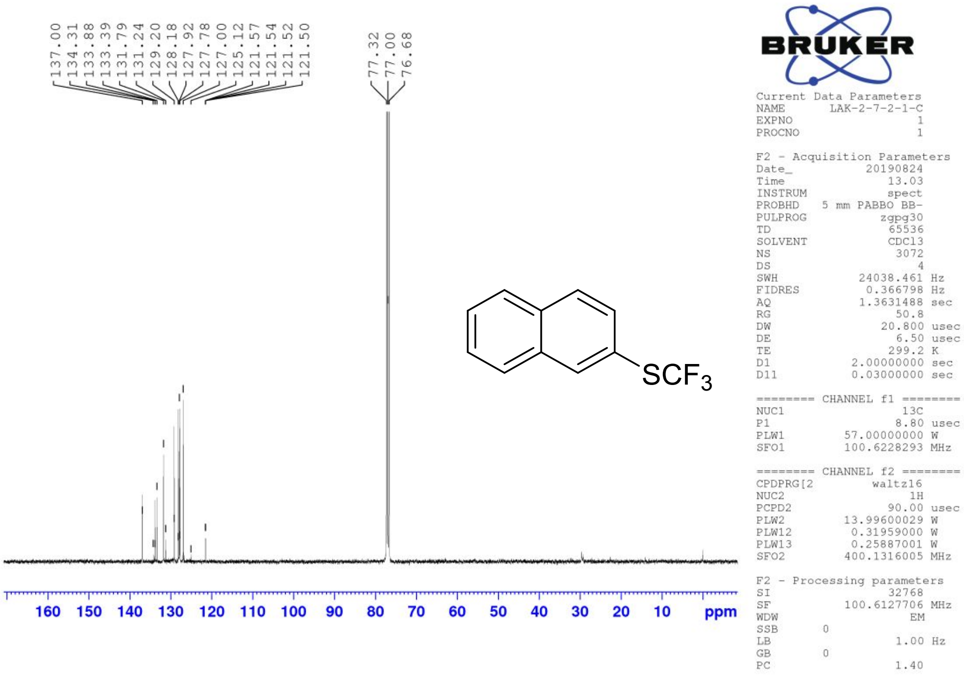

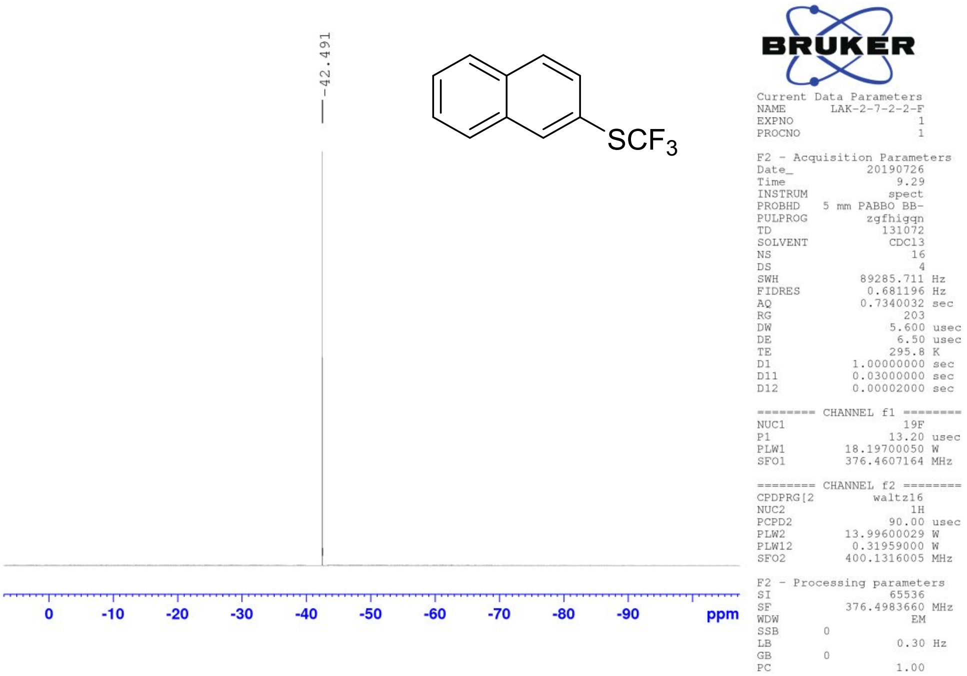

^{1}H, ^{13}C and ^{19}F NMR spectra of ***ethyl 5-((trifluoromethyl)thio)-2,3-dihydrobenzofuran-2-carboxylate (3r)*** in $CDCl_3$

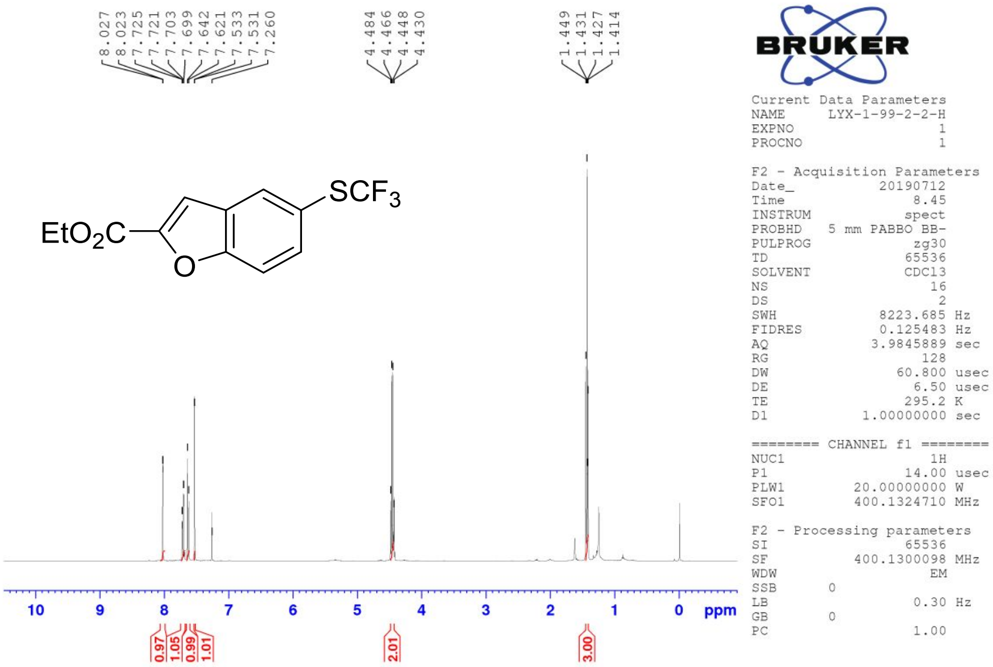

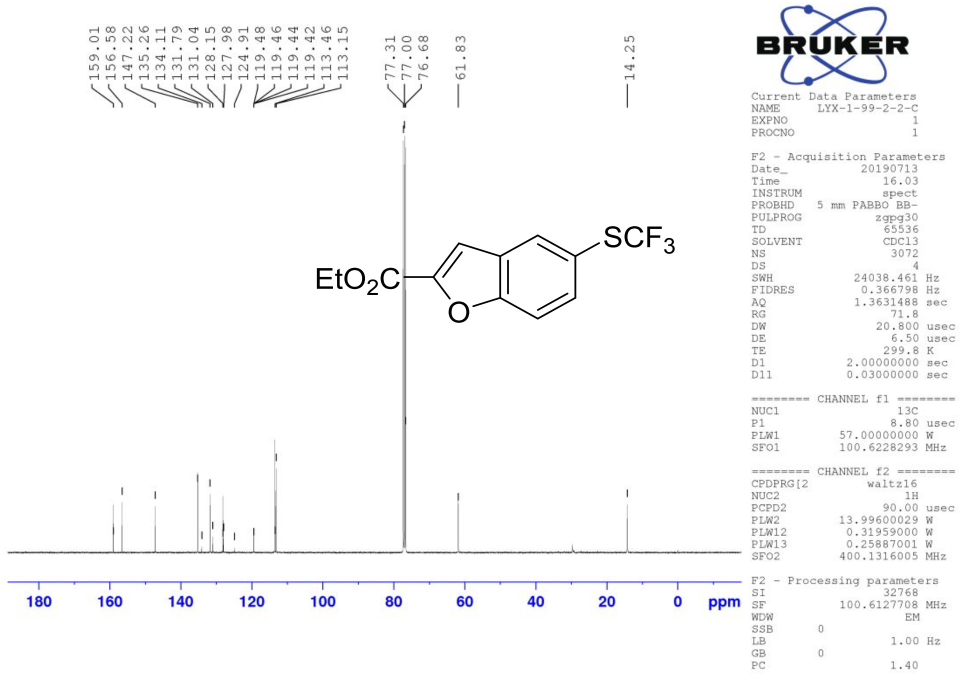
159.01
156.58
147.22
135.26
134.11
131.79
131.04
128.15
127.98
124.91
119.48
119.46
119.44
119.42
113.46
113.15
77.31
77.00
76.68
61.83
14.25
EtO2C
O
SCF3
180 160 140 120 100 80 60 40 20 0 ppm
BRUKER
Current Data Parameters
NAME LYX-1-99-2-2-C
EXPNO 1
PROCNO 1
F2 - Acquisition Parameters
Date_ 20190713
Time 16.03
INSTRUM spect
PROBHD 5 mm PABBO BB-
PULPROG zgpg30
TD 65536
SOLVENT CDCl3
NS 3072
DS 4
SWH 24038.461 Hz
FIDRES 0.366798 Hz
AQ 1.3631488 sec
RG 71.8
DW 20.800 usec
DE 6.50 usec
TE 299.8 K
D1 2.00000000 sec
D11 0.03000000 sec
======== CHANNEL f1 ========
NUC1 13C
P1 8.80 usec
PLW1 57.00000000 W
SFO1 100.6228293 MHz
======== CHANNEL f2 ========
CPDPRG[2 waltz16
NUC2 1H
PCPD2 90.00 usec
PLW2 13.99600029 W
PLW12 0.31959000 W
PLW13 0.25887001 W
SFO2 400.1316005 MHz
F2 - Processing parameters
SI 32768
SF 100.6127708 MHz
WDW EM
SSB 0
LB 1.00 Hz
GB 0
PC 1.40

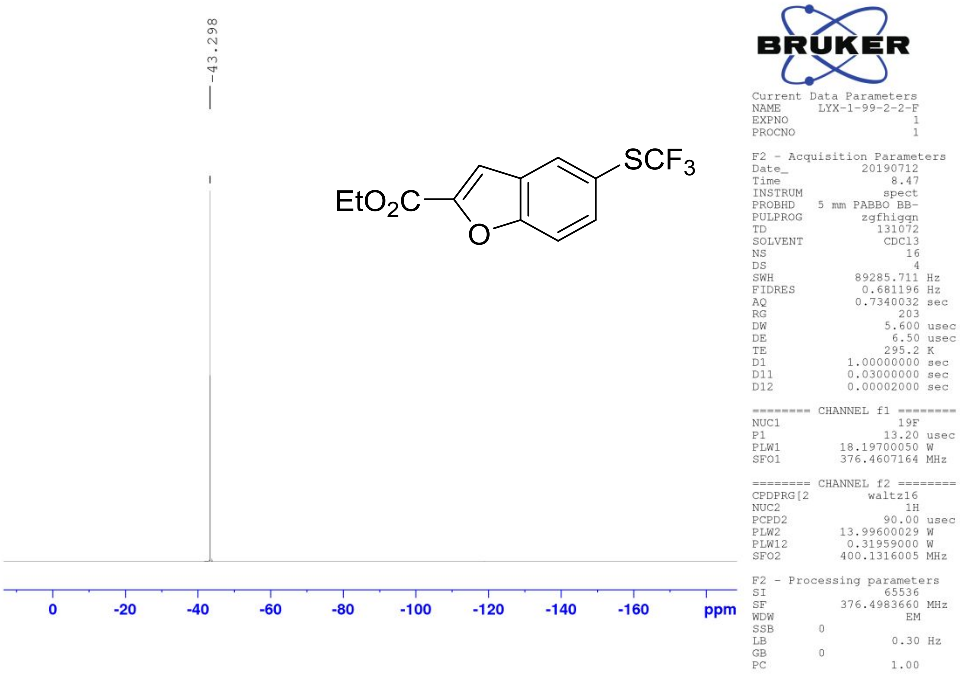
-43.298
EtO2C
O
SCF3
0 -20 -40 -60 -80 -100 -120 -140 -160 ppm
BRUKER
Current Data Parameters
NAME LYX-1-99-2-2-F
EXPNO 1
PROCNO 1
F2 - Acquisition Parameters
Date_ 20190712
Time 8.47
INSTRUM spect
PROBHD 5 mm PABBO BB-
PULPROG zgfhigqn
TD 131072
SOLVENT CDCl3
NS 16
DS 4
SWH 89285.711 Hz
FIDRES 0.681196 Hz
AQ 0.7340032 sec
RG 203
DW 5.600 usec
DE 6.50 usec
TE 295.2 K
D1 1.00000000 sec
D11 0.03000000 sec
D12 0.00002000 sec
======== CHANNEL f1 ========
NUC1 19F
P1 13.20 usec
PLW1 18.19700050 W
SFO1 376.4607164 MHz
======== CHANNEL f2 ========
CPDPRG[2 waltz16
NUC2 1H
PCPD2 90.00 usec
PLW2 13.99600029 W
PLW12 0.31959000 W
SFO2 400.1316005 MHz
F2 - Processing parameters
SI 65536
SF 376.4983660 MHz
WDW EM
SSB 0
LB 0.30 Hz
GB 0
PC 1.00

^{1}H, ^{13}C and ^{19}F NMR spectra of ***2-methyl-5-((trifluoromethyl)thio)benzo[d]thiazole (3s)*** in $CDCl_3$

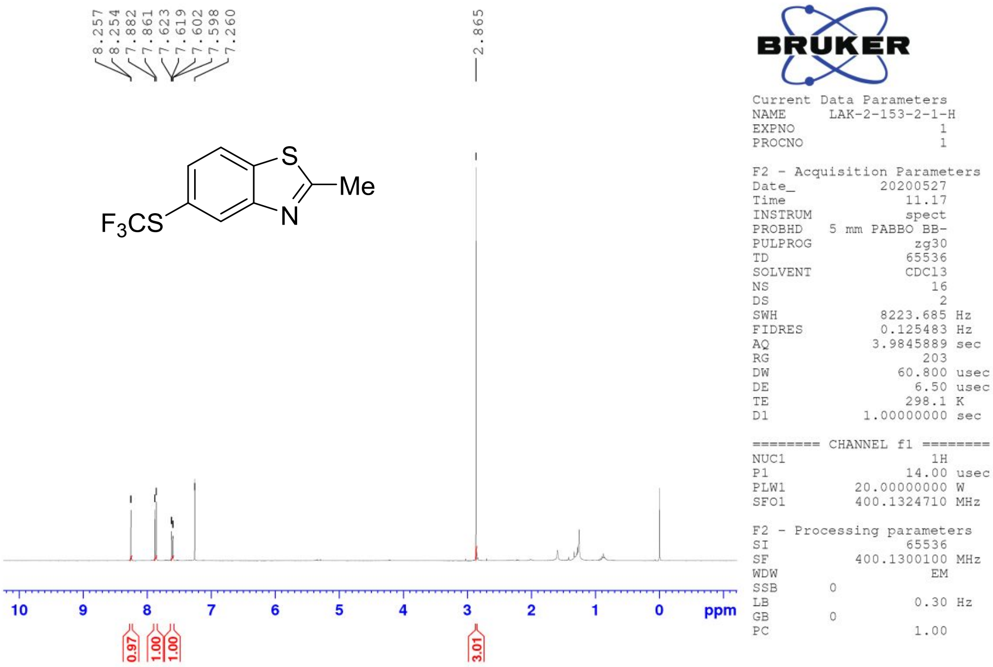

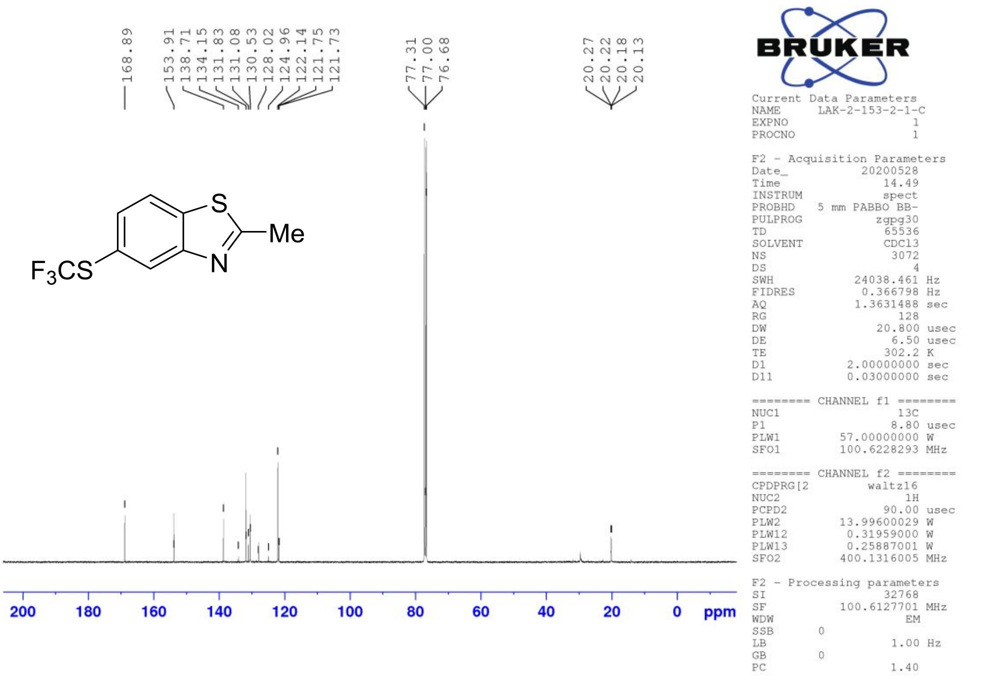

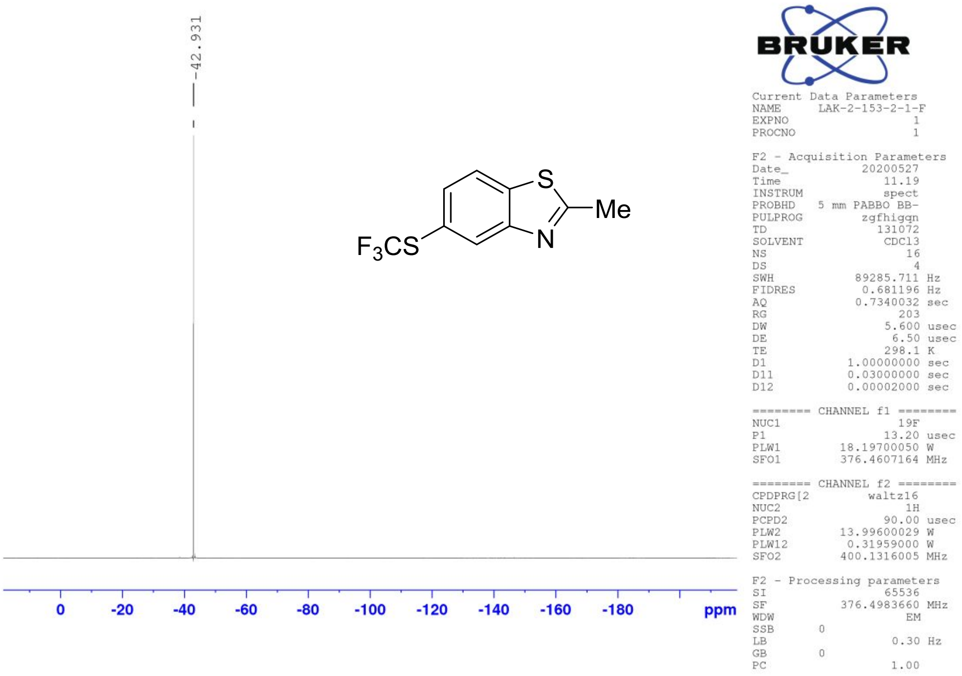

^{1}H, ^{13}C and ^{19}F NMR spectra of ***methyl 5-((trifluoromethyl)thio)nicotinate (3t)*** in $CDCl_3$

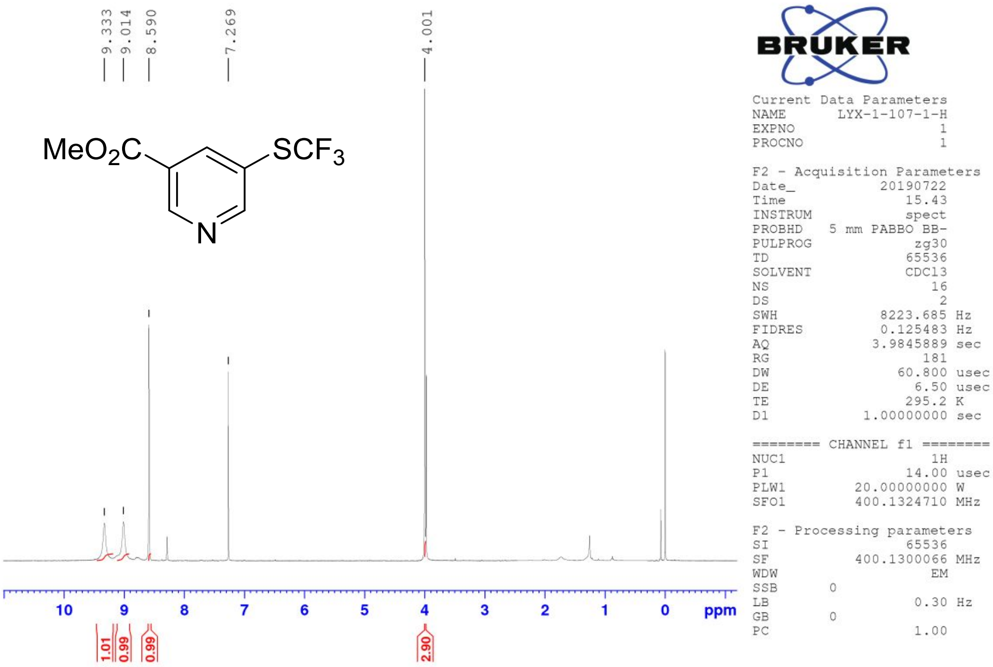

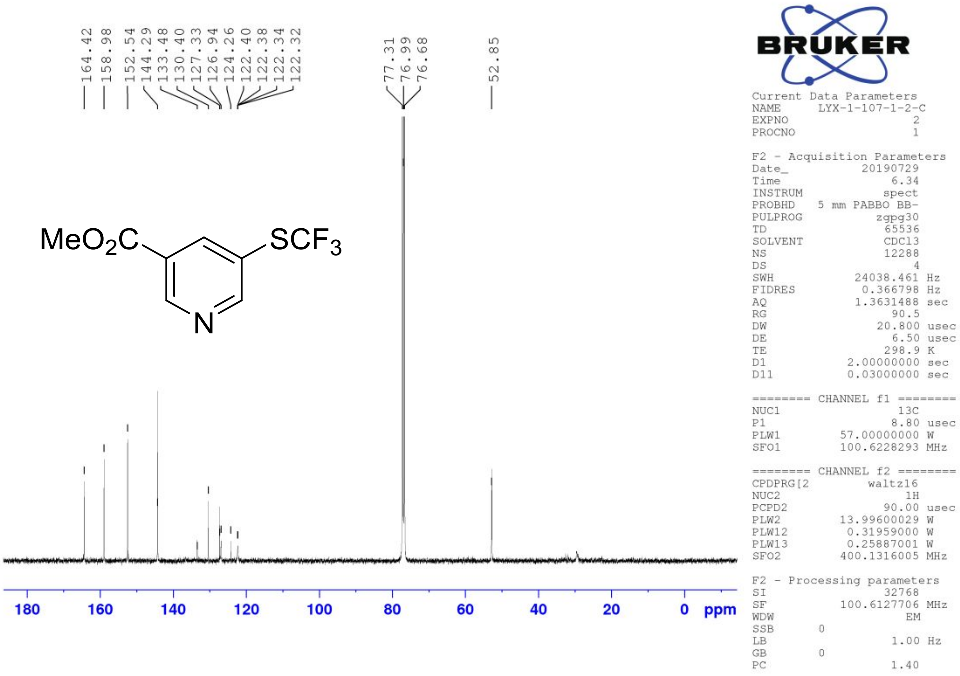
164.42
158.98
152.54
144.29
133.48
130.40
127.33
126.94
124.26
122.40
122.38
122.34
122.32
77.31
76.99
76.68
52.85
MeO2C
SCF3
N
180 160 140 120 100 80 60 40 20 0 ppm
BRUKER
Current Data Parameters
NAME LYX-1-107-1-2-C
EXPNO 2
PROCNO 1
F2 - Acquisition Parameters
Date_ 20190729
Time 6.34
INSTRUM spect
PROBHD 5 mm PABBO BB-
PULPROG zgpg30
TD 65536
SOLVENT CDCl3
NS 12288
DS 4
SWH 24038.461 Hz
FIDRES 0.366798 Hz
AQ 1.3631488 sec
RG 90.5
DW 20.800 usec
DE 6.50 usec
TE 298.9 K
D1 2.00000000 sec
D11 0.03000000 sec
======== CHANNEL f1 ========
NUC1 13C
P1 8.80 usec
PLW1 57.00000000 W
SFO1 100.6228293 MHz
======== CHANNEL f2 ========
CPDPRG[2 waltz16
NUC2 1H
PCPD2 90.00 usec
PLW2 13.99600029 W
PLW12 0.31959000 W
PLW13 0.25887001 W
SFO2 400.1316005 MHz
F2 - Processing parameters
SI 32768
SF 100.6127706 MHz
WDW EM
SSB 0
LB 1.00 Hz
GB 0
PC 1.40

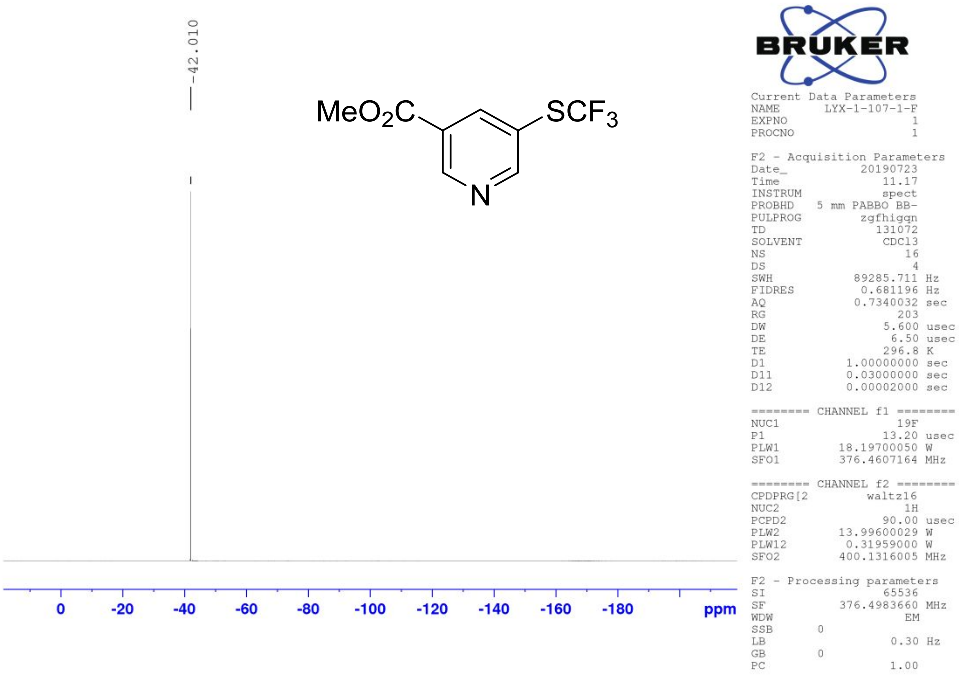
-42.010
MeO2C
SCF3
N
0 -20 -40 -60 -80 -100 -120 -140 -160 -180 ppm
BRUKER
Current Data Parameters
NAME LYX-1-107-1-F
EXPNO 1
PROCNO 1
F2 - Acquisition Parameters
Date_ 20190723
Time 11.17
INSTRUM spect
PROBHD 5 mm PABBO BB-
PULPROG zgfhigqn
TD 131072
SOLVENT CDCl3
NS 16
DS 4
SWH 89285.711 Hz
FIDRES 0.681196 Hz
AQ 0.7340032 sec
RG 203
DW 5.600 usec
DE 6.50 usec
TE 296.8 K
D1 1.00000000 sec
D11 0.03000000 sec
D12 0.00002000 sec
======== CHANNEL f1 ========
NUC1 19F
P1 13.20 usec
PLW1 18.19700050 W
SFO1 376.4607164 MHz
======== CHANNEL f2 ========
CPDPRG[2 waltz16
NUC2 1H
PCPD2 90.00 usec
PLW2 13.99600029 W
PLW12 0.31959000 W
SFO2 400.1316005 MHz
F2 - Processing parameters
SI 65536
SF 376.4983660 MHz
WDW EM
SSB 0
LB 0.30 Hz
GB 0
PC 1.00

^{1}H, ^{13}C and ^{19}F NMR spectra of ***7-((trifluoromethyl)thio)quinoline (3u)*** in $CDCl_3$

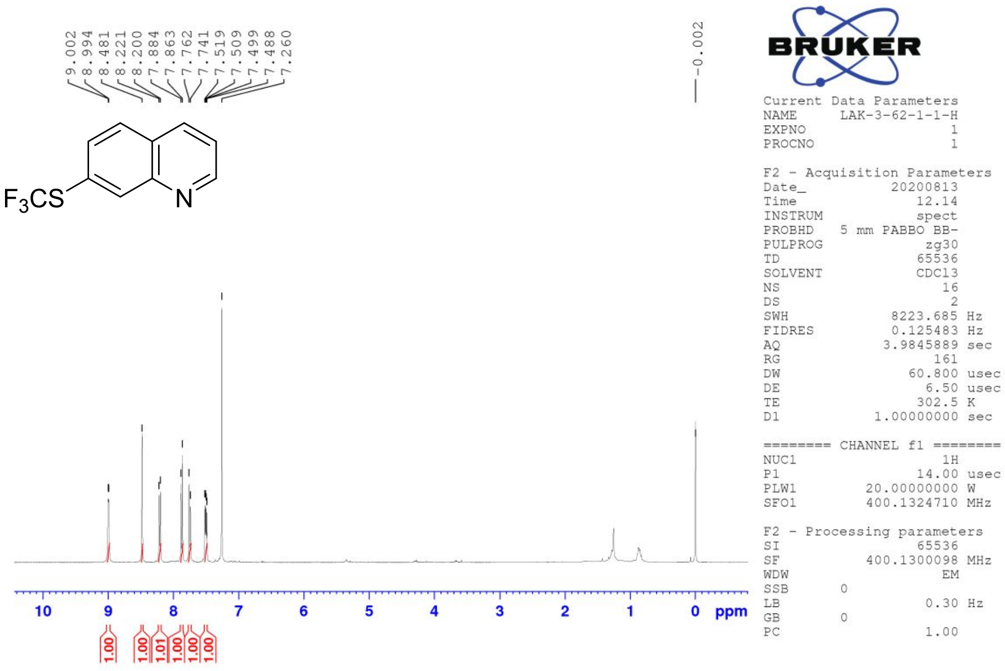

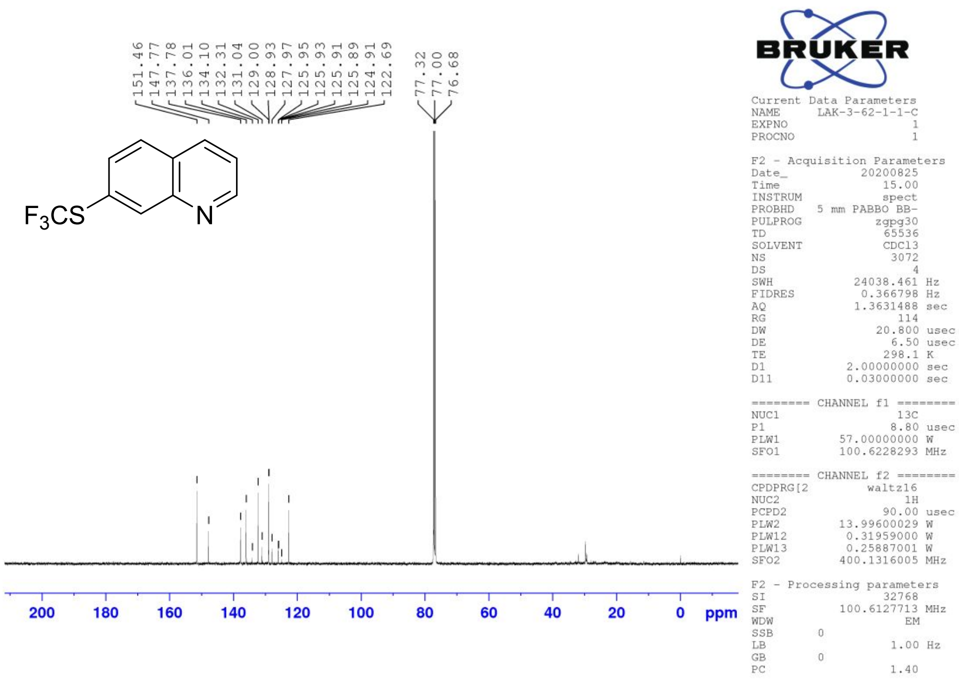

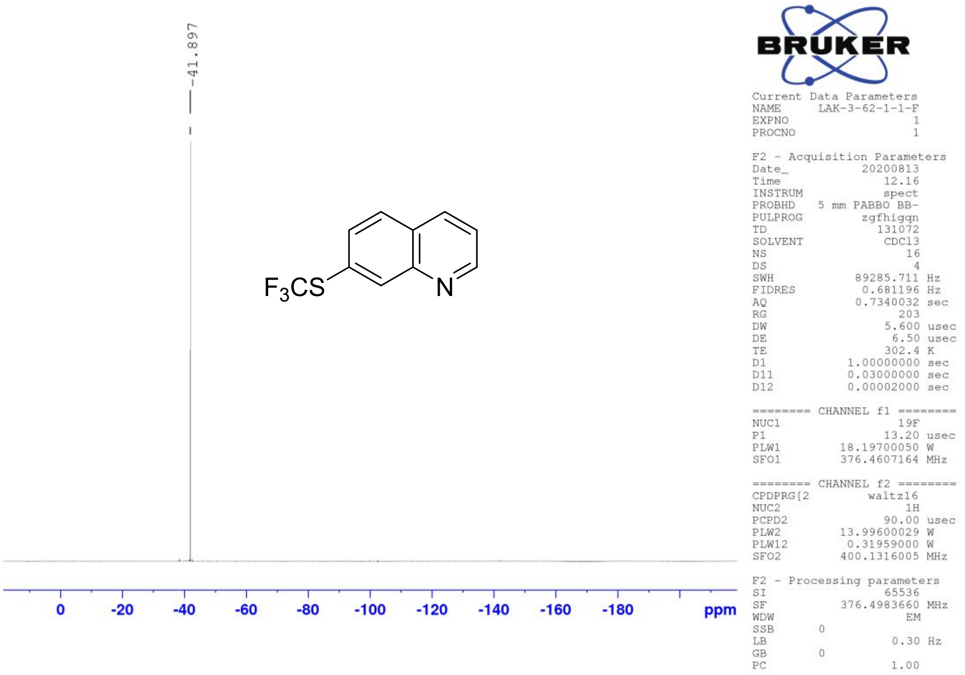

^{1}H, ^{13}C and ^{19}F NMR spectra of ***4-methyl-7-((trifluoromethyl)thio)-2H-chromen-2-one (3v)*** in $CDCl_3$

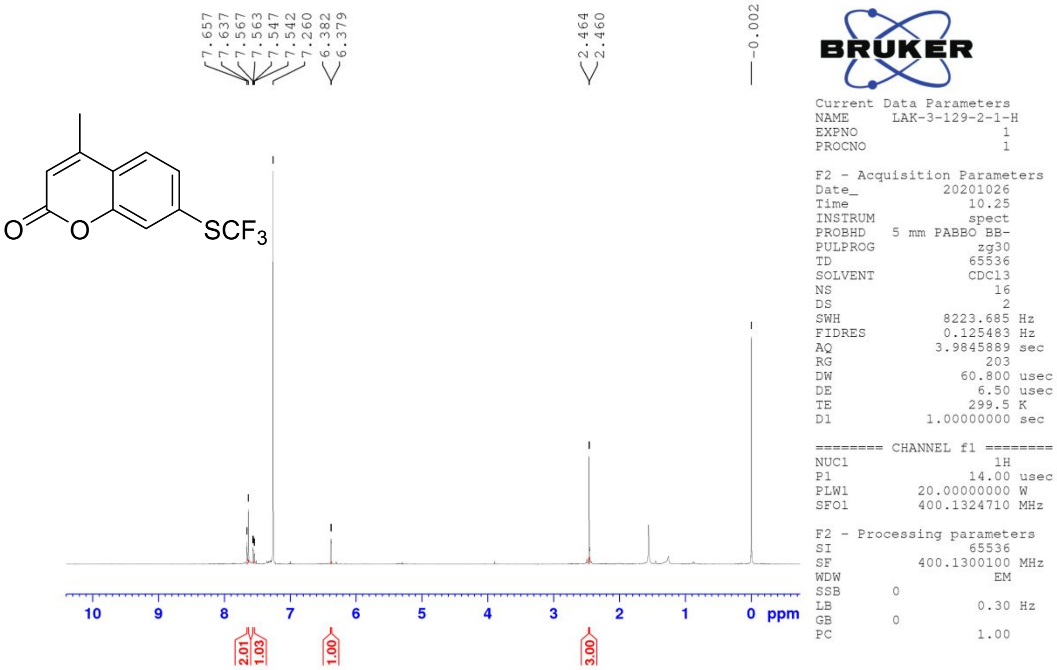

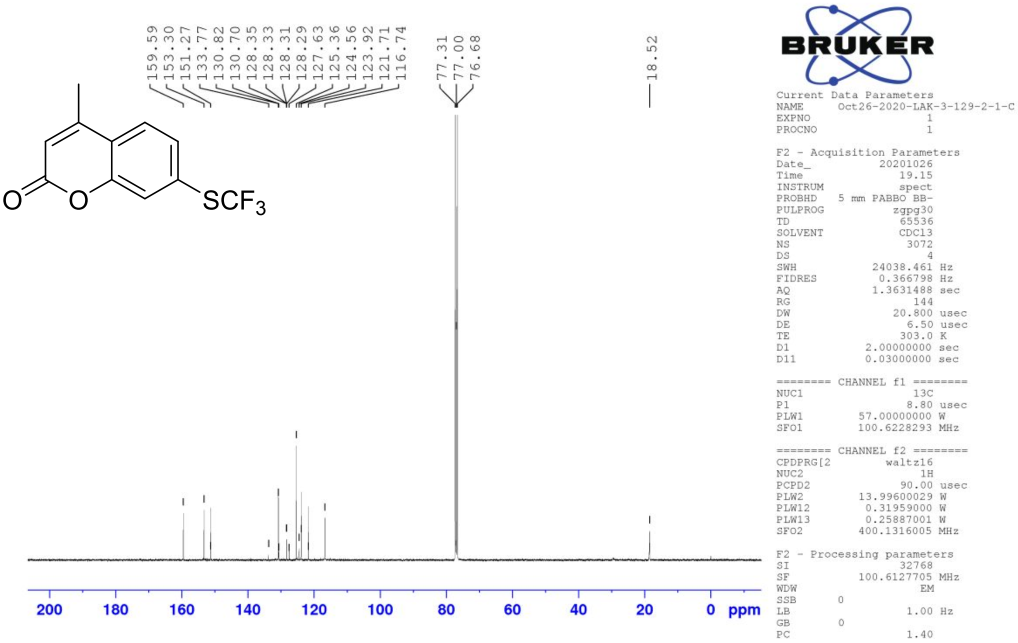
159.59
153.30
151.27
133.77
130.82
130.70
128.35
128.33
128.31
128.29
127.63
125.36
124.56
123.92
121.71
116.74
77.31
77.00
76.68
18.52
O
O
SCF3
200
180
160
140
120
100
80
60
40
20
0
ppm
Current Data Parameters
NAME Oct26-2020-LAK-3-129-2-1-C
EXPNO 1
PROCNO 1
F2 - Acquisition Parameters
Date_ 20201026
Time 19.15
INSTRUM spect
PROBHD 5 mm PABBO BB-
PULPROG zgpg30
TD 65536
SOLVENT CDCl3
NS 3072
DS 4
SWH 24038.461 Hz
FIDRES 0.366798 Hz
AQ 1.3631488 sec
RG 144
DW 20.800 usec
DE 6.50 usec
TE 303.0 K
D1 2.00000000 sec
D11 0.03000000 sec
======== CHANNEL f1 ========
NUC1 13C
P1 8.80 usec
PLW1 57.00000000 W
SFO1 100.6228293 MHz
======== CHANNEL f2 ========
CPDPRG[2 waltz16
NUC2 1H
PCPD2 90.00 usec
PLW2 13.99600029 W
PLW12 0.31959000 W
PLW13 0.25887001 W
SFO2 400.1316005 MHz
F2 - Processing parameters
SI 32768
SF 100.6127705 MHz
WDW EM
SSB 0
LB 1.00 Hz
GB 0
PC 1.40

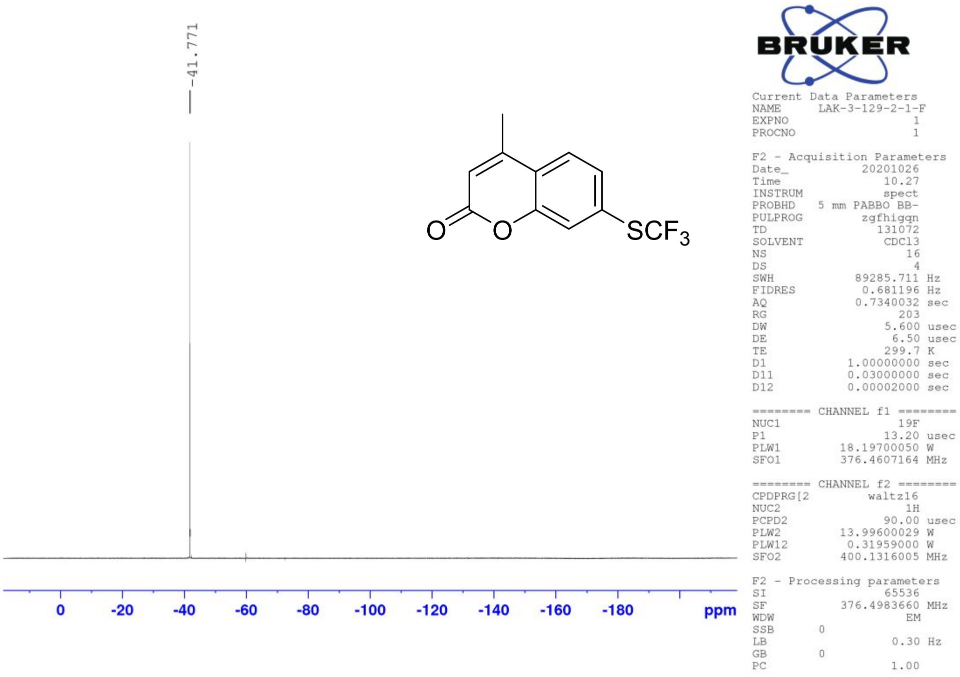
-41.771
O
O
SCF3
0
-20
-40
-60
-80
-100
-120
-140
-160
-180
ppm
Current Data Parameters
NAME LAK-3-129-2-1-F
EXPNO 1
PROCNO 1
F2 - Acquisition Parameters
Date_ 20201026
Time 10.27
INSTRUM spect
PROBHD 5 mm PABBO BB-
PULPROG zgfhigqn
TD 131072
SOLVENT CDCl3
NS 16
DS 4
SWH 89285.711 Hz
FIDRES 0.681196 Hz
AQ 0.7340032 sec
RG 203
DW 5.600 usec
DE 6.50 usec
TE 299.7 K
D1 1.00000000 sec
D11 0.03000000 sec
D12 0.00002000 sec
======== CHANNEL f1 ========
NUC1 19F
P1 13.20 usec
PLW1 18.19700050 W
SFO1 376.4607164 MHz
======== CHANNEL f2 ========
CPDPRG[2 waltz16
NUC2 1H
PCPD2 90.00 usec
PLW2 13.99600029 W
PLW12 0.31959000 W
SFO2 400.1316005 MHz
F2 - Processing parameters
SI 65536
SF 376.4983660 MHz
WDW EM
SSB 0
LB 0.30 Hz
GB 0
PC 1.00

^{1}H, ^{13}C and ^{19}F NMR spectra of ***ethyl-3-((trifluoromethyl)thio)-9H-carbazole (3w)*** in $CDCl_3$

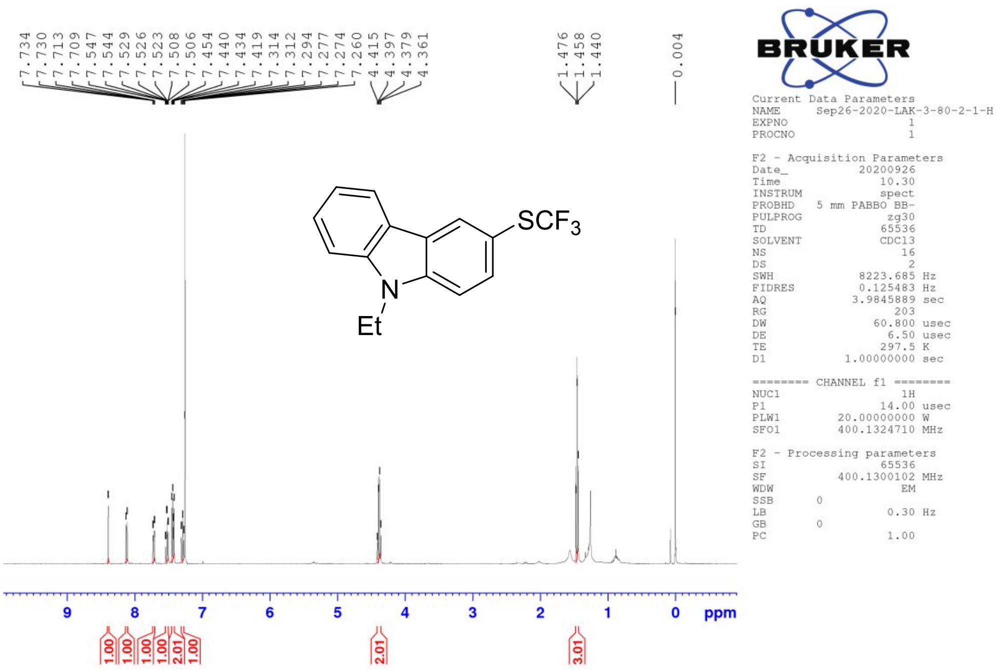

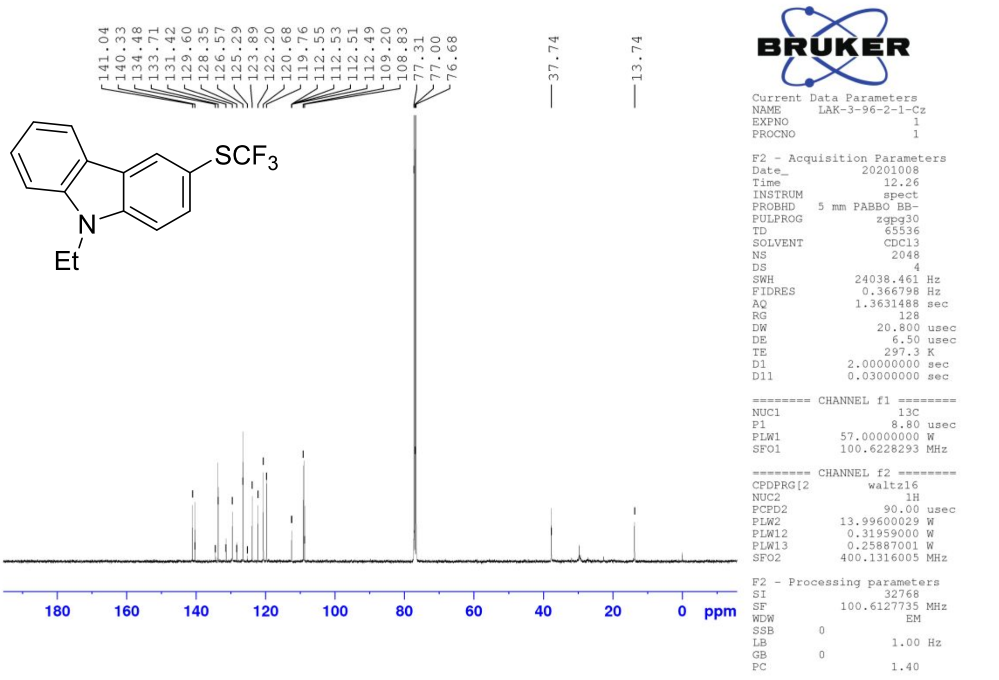

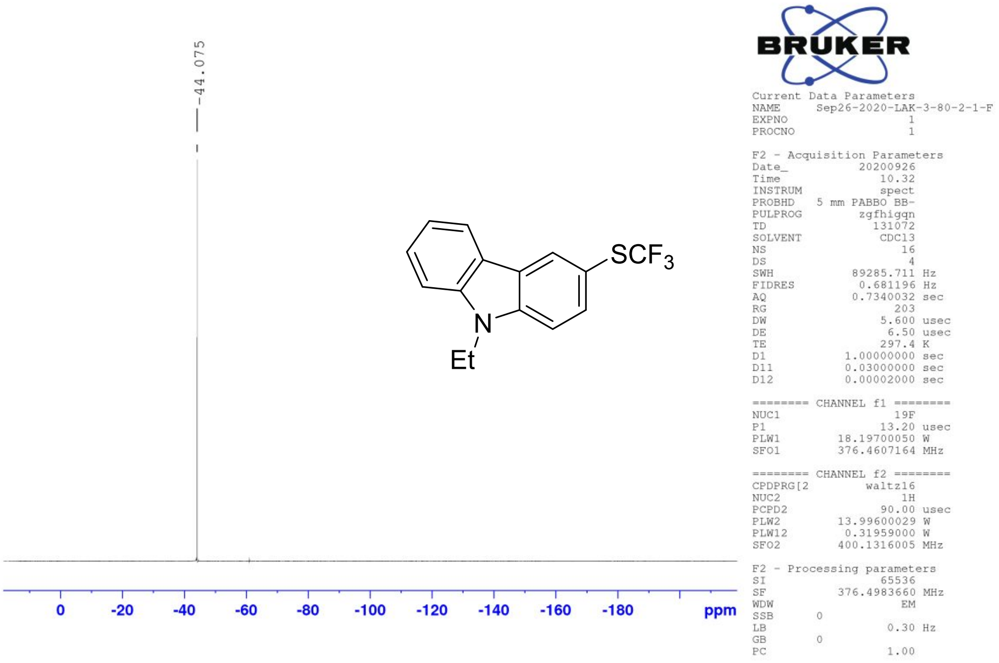

^{1}H, ^{13}C and ^{19}F NMR spectra of ***5-((trifluoromethyl)thio)benzo[d]thiazole (3x)*** in $CDCl_3$

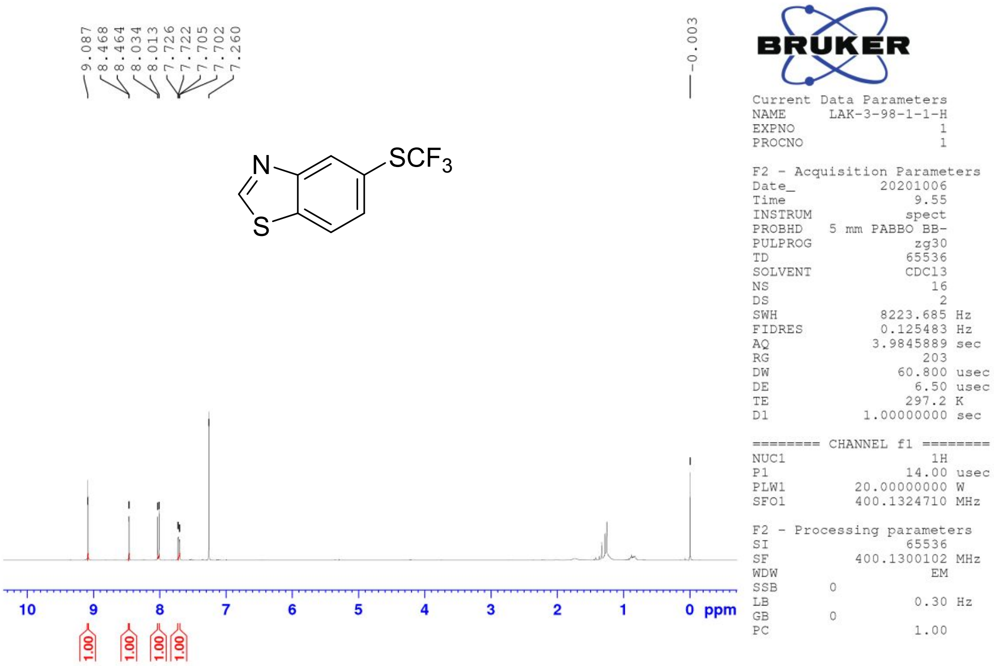

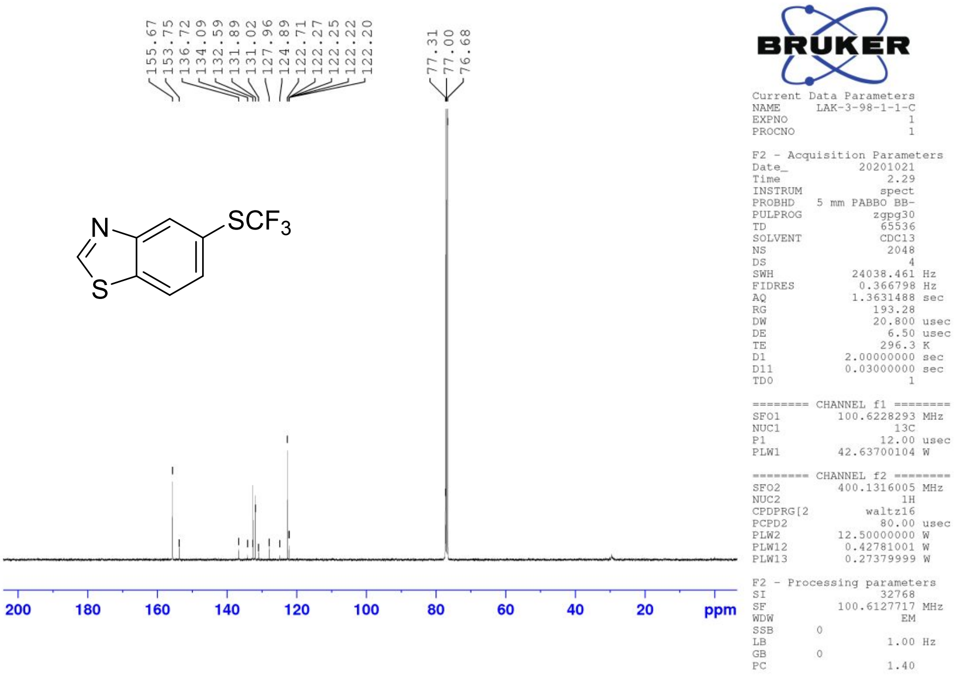
155.67
153.75
136.72
134.09
132.59
131.89
131.02
127.96
124.89
122.71
122.27
122.25
122.22
122.20
77.31
77.00
76.68
N
S
SCF3
200 180 160 140 120 100 80 60 40 20 ppm
BRUKER
Current Data Parameters
NAME LAK-3-98-1-1-C
EXPNO 1
PROCNO 1
F2 - Acquisition Parameters
Date_ 20201021
Time 2.29
INSTRUM spect
PROBHD 5 mm PABBO BB-
PULPROG zgpg30
TD 65536
SOLVENT CDCl3
NS 2048
DS 4
SWH 24038.461 Hz
FIDRES 0.366798 Hz
AQ 1.3631488 sec
RG 193.28
DW 20.800 usec
DE 6.50 usec
TE 296.3 K
D1 2.00000000 sec
D11 0.03000000 sec
TD0 1
======== CHANNEL f1 ========
SFO1 100.6228293 MHz
NUC1 13C
P1 12.00 usec
PLW1 42.63700104 W
======== CHANNEL f2 ========
SFO2 400.1316005 MHz
NUC2 1H
CPDPRG[2 waltz16
PCPD2 80.00 usec
PLW2 12.50000000 W
PLW12 0.42781001 W
PLW13 0.27379999 W
F2 - Processing parameters
SI 32768
SF 100.6127717 MHz
WDW EM
SSB 0
LB 1.00 Hz
GB 0
PC 1.40

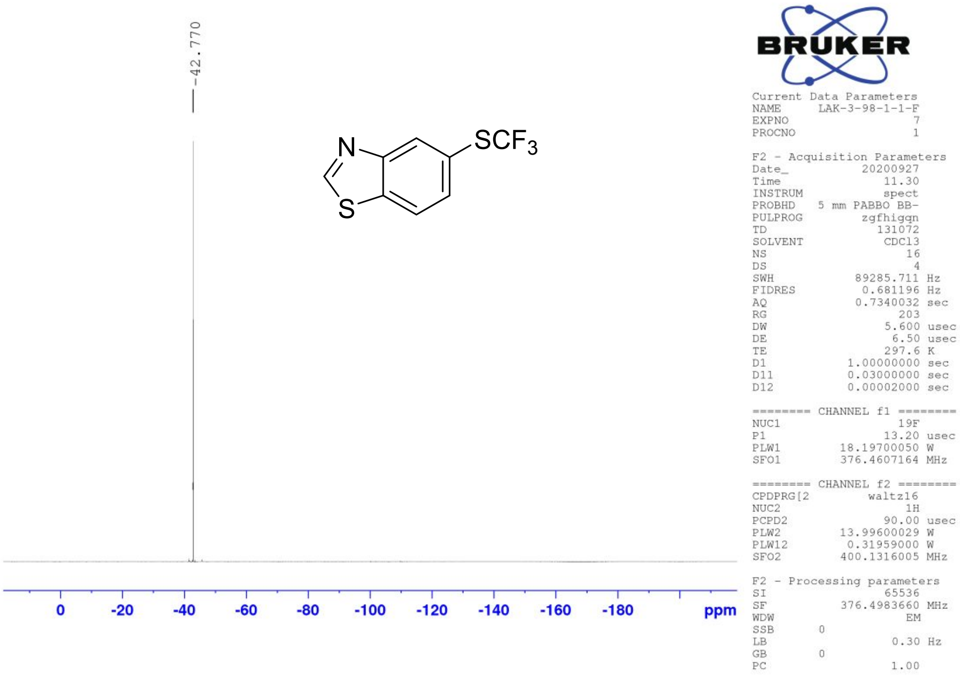
-42.770
N
S
SCF3
0 -20 -40 -60 -80 -100 -120 -140 -160 -180 ppm
BRUKER
Current Data Parameters
NAME LAK-3-98-1-1-F
EXPNO 7
PROCNO 1
F2 - Acquisition Parameters
Date_ 20200927
Time 11.30
INSTRUM spect
PROBHD 5 mm PABBO BB-
PULPROG zgfhigqn
TD 131072
SOLVENT CDCl3
NS 16
DS 4
SWH 89285.711 Hz
FIDRES 0.681196 Hz
AQ 0.7340032 sec
RG 203
DW 5.600 usec
DE 6.50 usec
TE 297.6 K
D1 1.00000000 sec
D11 0.03000000 sec
D12 0.00002000 sec
======== CHANNEL f1 ========
NUC1 19F
P1 13.20 usec
PLW1 18.19700050 W
SFO1 376.4607164 MHz
======== CHANNEL f2 ========
CPDPRG[2 waltz16
NUC2 1H
PCPD2 90.00 usec
PLW2 13.99600029 W
PLW12 0.31959000 W
SFO2 400.1316005 MHz
F2 - Processing parameters
SI 65536
SF 376.4983660 MHz
WDW EM
SSB 0
LB 0.30 Hz
GB 0
PC 1.00
